# Supplementary material for: Control of flagellar gene expression by a chemotaxis receptor-like regulator in pathogenic Escherichia coli
Source: EMBO J. 2025 Oct 13;44(22):6675–703. doi: 10.1038/s44318-025-00595-x (PMC12623472; doi:10.1038/s44318-025-00595-x)
Supplement: Supplementary file 1 — Appendix [file 44318_2025_595_MOESM1_ESM.pdf]

Appendix for:

Control of flagellar gene expression by a chemotaxis receptor-like regulator in pathogenic *Escherichia coli*

Jae-Woo Lee[1#], Liyun Wang[1#], Sarah L. Comer[2], Remy Colin[1], Mollie M Gidney[2], Leanid Laganenka[3], Wolf-Dietrich Hardt[3], Maria Hadjifrangiskou[2], Victor Sourjik[1*]

[1]Max Planck Institute for Terrestrial Microbiology and Center for Synthetic Microbiology (SYNMIKRO), Marburg, Germany

[2]Department of Pathology, Microbiology & Immunology, Vanderbilt University Medical Center, Nashville, Tennessee, United States of America

[3]Institute of Microbiology, D-BIOL, ETH Zurich, Zurich, Switzerland

[#]These authors equally contributed to this work.

[*]Corresponding author: Victor Sourjik, victor.sourjik@mpi-marburg.mpg.de

Table of Contents

Appendix Table S1 3

Appendix Table S2 5

Appendix Table S3 7

Appendix Table S4 9

Appendix Table S5 11

Appendix Table S6 13

Appendix Table S7 14

Appendix Figure S1..15

Appendix Figure S2..16

Appendix Figure S3..17

Appendix Figure S4..19

Appendix Figure S5..20

Appendix Figure S6..21

Appendix Figure S7..23

Appendix Figure S8..25

Appendix Figure S9..28

Appendix Figure S10..29

Appendix Figure S11..30

Appendix Table S1

List of primers used in this study

Primer	Sequence (5’ -> 3’)
S13 *tls* deletion-F	atgaaaaaactggcacttattttgtttatggg
S13 *tls* deletion-R	gccagggctgtaaaattccttcgggttg
fliA deletion-F	gagactgacggcaacgccaaattgcctgatg
fliA deletion-R	atcactctgcgttttgcaatgtaaatcaccg
pTrc99A-Tls-gibson for vector-F	gctacggtgtgatctagagtcgacctgcaggcatg
pTrc99A-Tls-gibson for vector-R	acccatggcacactccttcactagtacatggtctg
pTrc99A-Tls-gibson for Insert-F	ctagtgaaggagtgtgccatgggtatgaatgtcagaatcgtg
pTrc99A-Tls-gibson for Insert-R	gactctagatcacaccgtagcaaaactaactgg
pTrc99A-TlsN-TrgΔN-gibson for vector-F	gctacggtgtgatctagagtcgacctgcaggcatg
pTrc99A-TlsN-TrgΔN-gibson for vector-R	cgagagtaacacgattctgacattcatacccatgg
pTrc99A-TlsN-TrgΔN-gibson for insert-F	tgtcagaatcgtgttactctcgcaagggcgtggatttgccg
pTrc99A-TlsN-TrgΔN-gibson for insert-R	gactctagatcacaccgtagcgaaactaactgg
pTrc99A-TlsN-TarΔN-gibson for vector-F	gaaacattttgatctagagtcgacctgcaggcatg
pTrc99A-TlsN-TarΔN-gibson for vector-R	cgagagtaacacgattctgacattcatacccatgg
pTrc99A-TlsN-TarΔN-gibson for insert-F	tgtcagaatcgtgttactctcgcagggccgtggttttgccg
pTrc99A-TlsN-TarΔN-gibson for insert-R	gactctagatcaaaatgtttcccagtttgg
pTrc99A-TlsN-TsrΔN-gibson for vector-F	gaaacattttaatctagagtcgacctgcaggcatg
pTrc99A-TlsN-TsrΔN-gibson for vector-R	cgagagtaacacgattctgacattcatacccatgg
pTrc99A-TlsN-TsrΔN-gibson for insert-F	tgtcagaatcgtgttactctcgcaagggcgcggttttgcgg
pTrc99A-TlsN-TsrΔN-gibson for insert-R	gactctagattaaaatgtttcccagttctcctcg
pTrc99A-TlsN-TapΔN-gibson for vector-F	gtggtatcctgatctagagtcgacctgcaggcatg
pTrc99A-TlsN-TapΔN-gibson for vector-R	cgagagtaacacgattctgacattcatacccatgg
pTrc99A-TlsN-TapΔN-gibson for insert-F	tgtcagaatcgtgttactctcgcaggggcgtggttttgcgg
pTrc99A-TlsN-TapΔN-gibson for insert-R	gactctagatcaggataccactggcgcaatttgtaactg
pTrc99A-TlsN-AerΔN-gibson for vector-F	gtactgcattaatctagagtcgacctgcaggcatg
pTrc99A-TlsN-AerΔN-gibson for vector-R	cgagagtaacacgattctgacattcatacccatgg
pTrc99A-TlsN-AerΔN-gibson for insert-F	tgtcagaatcgtgttactctcgcagggcaaaggttttgcag
pTrc99A-TlsN-AerΔN-gibson for insert-R	gactctagattaatgcagtaccgtcaccgcg
pTrc99A-TlsN-mutagenesis-F	tgatctagagtcgacctgcaggcatg
pTrc99A-TlsN-mutagenesis-R	cgagagtaacacgattctgacattcatacccatgg
pTrc99A-Tls-sfGFP-gibson for vector-F	ggcggaggtggatcttccaagggtgaagagc
pTrc99A-Tls-sfGFP-gibson for vector-R	acccatggcacactccttcactagtacatggtctg
pTrc99A-Tls-sfGFP-gibson for insert-F	ctagtgaaggagtgtgccatgggtatgaatgtcagaatcgtg
pTrc99A-Tls-sfGFP-gibson for insert-R	cttggaagatccacctccgcccaccgtagcaaaactaactgg
pTrc99A-TlsN-sfGFP-mutagenesis-F	ggcggaggtggatcttccaagggtgaagagc
pTrc99A-TlsN-sfGFP-mutagenesis-R	cgagagtaacacgattctgacattcatacccatgg

pTrc99A-TlsN-sfGFP(N149Y)-mutagenesis-F	caactcgcattatgtgtacattac
pTrc99A-TlsN-sfGFP(N149Y)-mutagenesis-R	aaattatactcaagtttatggc
pTrc99A-TlsN-sfGFP(R206A)-mutagenesis-F	cacacagtctgcgttgtcaaaagatccg
pTrc99A-TlsN-sfGFP(R206A)-mutagenesis-R	cttaagtagtgattatcgg
pTrc99A-TlsN-mCherry-mutagenesis-F	tctcgggcggaggtggatctgtgagcaagggcgaggagg
pTrc99A-TlsN-mCherry-mutagenesis-R	gtaacacgattctgacattcatacccatggcacactcc
pTrc99A-$(Gly)_8$-mCherry-mutagenesis-F	gtggaggcggaggtggatctgtgagcaagggcgaggagg
pTrc99A-$(Gly)_8$-mCherry-mutagenesis-R	ctccgcctccacctccgcccatacccatggcacactcc
pTrc99A-TlsN (R4D)-mCherry-mutagenesis-F	tctcgggcggaggtggatctgtgagcaagggcgaggagg
pTrc99A-TlsN (R4D)-mCherry-mutagenesis-R	gtaacacgatgtcgacattcatacccatggcacactcc
pTrc99A-TlsN (R4K)-mCherry-mutagenesis-F	tctcgggcggaggtggatctgtgagcaagggcgaggagg
pTrc99A-TlsN (R4K)-mCherry-mutagenesis-R	gtaacacgatcttgacattcatacccatggcacactccttcactag
pTrc99A-TlsN*-mCherry-mutagenesis-F	ttactctcgggcggaggtggatctgtgagcaagggcgaggagg
pTrc99A-TlsN*-mCherry-mutagenesis-R	cgaatgattaaactgactaaaccgcatacccatggcacactcc
pTrc99A-Tls-mCherry-gibson for vector-F	ctgtacaagtaatctagagtcgacctgcaggcatg
pTrc99A-Tls-mCherry-gibson for vector-R	agatccacctccgcccaccgtagcaaaactaactg
pTrc99A-Tls-mCherry-gibson for insert-F	acggtgggcggaggtggatctgtgagcaagggcgag
pTrc99A-Tls-mCherry-gibson for insert-R	gactctagattacttgtacagctcgtccatgccg
pTrc99A-mCherry-Tls-gibson for vector-F	ggcggaggtggatctaatgtcagaatcgtgttactctcg
pTrc99A-mCherry-Tls-gibson for vector-R	acccatggcacactccttcactagtacatggtctg
pTrc99A-mCherry-Tls-gibson for insert-F	ctagtgaaggagtgtgccatgggtatggtgagcaagggcgag
pTrc99A-mCherry-Tls-gibson for insert-R	gacattagatccacctccgcccttgtacagctcgtccatg
pTrc99A-Trg-gibson for vector-F	gctacggtgtgatctagagtcgacctgcaggcatg
pTrc99A-Trg-gibson for vector-R	acccatggcacactccttcactagtacatggtctg
pTrc99A-Trg-gibson for insert-F	ctagtgaaggagtgtgccatgggtatgaatacaactccctcacag
pTrc99A-Trg-gibson for insert-R	gactctagatcacaccgtagcgaaactaactgg
pBAD33RBS-FlhDC-sfGFP-enzyme cut-F (Sac1)	aaaaaagagctcatgcatacctccgagttgctg
pBAD33RBS-FlhDC-sfGFP-enzyme cut-R (HindIII)	ttttttaagcttttacgaccccttataaagc
pBAD33RBS-FlhDC-sfGFP-mutagenesis-F	gtcgcggacgcctgatagaactttatgaagaactgcgcggaag
pBAD33RBS-FlhDC-sfGFP-mutagenesis-R	ttaactgtgtttcgctttccagcatctgcaaacgag

Appendix Table S2

List of proteins upregulated in S13 Δ*tls* / S13

Protein name	Log_2 fold change	qValue
FlgI	7.351213581	7.21E-06
FlgD	7.325973148	0.00804194
FlgE	6.191881233	3.70E-05
FliH	6.153342889	1.36E-06
LeuA	5.185663192	3.74E-05
FimG	4.848467612	1.93E-05
CheW	4.389860702	5.82E-05
FliA	4.363757937	0.005073002
FliF	4.232305135	0.000237034
LeuD	3.667935925	0.004769032
SpeB	3.448312805	1.93E-05
FlgG	3.431338681	0.000724066
Tar	3.429694008	0.000155975
Tsr	3.308455336	1.93E-05
MotA	3.201898895	0.010401728
CheA	3.131990529	7.00E-05
FliC	2.912247716	5.69E-05
CheB	2.828284057	0.003526445
FlgH	2.743813889	4.55E-05
YcgR	2.539162822	0.004343572
YfjI	2.414365314	0.004124872
LeuC	2.391820982	0.001744058
FimA	2.308037702	0.006404074
YdiY	2.305915691	0.006382149
CheZ	2.167784929	0.00534628
YecN	2.11428877	0.016353859
LeuB	2.074731331	0.000193965
IlvN	1.945668186	5.55E-05
FimC	1.923633951	0.002457092
FlgM	1.865197016	0.000262806
TatE	1.837653714	0.01639495
TyrS	1.792412396	4.55E-05
IlvB	1.591391609	0.038579817
YeiC	1.58055815	0.006370017
FliS	1.429480293	0.025455156
YagU	1.367760628	0.000198773

Ade	1.297893073	0.002591177
IlvC	1.295494168	0.003787796
AroL	1.285305675	0.020241584
YgdL	1.230818852	0.009806369
YeeF	1.200270028	0.000724066
ThrC	1.090937577	0.001868089
Mqo	1.090558652	0.000724066
YcjY	1.083360865	0.0067799
GltD	1.079853165	0.018623219
FolX	1.064222961	0.017219658
FumB	1.057935152	0.031850938
YeaJ	1.038686154	0.00528081

Proteins were selected from the whole-proteome data based on the following cutoffs (qValue < 0.05 and $\log_2$ fold change > 1).

Appendix Table S3

List of proteins downregulated in S13 Δ*tls* / S13

Protein name	Log_2 fold change	qValue
YfdC	-3.260216081	0.00145761
UTI89_C2822	-3.132262334	0.03204625
TdcG	-3.062901464	0.018733151
YbbQ	-2.954570753	0.00064893
EvgA	-2.851109181	0.00678466
TdcE	-2.657183078	7.54E-05
AllB	-2.520192965	0.00416661
PyrI	-2.465295169	0.000178848
IbpA	-2.326631582	0.00027907
Hyi	-2.173388554	0.011009486
YfiL	-2.129046835	0.006590748
YoaE	-2.049354812	0.010401728
RihC	-1.94798737	0.013532265
TdcD	-1.92129096	0.020241584
DppD	-1.905577634	0.000262806
DppF	-1.8556986	0.009651698
YfiB	-1.840360636	0.004343572
UTI89_C0259	-1.804892836	0.009388128
SrlB	-1.781126748	0.010161711
YgiW	-1.617729607	0.001330114
CspA	-1.59865076	0.000600374
AstE	-1.559240593	0.001744058
AldB	-1.523029988	0.00145761
BetA	-1.512700865	0.002174985
YbeX	-1.463823323	0.013532265
NarY	-1.461792434	0.019241197
YtfJ	-1.436538763	0.011009486
YiiP	-1.431388381	0.028431776
UTI89_C0916	-1.339344861	0.020244417
DppA	-1.299234556	0.000714774
GabT	-1.247058324	0.002012305
GltL	-1.145629235	0.009156104
YebE	-1.13736369	0.0023033
ChuA	-1.117269888	0.002174985
PotG	-1.110505356	0.049588612
GabD	-1.104158496	0.005726933

Acs	-1.099197392	0.000682342
IbpB	-1.08363533	0.00145761
YgfY	-1.053319732	0.044163471
YebV	-1.033731549	0.004954361
SthA	-1.020949089	0.000457698
YhdP	-1.019493296	0.021217022

Proteins were selected from the whole-proteome data based on the following cutoffs (qValue < 0.05 and log_2 fold change < -1).

Appendix Table S4

List of proteins upregulated in MG1655 / pTrc99A-Tls

Protein name	Log_2 fold change	qValue
Trg	8.172092067	4.18E-05
HcaR	6.288347351	0.006079974
MdtE	5.788628857	0.030408899
WbbL	5.735248347	4.18E-05
FlhC	5.517327172	0.029358032
YcbK	5.475230089	0.000127274
IntF	5.095088133	0.029971311
YgbJ	4.831216677	0.004494165
YrbL	4.830188071	0.001100159
IbpB	4.723108058	0.000350862
CsiE	4.495601589	0.005471729
YjhQ	3.557635884	0.005174792
IbpA	3.442642193	0.000587077
FlhD	3.220871444	0.000370249
YgbK	3.165375303	0.001056358
TdcD	3.119251804	0.000350862
MalI	2.921981795	0.007825
YmgC	2.833153439	0.018630038
TdcB	2.727638613	0.005760162
RspA	2.684382253	0.004515842
RpoS	2.563019006	0.00083205
Cfa	2.161784523	0.002469804
PuuA	2.084947126	0.026302264
YqeF	2.02224992	0.002990814
SoxR	2.012709915	0.036498516
RecN	1.89235924	0.013697585
YfeR	1.857557507	0.002126001
AraC	1.773101675	0.02635022
MhpR	1.71871277	0.020306782
YmfI	1.669847791	0.018901653
Rph	1.49348264	0.006659936
GatZ	1.461093871	0.027593597
SdiA	1.392015246	0.032440599
AcpH	1.302633643	0.038851505
RcdA	1.260024827	0.048336755
UbiC	1.187351105	0.048336755

TyrA	1.07887148	0.01318711

Proteins were selected from the whole-proteome data based on the following cutoffs (qValue < 0.05 and log_2 fold change > 1).

Appendix Table S5

List of proteins downregulated in MG1655 / pTrc99A-Tls

Protein name	Log_2 fold change	qValue
MotB	-6.094057644	0.010504373
FlgG	-5.603715103	0.000350862
FliH	-4.789164624	0.000874508
FlxA	-4.378537318	0.000549636
YoaC	-3.918496466	0.001584718
FliD	-3.759584332	0.000587077
FlgL	-3.563929136	0.031355695
FlgK	-3.381687063	0.002140481
CheR	-3.186058312	0.012349592
FlgI	-3.130749225	0.000564691
HisD	-2.758650175	0.009654017
Tap	-2.711783484	0.006106917
FlgE	-2.587659333	0.001388537
IraP	-2.490379841	0.02525086
FliC	-2.353650724	0.004644067
CheB	-2.255084224	0.000874508
YnjH	-2.176635041	0.004494165
FlgN	-2.090665141	0.004125978
CheA	-2.043841663	0.00125362
FliL	-1.955390261	0.002246353
ArsR	-1.93662037	0.011488597
CheZ	-1.902844657	0.011488597
CheW	-1.800666202	0.003843912
Tar	-1.795929201	0.00150226
Tsr	-1.740740116	0.001559471
FliF	-1.701977249	0.002140481
DinI	-1.64161537	0.018672372
FlgH	-1.589908134	0.010924265
CheY	-1.587618178	0.016427406
FlhE	-1.574613776	0.016136912
QueF	-1.533805901	0.024000435
YghA	-1.502905546	0.010504373
FlgM	-1.392350169	0.006106917
NapA	-1.371914687	0.010865992
FliN	-1.331591541	0.016136912
CysI	-1.283692293	0.006659936

GsiB	-1.262329106	0.032440599
FliA	-1.238019765	0.009831583
YjiM	-1.213785035	0.02635022
FliM	-1.208800257	0.046922867
MalE	-1.193179164	0.024000435
CsiD	-1.174264682	0.012517642
NemA	-1.152588682	0.021590183
IlvN	-1.136453091	0.029358032
CysJ	-1.135023678	0.010504373

Proteins were selected from the whole-proteome data based on the following cutoffs (qValue < 0.05 and log_2 fold change < -1).

Appendix Table S6

List of proteins upregulated in MG1655 Δ*flhC* / pTrc99A-Tls

Protein name	Log_2 fold change	qValue
Trg	11.05457949	0.0025092
IbpB	4.47547236	0.001128238
AsnC	3.492133855	0.002097973
IbpA	2.40902847	0.001128238
TdcB	1.601100624	0.013100334
TdcD	1.044427768	0.002097973
YehU	1.00621539	0.0158579

Proteins were selected from the whole-proteome data based on the following cutoffs (qValue < 0.05 and log_2 fold change > 1).

Appendix Table S7

List of proteins downregulated in MG1655 Δ*flhC* / pTrc99A-Tls

Protein name	Log_2 fold change	qValue
FrwB	-2.518148776	0.000211347
LsrG	-1.285153098	0.000942771
CreB	-1.0722013	0.009755836

Proteins were selected from the whole-proteome data based on the following cutoffs (qValue < 0.05 and log_2 fold change < -1).

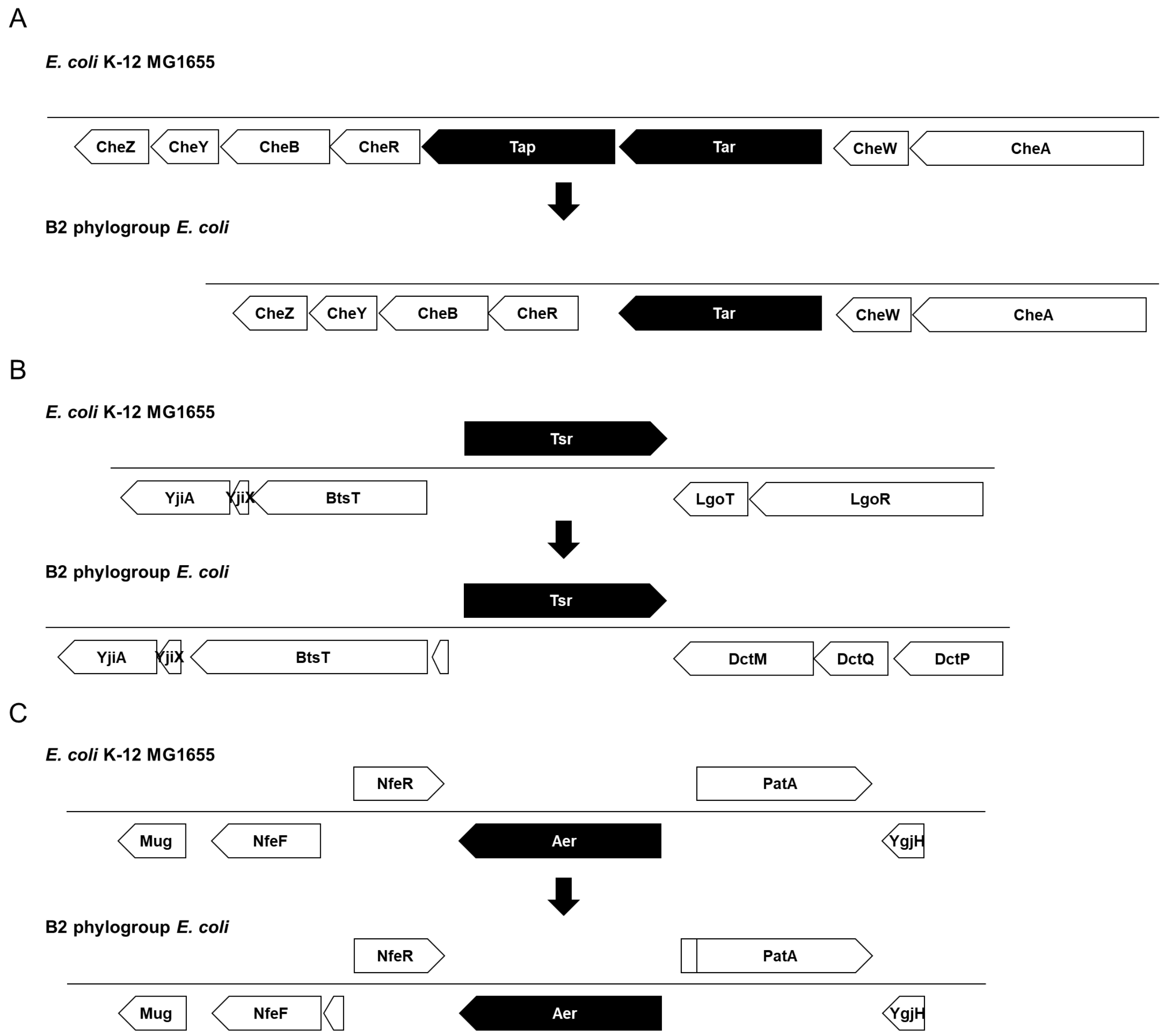

Appendix Figure S1. Genomic contexts of chemoreceptors in B2 phylogroup *E. coli*. (A-C) Schematic representation for the genomic structure of *tar* and *tap* (A), *tsr* (B), and *aer* (C) genes in *E. coli* UTI89, which is representative for B2 strains, based on the NCBI and Microbial Signal Transduction Database 4.0.

```
            Trg (MG1655)  MNTTPSQRLGFLHHIRLVPLFACILGGILVLFALSSALAGYFLWQADRDQRDVTAEIEIR  60
                Tls (S13)  ------------------------------------------------------------  0
              Tls (UTI89)  ------------------------------------------------------------  0
  Tls variant (E2348/69)  ------------------------------------------------------------  0

            Trg (MG1655)  TGLANSSDFLRSARINMIQAGAASRIAEMEAMKRNIAQAESEIKQSQQGYRAYQNRPVKT  120
                Tls (S13)  ------------------------------------------------------------  0
              Tls (UTI89)  ------------------------------------------------------------  0
  Tls variant (E2348/69)  ------------------------------------------------------------  0

            Trg (MG1655)  PADEALDTELNQRFQAYITGMQPMLKYAKNGMFEAIINHESEQIRPLDNAYTDILNKAVK  180
                Tls (S13)  ------------------------------------------------------------  0
              Tls (UTI89)  ------------------------------------------------------------  0
  Tls variant (E2348/69)  ------------------------------------------------------------  0

            Trg (MG1655)  IRSTRANQLAELAHQRTRLGGMFMIGAFVLALVMTLITFMVLRRIVIRPLQHAAQRIEKI  240
                Tls (S13)  ------------------------------------------------------------  0
              Tls (UTI89)  ------------------------------------------------------------  0
  Tls variant (E2348/69)  ------------------------------------------------------------  0

            Trg (MG1655)  ASGDLTMNDEPAGRNEIGRLSRHLQQMQHSLGMTVGTVRQGAEEIYRGTSEISAGNADLS  300
                Tls (S13)  ------------------------------------------------------------  0
              Tls (UTI89)  ------------------------------------------------------------  0
  Tls variant (E2348/69)  ------------------------------------------------------------  0

            Trg (MG1655)  SRTEEQAAAIEQTAASMEQLTATVKQNADNAHHASKLAQEASIKASDGGQTVSGVVKTMG  360
                Tls (S13)  ------------------------------------------------------------  0
              Tls (UTI89)  ------------------------------------------------------------  0
  Tls variant (E2348/69)  ------------------------------------------------------------  0

            Trg (MG1655)  AISTSSKKISEITAVINSIAFQTNILALNAAVEAARAGEQGRGFAVVASEVRTLASRSAQ  420
                Tls (S13)  ------------------------------MNVRIVLLSQGPGFAVVASEVRTLASRSAQ  30
              Tls (UTI89)  ------------------------------MNVRIVLLSQGPGFAVVASEVRTLASRSAQ  30
  Tls variant (E2348/69)  ---------------------------MRFSQFNHSLLSQGRGFAVVASEVRTLASRSAQ  33
                                                                .** ******************
            Trg (MG1655)  AAKEIEGLISESVRLIDLGSDEVATAGKTMSTIVDAVASVTHIMQEIAAASDEQSRGITQ  480
                Tls (S13)  AAKEIEGLISESVRLIDLGSDEVATAGKIMCTIVDAVASVTHIMQEIATASGEQSRGITQ  90
              Tls (UTI89)  AAKEIEGLISESVRLIDLGSDEVATAGKIMCTIVDAVASVTHIMQEIATASGEQSRGITQ  90
  Tls variant (E2348/69)  TAKEIEGLISESVRLIDLGSDEVATAGKTMCTIVDAVASVTHIMQEIATASDEQSRGITQ  93
                          :*************************** *.*****************:**.********
            Trg (MG1655)  VSQAISEMDKVTQQNASLVEEASAAAVSLEEQAARLTEAVDVFRLHKHSVSAEPRGAGEP  540
                Tls (S13)  VSQAISEMDKVTQQNASLVEEASTAAVSLEEQAARLTEAVDVFRLNKHSVSAEPRGACEP  150
              Tls (UTI89)  VSQAISEMDKVTQQNASLVEEASTAAVSLEEQAARLTEAVDVFRLNKHSVSAEPRGACEP  150
  Tls variant (E2348/69)  VSQAISEMDKVTQQNASLVEEASTAAVSLEEQAARLTEAVDVFRLNKHSVSAEPRGACEP  153
                          ***********************:*********************:*********** **
            Trg (MG1655)  VSFATV  546
                Tls (S13)  VSFATV  156
              Tls (UTI89)  VSFATV  156
  Tls variant (E2348/69)  VSFATV  159
                          ******
```

Appendix Figure S2. Amino acid sequence alignment between Trg and Tls. Alignment of Tls sequences from *E. coli* B2 strains S13, UTI89, and E2348/69 with that of Trg from *E. coli* MG1655 using Clustal Omega software. Identical (*), strongly similar (:), and weakly similar (.) residues are indicated. The short N-terminal amino acid sequence MNVRIVLLS, and its variant sequence MRFSQFNHSLLS are marked in red and blue, respectively.

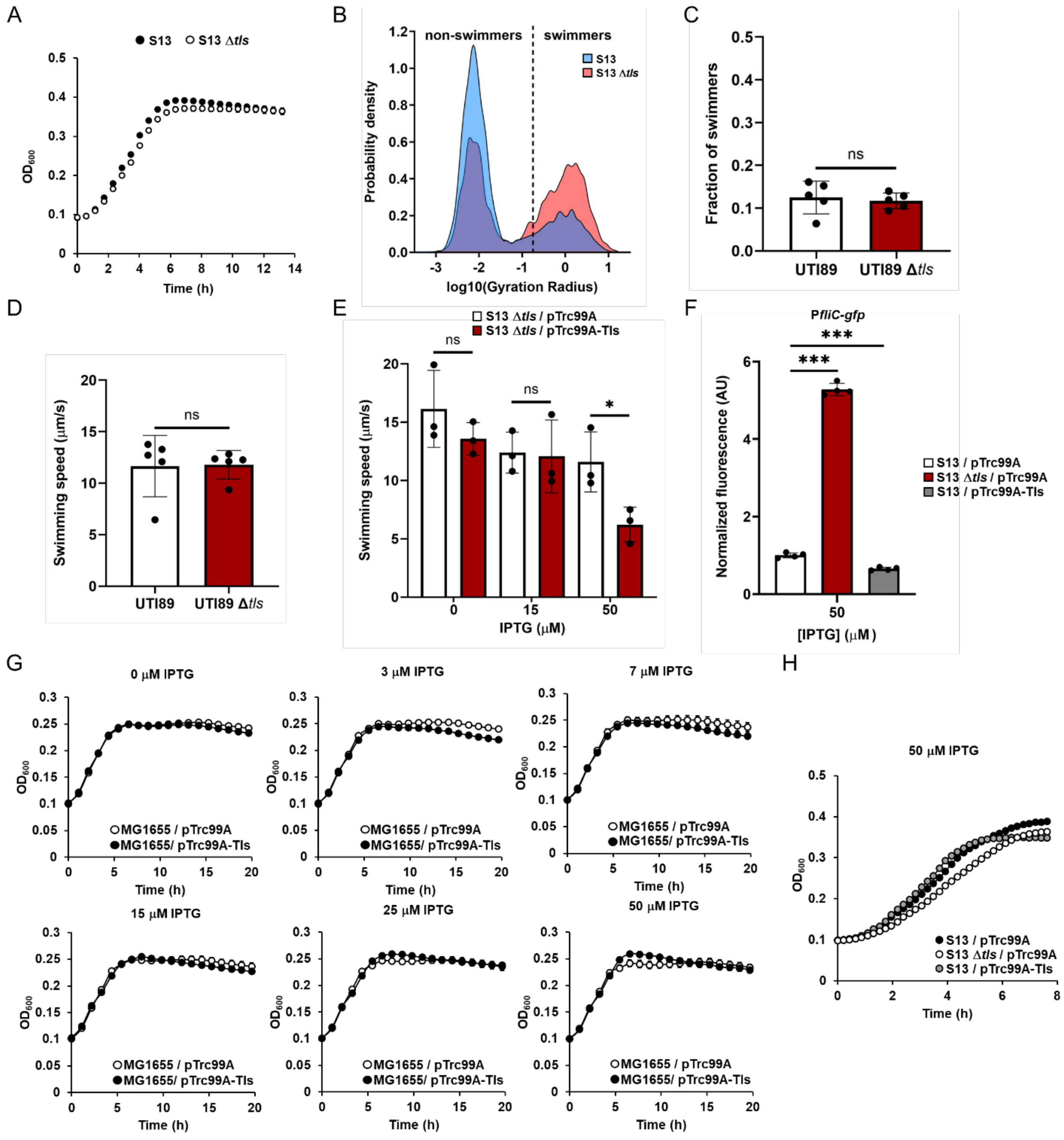

Appendix Figure S3. Effect of Tls on motility, flagellar gene expression, and growth. (A) Growth curves of *E. coli* strain S13 and its Δ*tls* mutant in TB medium as a function of time, measured in a plate reader. Values represent the means and standard deviations of a minimum of three biological replicates. (B) Distribution of gyration radius (proxy for diffusion coefficient) obtained from analysis of motility of *E. coli* S13 and its Δ*tls* mutant cells (see Methods), with vertical dotted line indicating a threshold for separating swimmers from non-swimmers. (C-D) Fraction of swimmers (C) and swimming speed (D) of *E. coli* UTI89 and its

Δ*tls* mutant cells. Values represent the means and standard deviations of five biological replicates. The analysis is performed as described in the Methods. Statistical significance was determined using unpaired two-tailed Student's *t*-test. The P values are denoted as ns ($P > 0.05$), * ($P < 0.05$), ** ($P < 0.005$), *** ($P < 0.001$). (ns = 0.70 (panel C); ns = 0.93 (panel D)). (E) Swimming speed for *E. coli* S13 Δ*tls* mutant cells carrying either empty vector pTrc99A or pTrc99A-Tls induced by the indicated concentrations of IPTG. Values represent the means and standard deviations of three biological replicates. Statistical significance was determined using unpaired two-tailed Student's *t*-test. The P values are denoted as ns ($P > 0.05$), * ($P < 0.05$), ** ($P < 0.005$), *** ($P < 0.001$). (P values from left to right: ns = 0.28, ns = 0.88, *$P = 0.0352$). (F) Fluorescence levels of P*fliC-gfp* normalized by OD_{600} in *E. coli* strain S13 and its Δ*tls* mutant, both carrying an empty vector pTrc99A, and in S13 expressing Tls from pTrc99A-Tls plasmid induced by 50 μM IPTG. Measurements were performed in the log phase of growth in a plate reader. Values represent the means and standard deviations of a minimum of three biological replicates, in each case normalized to the reporter activity in the reference strain (S13 / pTrc99A). Statistical significance was determined using unpaired two-tailed Student's *t*-test (P values from left to right: ***$P = 4.34E-09$, ***$P = 0.0001$). (G-H) Growth curves in TB medium as a function of time for MG1655 carrying either empty vector pTrc99A or pTrc99A-Tls (G), or of *E. coli* strain S13 and its Δ*tls* mutant either carrying an empty vector pTrc99A or pTrc99A-Tls expression plasmid (H), induced by indicated concentrations of IPTG, measured in a plate reader. Values represent the means and standard deviations of a minimum of three biological replicates.

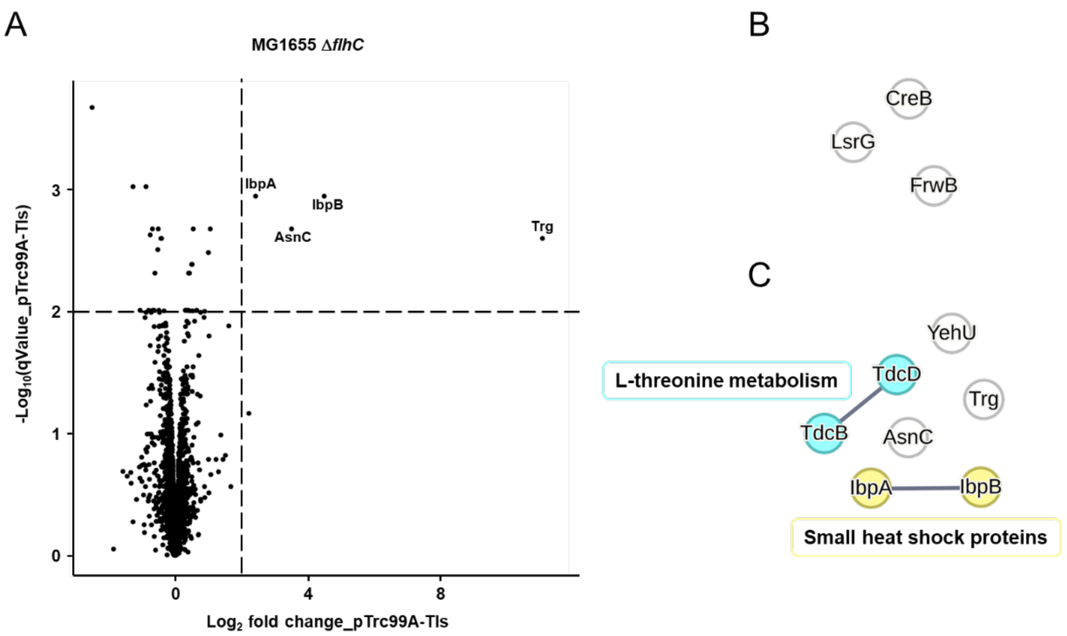

Appendix Figure S4. Whole proteome analysis of regulation by Tls in Δ*flhC* background.

(A) Volcano plot showing changes in protein levels upon expression of Tls in MG1655 Δ*flhC* from pTrc99A-Tls at 15 µM IPTG induction. This result is from three independent experiments. Only proteins significantly altered in their abundance are highlighted. (B-C) The STRING diagram of the clustering of downregulated (B) or upregulated (C) proteins. For the upregulated proteins, the clusters of L-threonine metabolism, and small heat shock proteins are highlighted in cyan, and yellow, respectively. The thickness of the lines indicates the strength of data support in STRING, and all proteins identified are listed in Appendix Table S6 and S7.

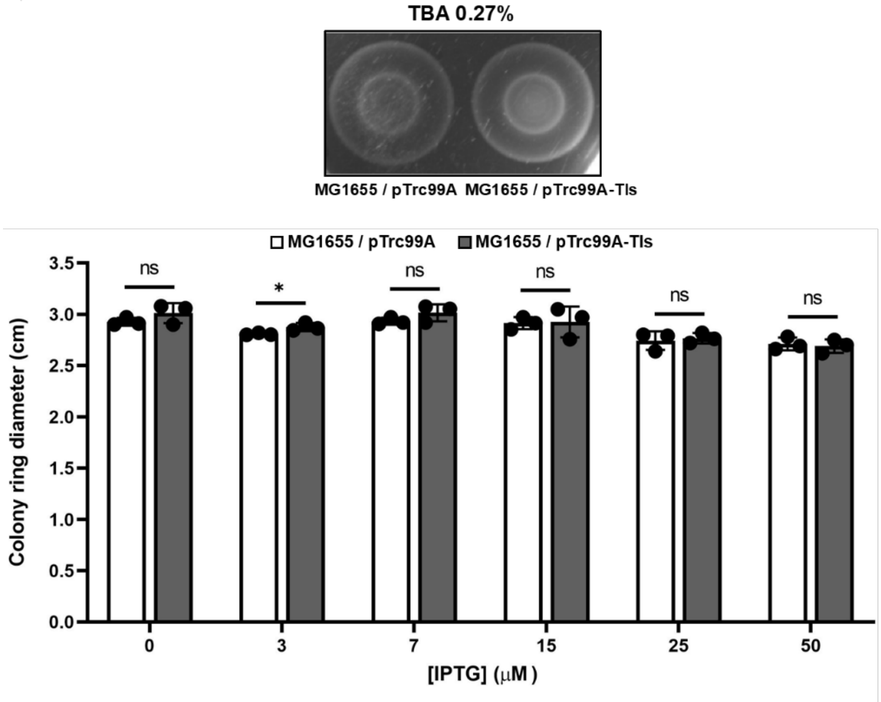

Appendix Figure S5. The effect of Tls on the motility on semi-solid medium. The MG1655 carrying either empty vector pTrc99A or pTrc99A-Tls induced by indicated concentrations of IPTG were spotted on the surface of 0.27% TB agar (TBA) plate. Representative image is shown from three biological replicates. Quantified colony ring diameter was shown below. Values represent the means and standard deviations of a minimum of three biological replicates. Statistical significance was determined using unpaired two-tailed Student's *t* test. The *P* values are denoted as ns ($P > 0.05$), * ($P < 0.05$), ** ($P < 0.005$), *** ($P < 0.001$). (*P* values from left to right: ns = 0.2301, *$P = 0.048$, ns = 0.18, ns = 0.90, ns = 0.71, ns = 0.71).

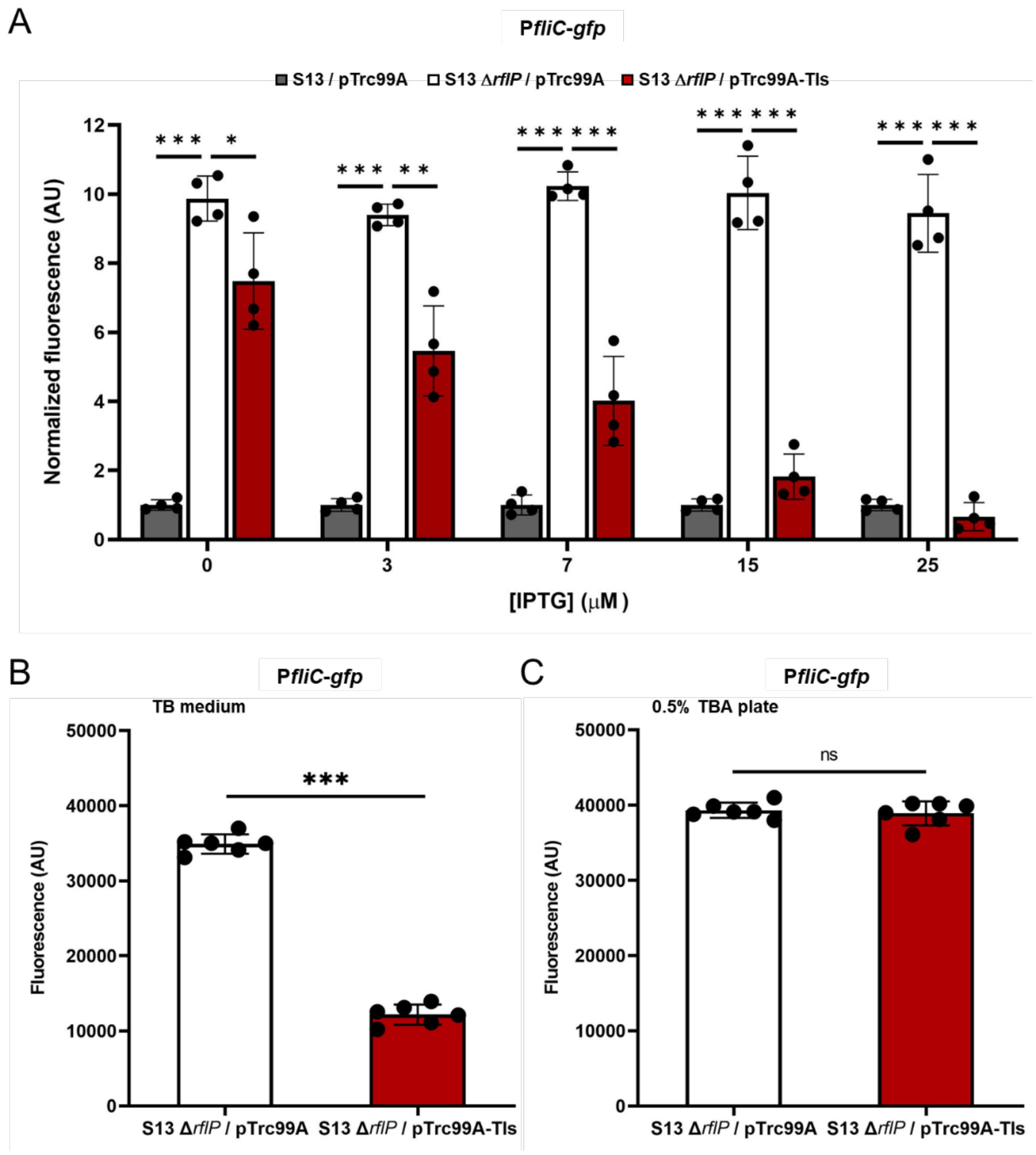

Appendix Figure S6. Downregulation of flagellar gene expression by Tls in RflP-independent manner. (A) Fluorescence levels of P*fliC-gfp* normalized by OD_{600} in *E. coli* strain S13 and its Δ*rflP* mutant, both carrying an empty vector pTrc99A, and in Δ*rflP* mutant expressing Tls from pTrc99A-Tls plasmid induced by indicated concentrations of IPTG. Measurements were performed in the log phase of growth in a plate reader. Values represent the means and standard deviations of a minimum of three biological replicates, in each case normalized to the reporter activity in the reference strain (S13 / pTrc99A). (B-C) Fluorescence levels of P*fliC-gfp* at log phase normalized by OD_{600} in *E. coli* S13 Δ*rflP* cells carrying either empty pTrc99A vector or pTrc99A-Tls expression plasmid induced with 15 μM IPTG, grown

in liquid TB medium (B) or on the surface of 0.5% TB agar (TBA) plate (C). Values represent the means and standard deviations of a minimum of three biological replicates. Statistical significance was determined using unpaired two-tailed Student's *t* test. The P values are denoted as ns ($P > 0.05$), * ($P < 0.05$), ** ($P < 0.005$), *** ($P < 0.001$). (P values from left to right: ***P = 1.94E-07, *P = 0.0211, ***P = 6.84E-09, **P = 0.001, ***P = 2.78E-08, ***P = 9.32E-05, ***P = 2.87E-06, ***P = 1.20E-05, ***P = 5.87E-06, ***P = 6.30E-06 (panel A); ***P = 4.20E-11 (panel B); ns = 0.62 (panel C)).

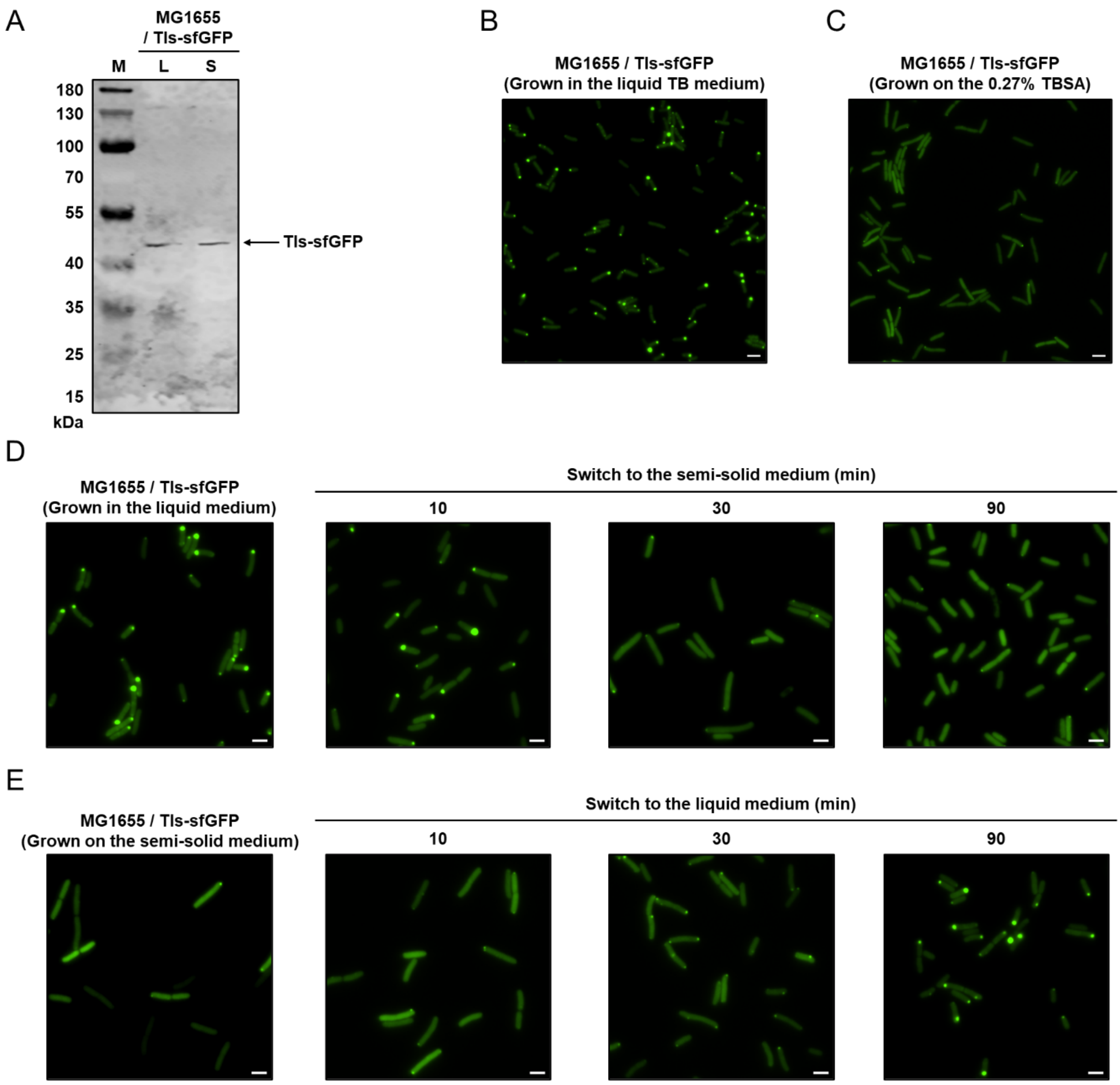

Appendix Figure S7. Surface-sensitive subcellular localization of Tls. (A) The amount of Tls-sfGFP fusion protein in MG1655 cells carrying pTrc99A-Tls expression plasmid induced with 15 μM IPTG, monitored via immunoblotting with anti-GFP primary antibody, in cells grown in the liquid TB medium (L) or on 0.5% TBA plate (S). Equal amounts of total protein were loaded for both samples. The immunoblot is a representative of two biological replicates. (B-C) Cellular localization of Tls-sfGFP in MG1655 cells carrying Tls-sfGFP expression plasmid induced with 15 μM IPTG, grown either in the liquid TB medium (B) or on 0.27% TBA plate (C). Representative images are shown from three biological replicates. Scale bars, 3 μm. (D-E) Changes in cellular localization of Tls-sfGFP over time upon transferring MG1655

cells carrying pTrc99A-Tls-sfGFP from growth in liquid TB medium to growth on 0.5% TBA plate (D), or vice versa (E). Representative images are shown from three biological replicates. Scale bars, 2 μm.

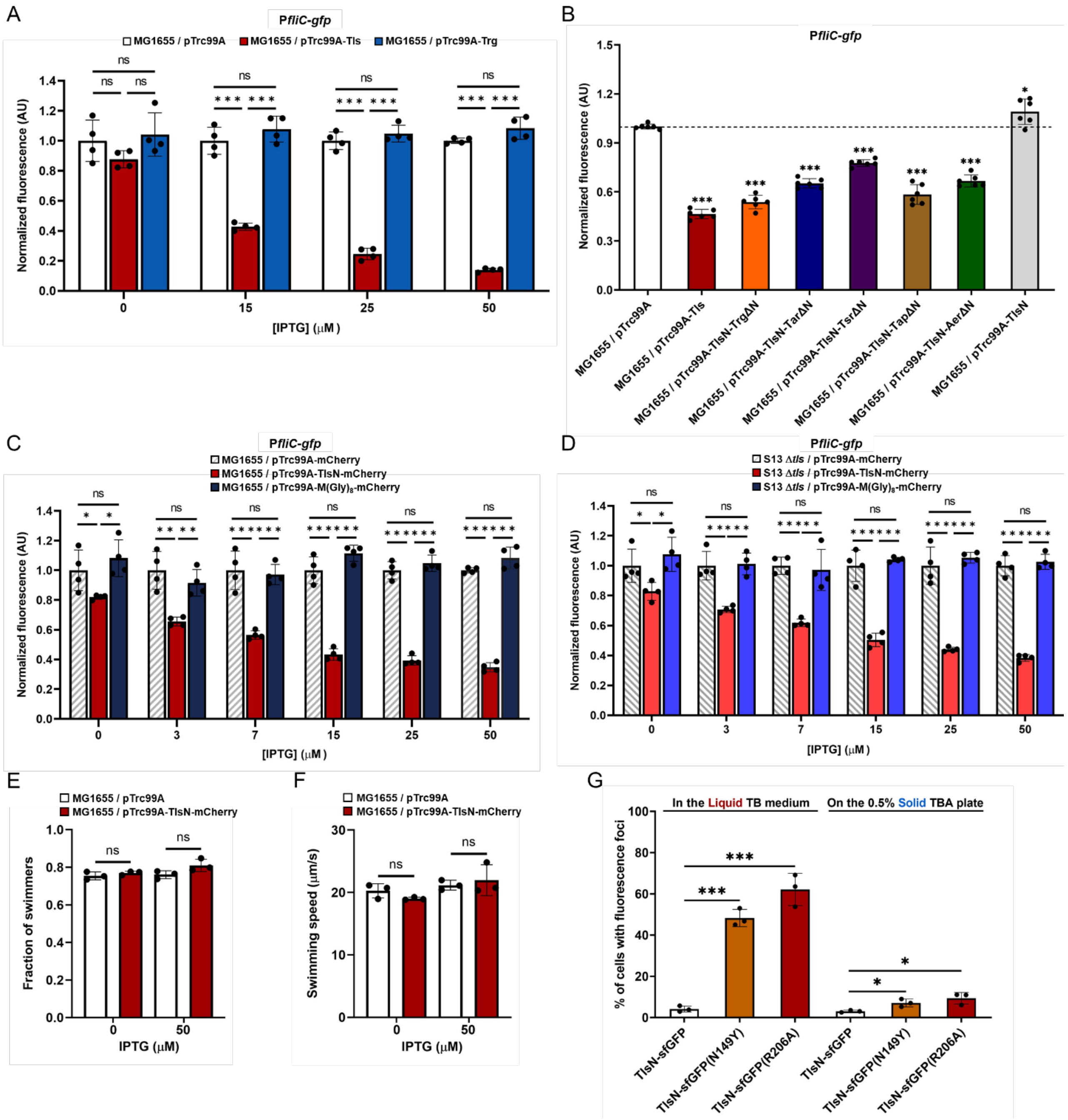

Appendix Figure S8. Importance of the N-terminal sequence of Tls for its functionality. (A) Fluorescence levels of P*fliC-gfp* normalized by OD_{600} in MG1655 carrying either empty vector pTrc99A or pTrc99A-Tls, or pTrc99A-Trg at indicated levels of IPTG induction. (B) Fluorescence levels of P*fliC-gfp* normalized by OD_{600} in MG1655 expressing Tls, the N-terminal Tls sequence (TlsN) alone, or indicated fusion constructs between TlsN and cytoplasmic fragments of *E. coli* chemoreceptors expressed from pTrc99A vector with 15 µM IPTG induction. TrgΔN, amino acids 400-546 of Trg; TarΔN, amino acids 390-553 of Tar;

TsrΔN, amino acids 392-551 of Tsr; TapΔN, amino acids 388-533 of Tap; AerΔN, amino acids 382-506 of Aer. Cells were grown in liquid TB in a plate reader. Values represent the means and standard deviations of a minimum of three biological replicates, in each case normalized to the reporter activity in the reference strain (MG1655 / pTrc99A). (C-D) Fluorescence levels of P*fliC-gfp* normalized by OD_{600} in MG1655 (C) or S13 Δ*tls* mutant (D) expressing either TlsN or a control $M(Gly)_8$ sequence fused to mCherry (which does not interfere with fluorescence measurements of the GFP reporter activity) from pTrc99A at indicated levels of IPTG induction. Measurements were performed in the log phase of growth in a plate reader. Values represent the means and standard deviations of a minimum of three biological replicates, in each case normalized to the reporter activity in the respective reference strain (MG1655 / pTrc99A-mCherry or S13 Δ*tls* / pTrc99A-mCherry). (E-F) Fraction of swimmers (E) and swimming speed (F) of MG1655 carrying either empty vector pTrc99A or pTrc99A-TlsN-mCherry induced by 50 μM of IPTG. Values represent the means and standard deviations of three biological replicates. (G) Quantification of the number of cells showing fluorescence foci formed in microscopy images as in Fig. 5C. For each construct, foci were counted in 120 cells from 3 different images, originating from three biological replicates. Values represent the means and standard deviations. Statistical significance was determined using unpaired two-tailed Student's *t* test. The *P* values are denoted as ns ($P > 0.05$), * ($P < 0.05$), ** ($P < 0.005$), *** ($P < 0.001$). (*P* values from left to right: ns = 0.15, ns = 0.69, ns = 0.076, ****P* = 1.77E-05, ns = 0.26, ****P* = 6.45E-06, ****P* =6.60E-07, ns = 0.28, ****P* = 3.74E-07, ****P* = 3.07E-10, ns = 0.072, ****P* = 2.59E-07 (panel A); ****P* = 2.08E-12, ****P* = 1.76E-10, ****P* = 1.41E-10, ****P* = 1.20E-09, ****P* = 1.60E-08, ****P* = 1.71E-09, **P* = 0.019 (panel B); **P* = 0.04, ns = 0.41, **P* = 0.0057, ***P* = 0.0018, ns = 0.32, ***P* = 0.0014, ****P* = 0.0005, ns = 0.71, ****P* = 3.36E-05, ****P* = 2.52E-05, ns = 0.072, ****P* = 9.10E-07, ****P* = 1.81E-06, ns = 0.28, ****P* = 9.26E-07, ****P* = 2.87E-08, ns = 0.072, ****P* = 1.69E-06 (panel C); **P* = 0.033, ns = 0.38,

*P = 0.0082, **P = 0.0011, ns = 0.82, ***P = 0.0002, ***P = 1.17E-05, ns = 0.7, **P = 0.0024, ***P = 0.0001, ns = 0.46, ***P = 7.44E-07, ***P = 0.0001, ns = 0.43, ***P = 6.33E-08, ***P = 2.48E-06, ns = 0.52, ***P = 4.03E-07 (panel D); ns = 0.31, ns = 0.092 (panel E); ns = 0.14, ns = 0.62 (panel F); ***P = 6.74E-05, ***P = 0.0002, *P = 0.023, *P = 0.019 (panel G)).

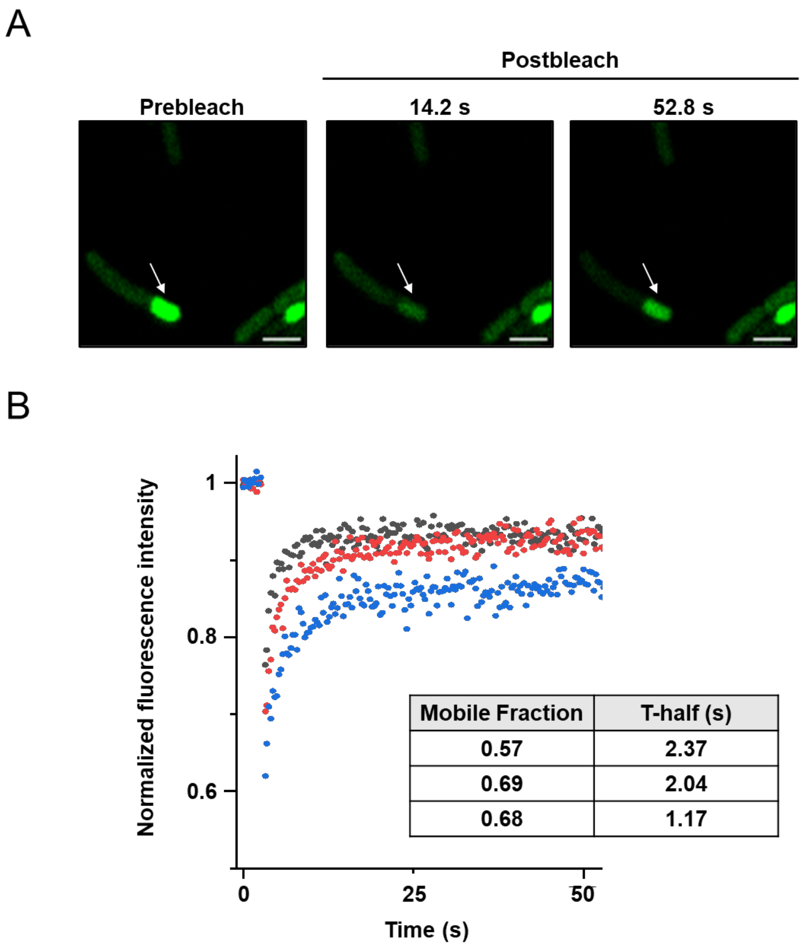

Appendix Figure S9. Formation of dynamic structure near the cell pole by Tls. (A-B) FRAP assay of focus formed by Tls-sfGFP expressed from pTrc99A vector with 15 µM IPTG induction in MG1655. (A) Whole region of focus (white arrow) was bleached as shown in the representative image from at least three biological replicates. Scale bars, 2 µm. (B) Fluorescence intensities from 3 different cells were quantified as a function of time, normalized both by the value of prebleach and the integral fluorescence of the entire cell, and the values for mobile fraction and half time of recovery were calculated for each individual cell.

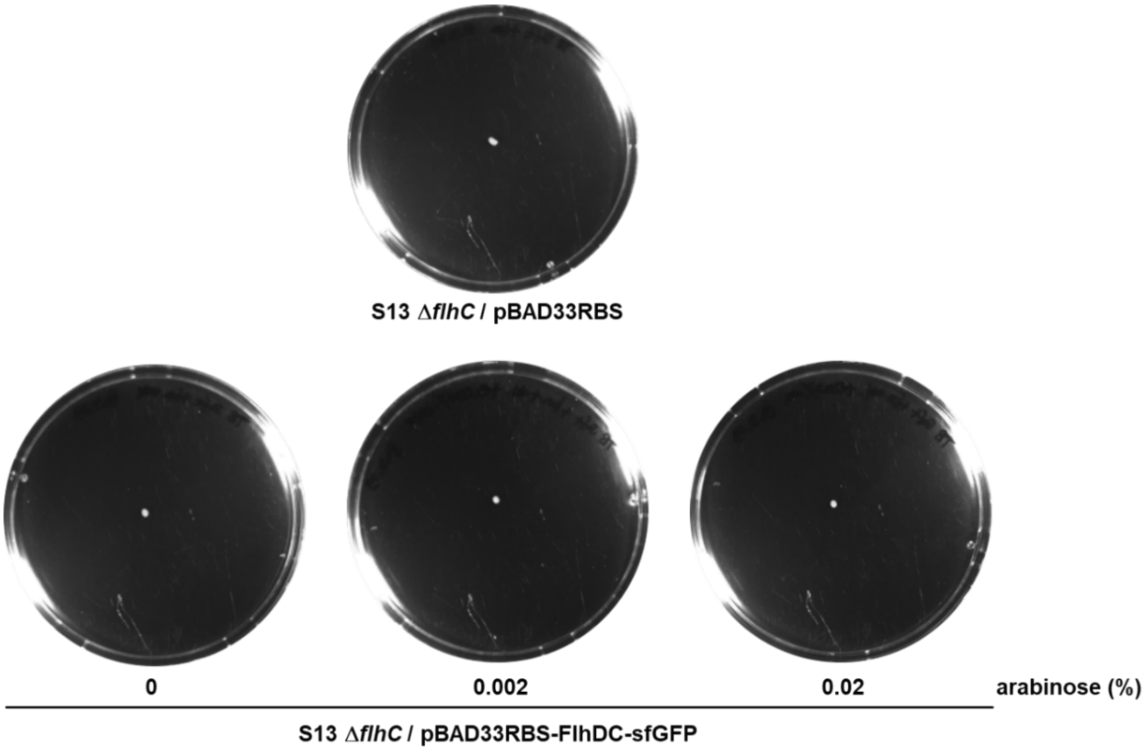

Appendix Figure S10. Functionality test of FlhC-sfGFP in Δ*flhC* strain. The S13 Δ*flhC* carrying either empty vector pBAD33RBS or pBAD33RBS-FlhDC-sfGFP induced by indicated concentrations of arabinose were spotted on the surface of 0.27% TB agar (TBA) plate. Representative images are shown from three biological replicates. Lack of spreading suggests the non-functionality of FlhC-sfGFP fusion.

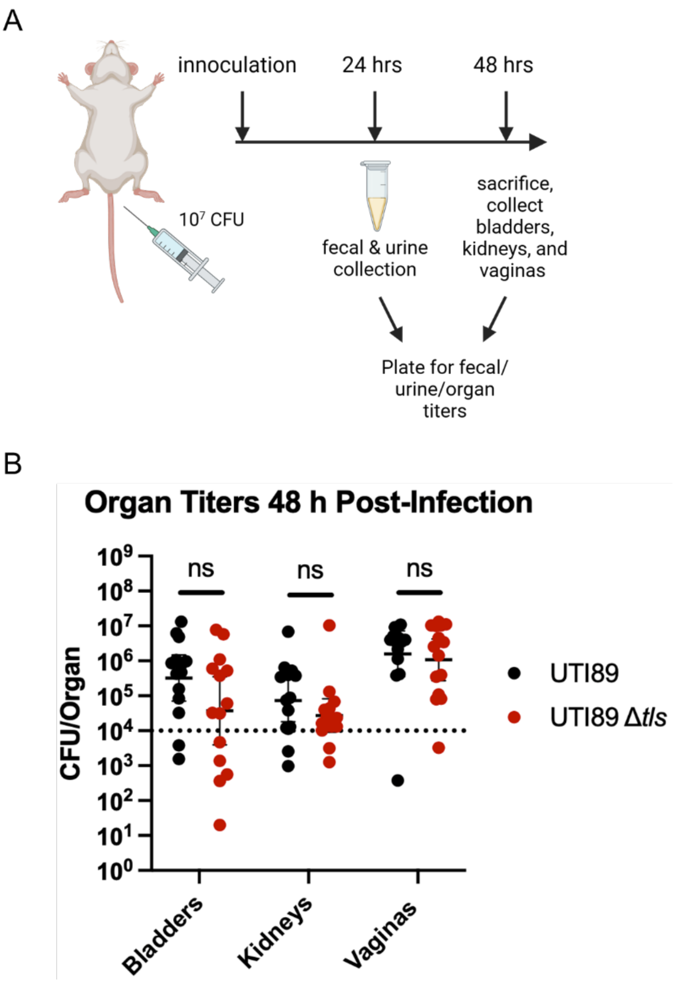

Appendix Figure S11. Testing role of Tls in urinary tract infection. (A) The scheme of colonization experiment. Mice were inoculated with 10^7 c.f.u. UTI89 WT or the isogenic Δ*tls* mutant. Organs were collected at designated time and quantified for bacterial titers. (B) Graph depicts bacterial titers recovered from organs of mice infected with either wild-type UTI89 (black circles) or the isogenic Δ*tls* mutant (red circles). Each dot represents an individual mouse. Mice were each transurethrally inoculated with 50 µl of inoculum containing 10^7 CFU. Mice were sacrificed at 48 h post inoculation and organs were harvested, homogenized and evaluated for colonization. Analyses were performed using 14-15 mice and analyzed using unpaired two-tailed Mann Whitney U test. The P values are denoted as ns ($P > 0.05$), * ($P < 0.05$), ** ($P < 0.005$), *** ($P < 0.001$). (P values from left to right: ns = 0.1280, ns = 0.1465, ns = 0.6591). Dashed line indicates the clinical threshold for UTI in humans. The line depicts the geometric mean +/- 95% CI.
