# Supplementary figures and images for: Control of flagellar gene expression by a chemotaxis receptor-like regulator in pathogenic Escherichia coli
Source: EMBO J. 2025 Oct 13;44(22):6675–703. doi: 10.1038/s44318-025-00595-x (PMC12623472; doi:10.1038/s44318-025-00595-x)

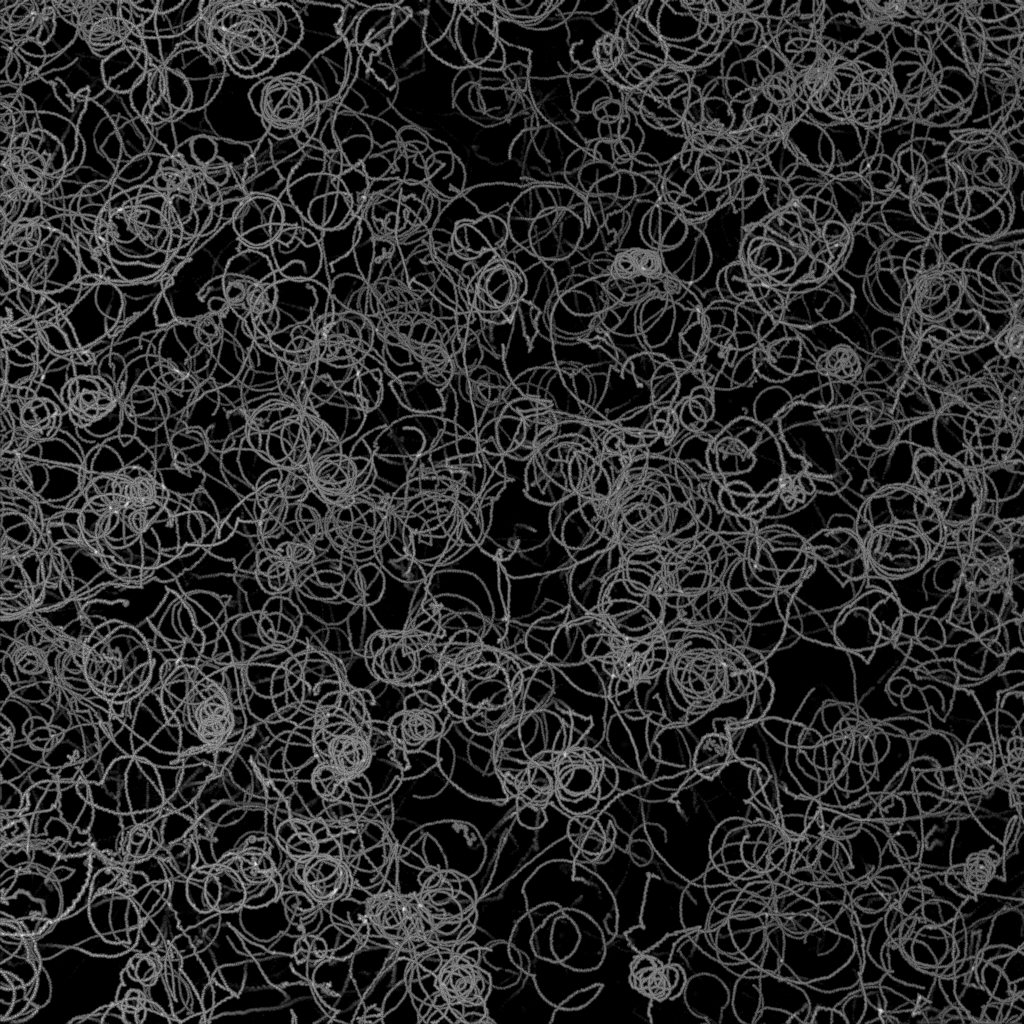

Supplement: Supplementary file 4 — Source data Fig. 2 [file 44318_2025_595_MOESM4_ESM.zip › Fig. 2/2B/2B_S13 tls mutant.tif]

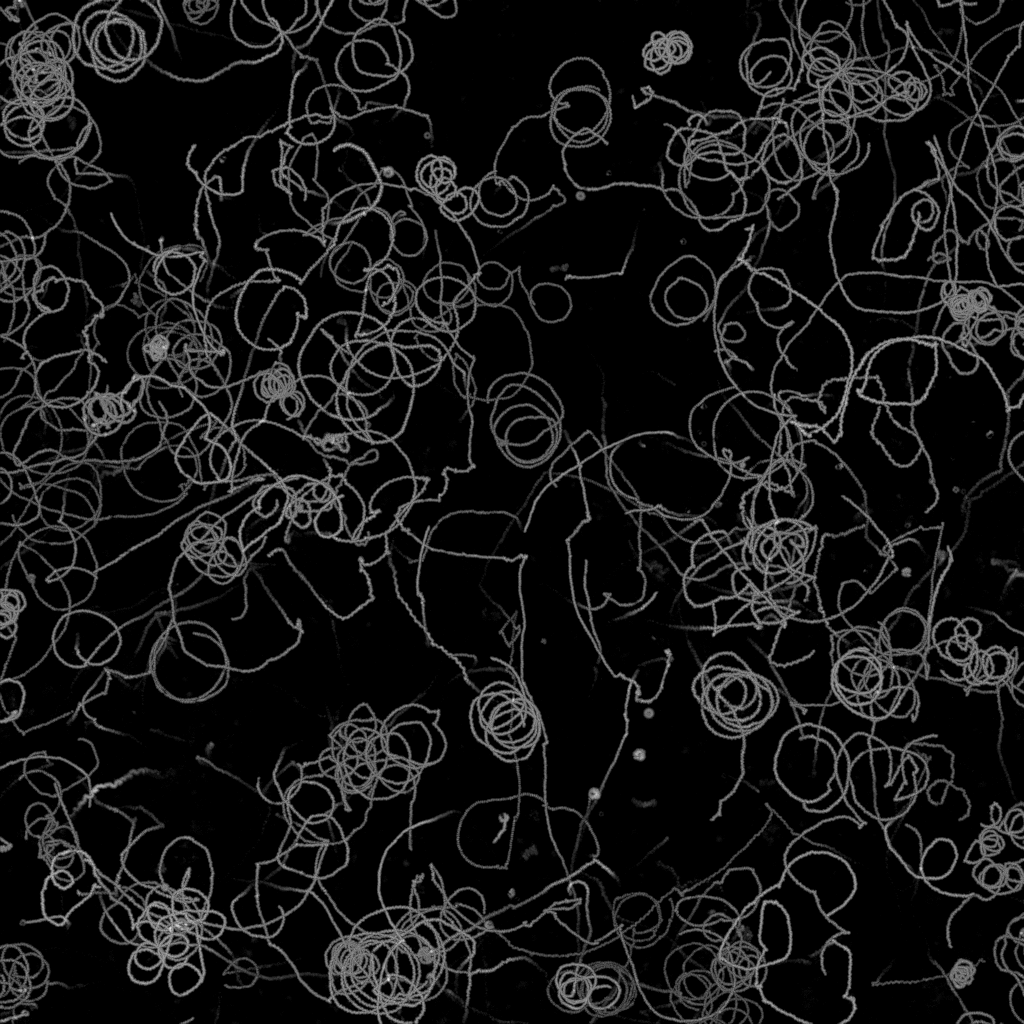

Supplement: Supplementary file 4 — Source data Fig. 2 [file 44318_2025_595_MOESM4_ESM.zip › Fig. 2/2B/2B_S13 wild type.tif]

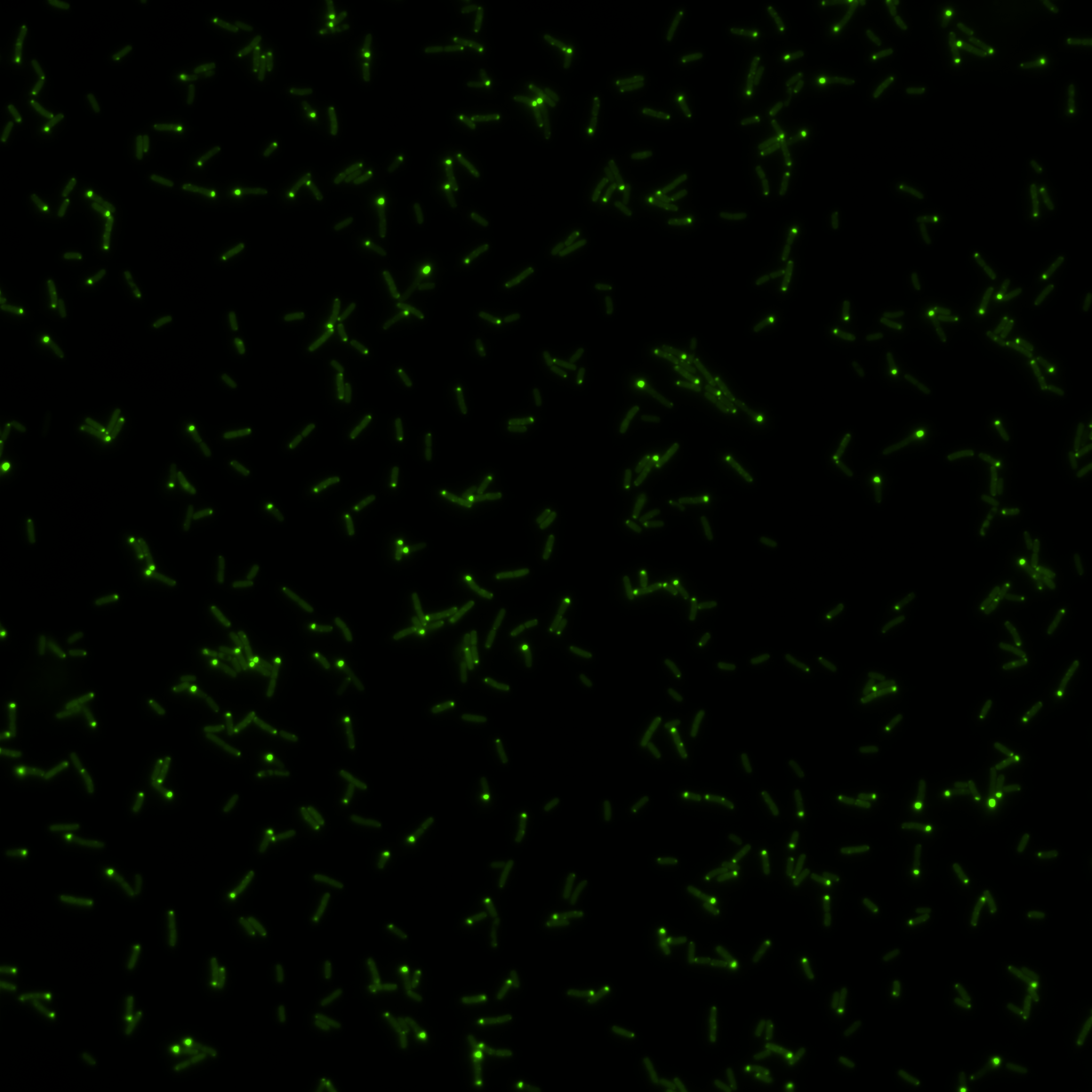

Supplement: Supplementary file 6 — Source data Fig. 4 [file 44318_2025_595_MOESM6_ESM.zip › Fig. 4/4E/4E.tif]

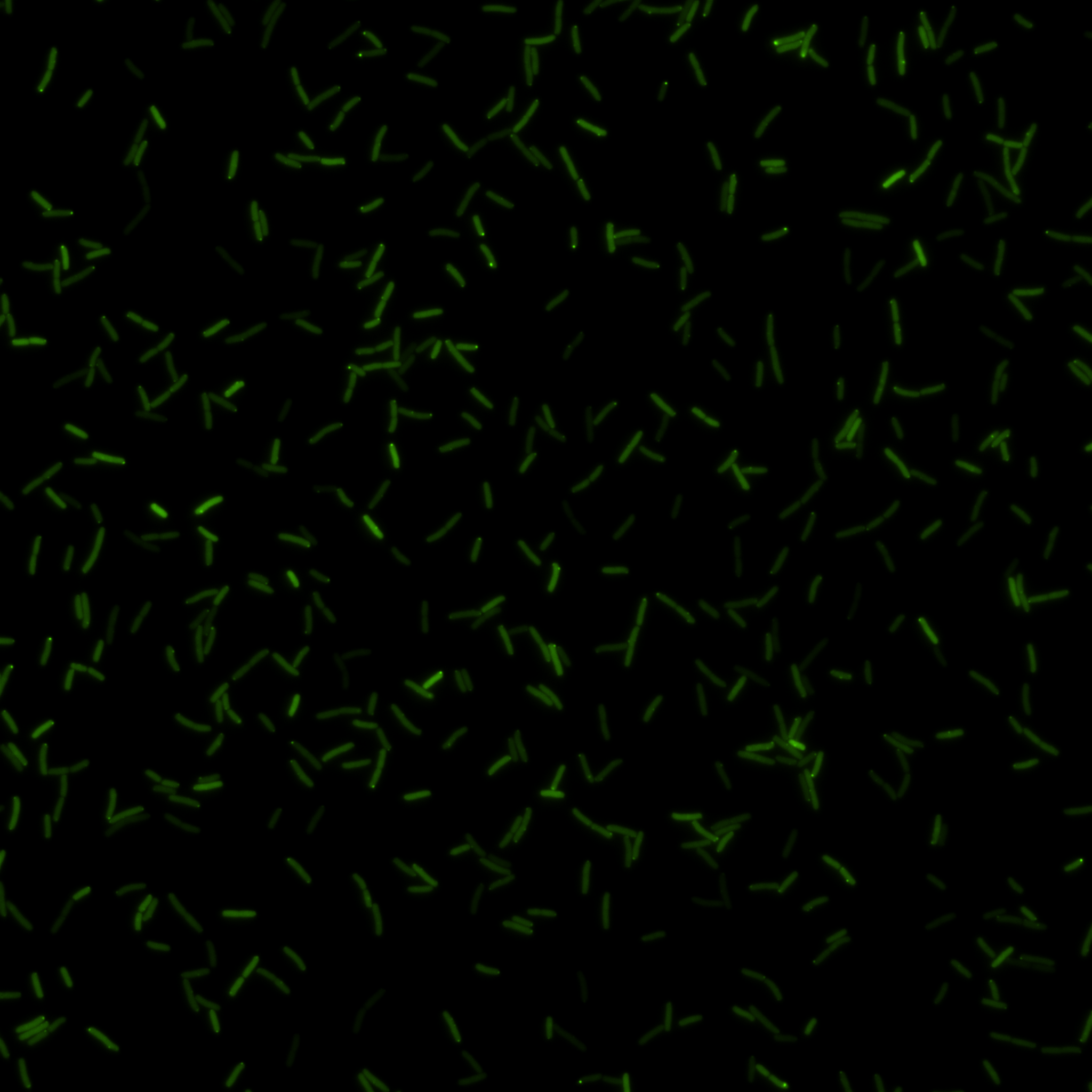

Supplement: Supplementary file 6 — Source data Fig. 4 [file 44318_2025_595_MOESM6_ESM.zip › Fig. 4/4F/4F.tif]

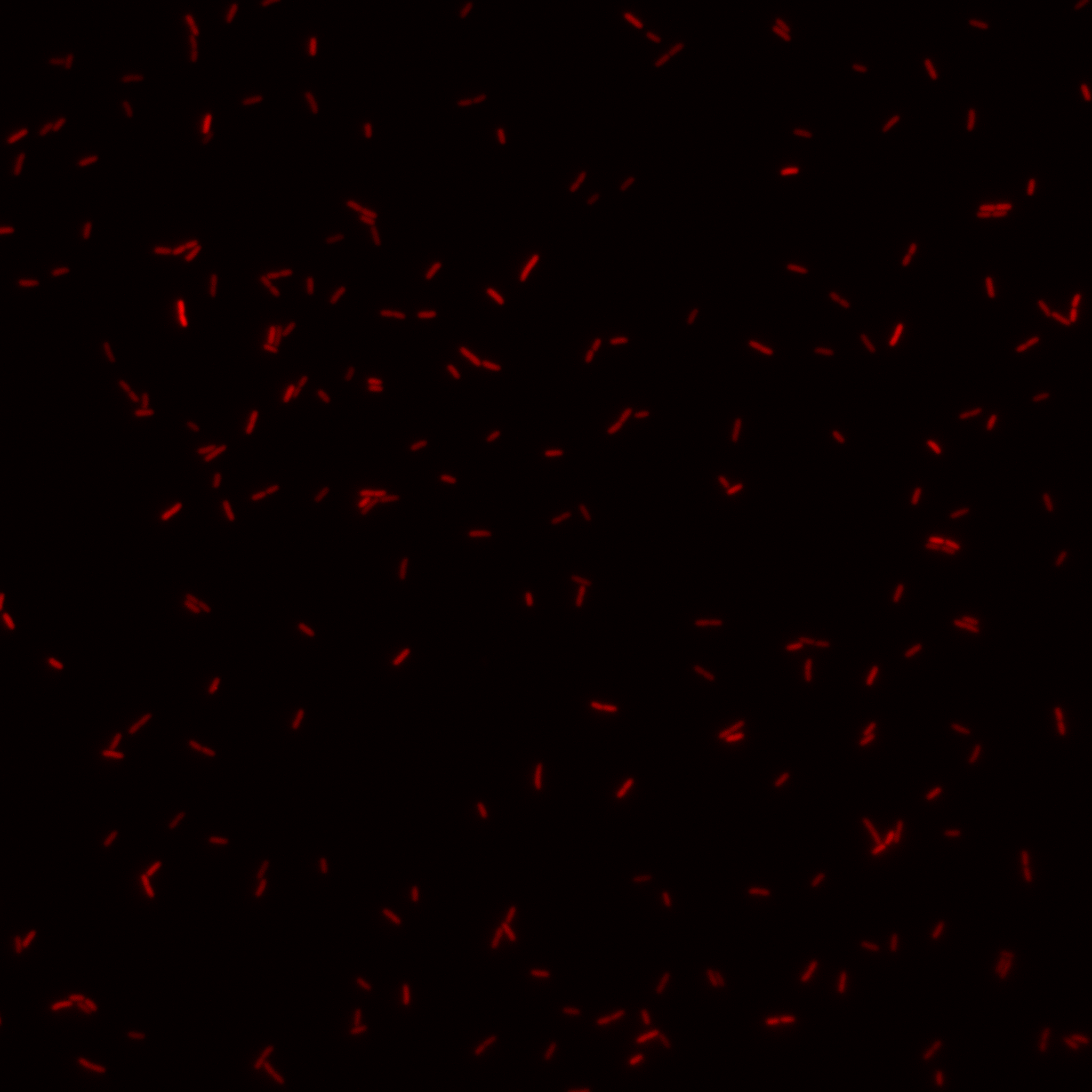

Supplement: Supplementary file 7 — Source data Fig. 5 [file 44318_2025_595_MOESM7_ESM.zip › Fig. 5/5A/5A_M(Gly)8-mCherry.tif]

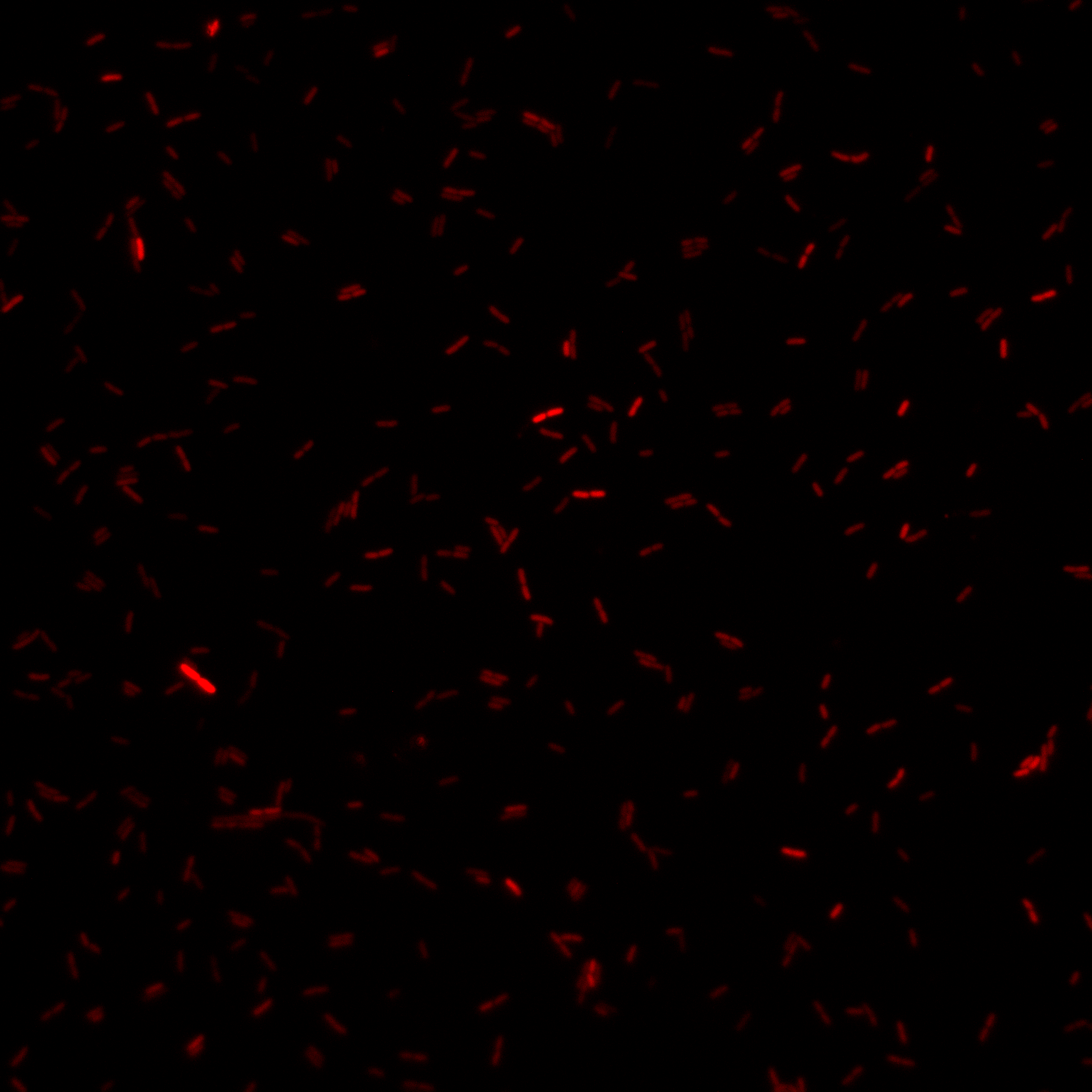

Supplement: Supplementary file 7 — Source data Fig. 5 [file 44318_2025_595_MOESM7_ESM.zip › Fig. 5/5A/5A_mCherry-Tls.tif]

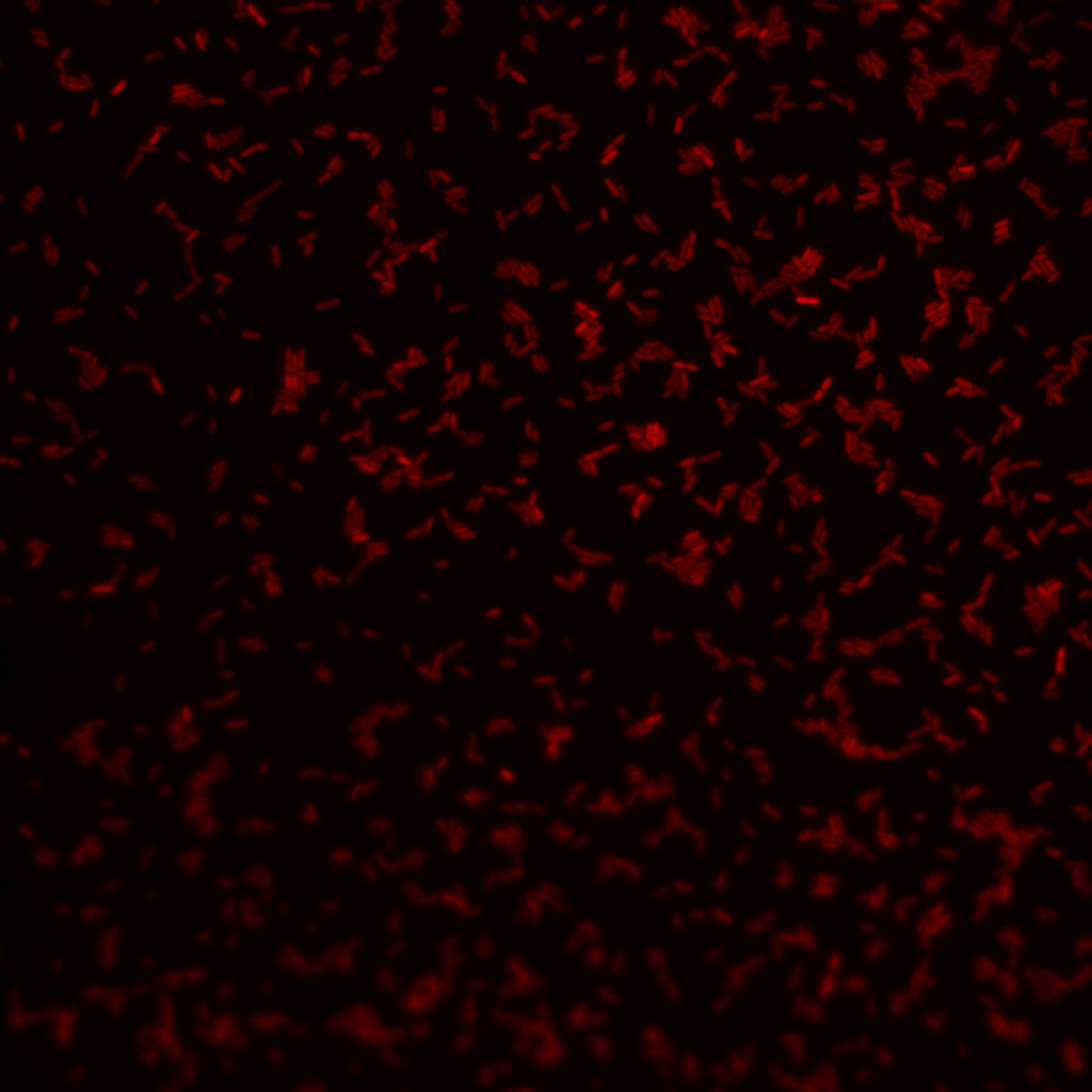

Supplement: Supplementary file 7 — Source data Fig. 5 [file 44318_2025_595_MOESM7_ESM.zip › Fig. 5/5A/5A_mCherry.tif]

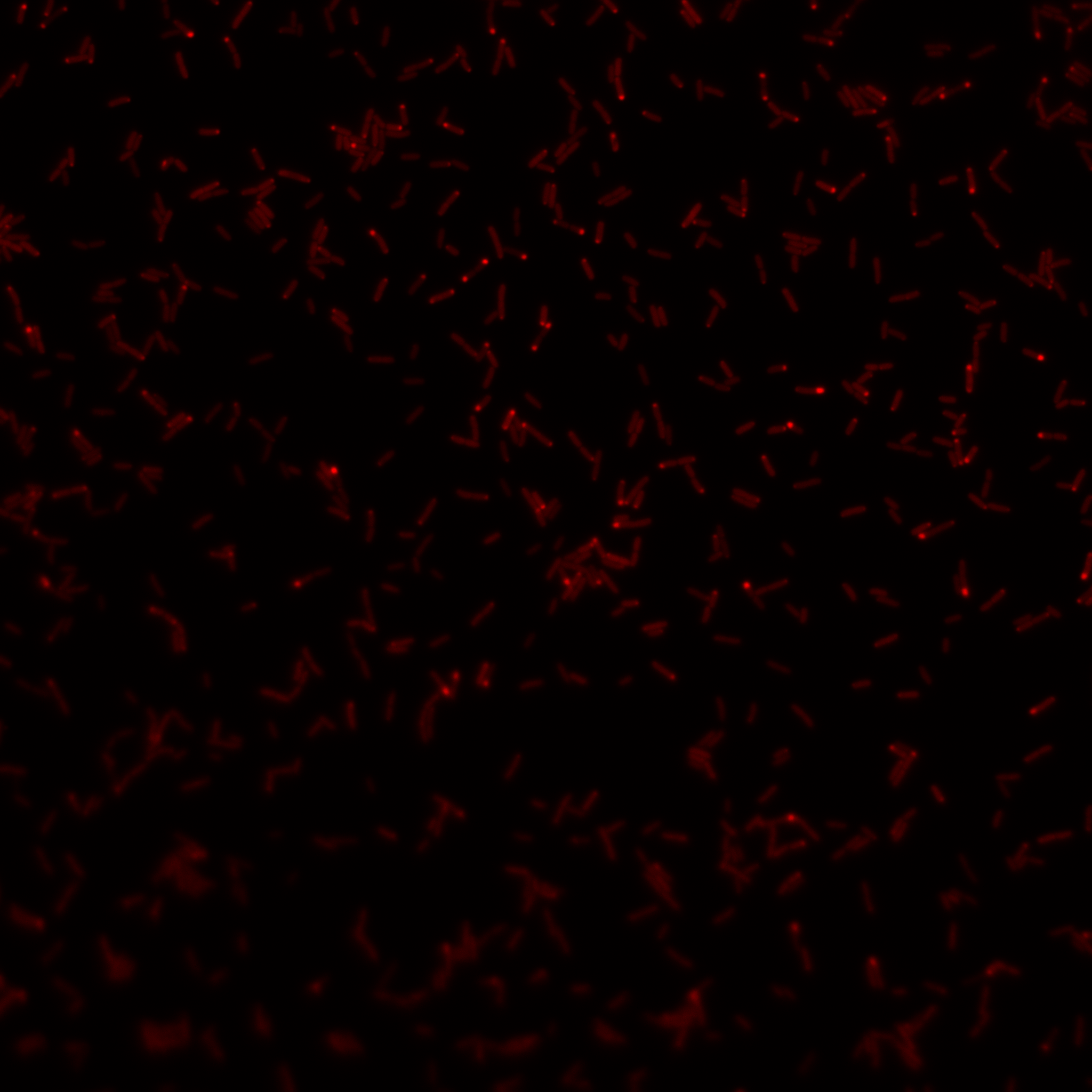

Supplement: Supplementary file 7 — Source data Fig. 5 [file 44318_2025_595_MOESM7_ESM.zip › Fig. 5/5A/5A_Tls-mCherry.tif]

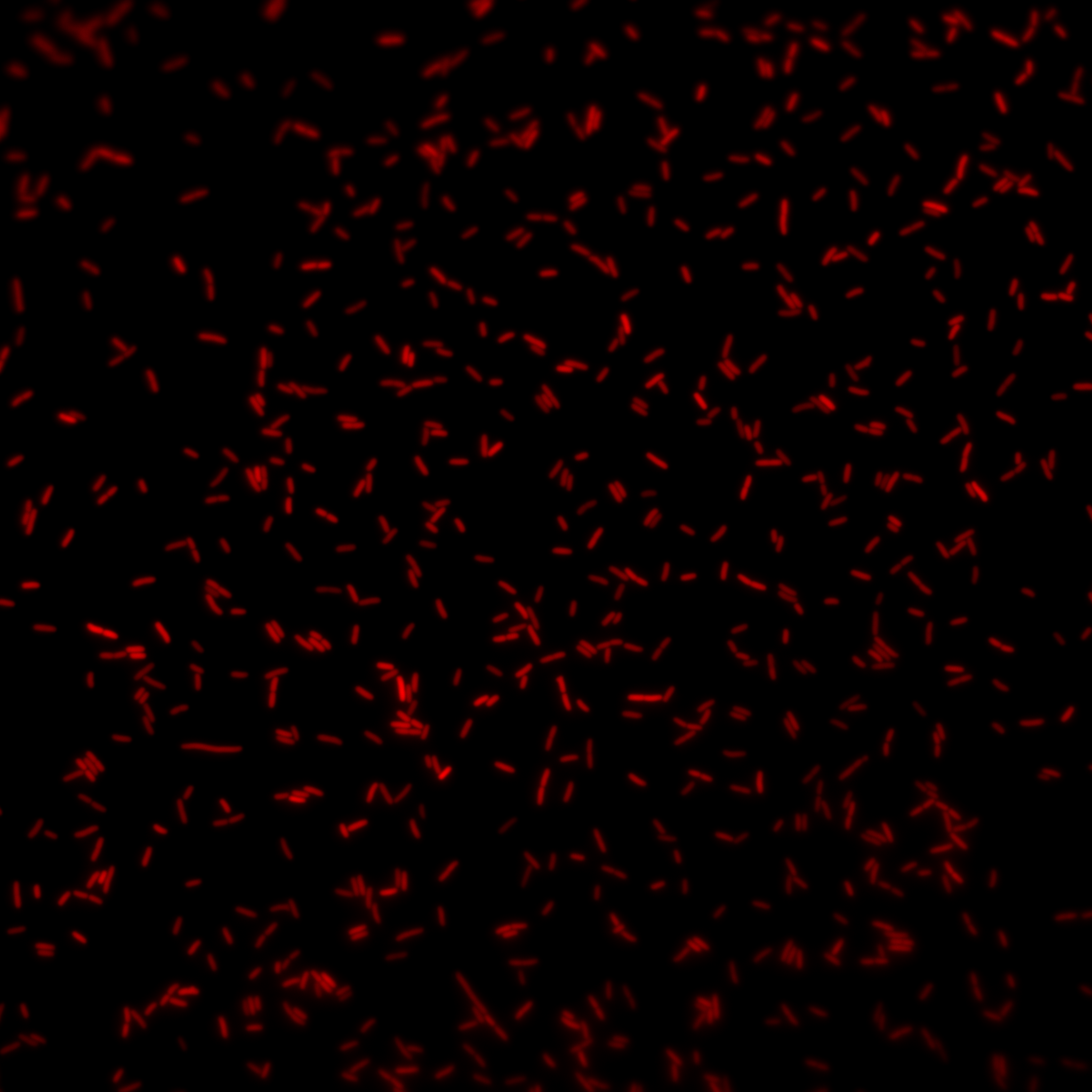

Supplement: Supplementary file 7 — Source data Fig. 5 [file 44318_2025_595_MOESM7_ESM.zip › Fig. 5/5A/5A_TlsN(asterisk)-mCherry.tif]

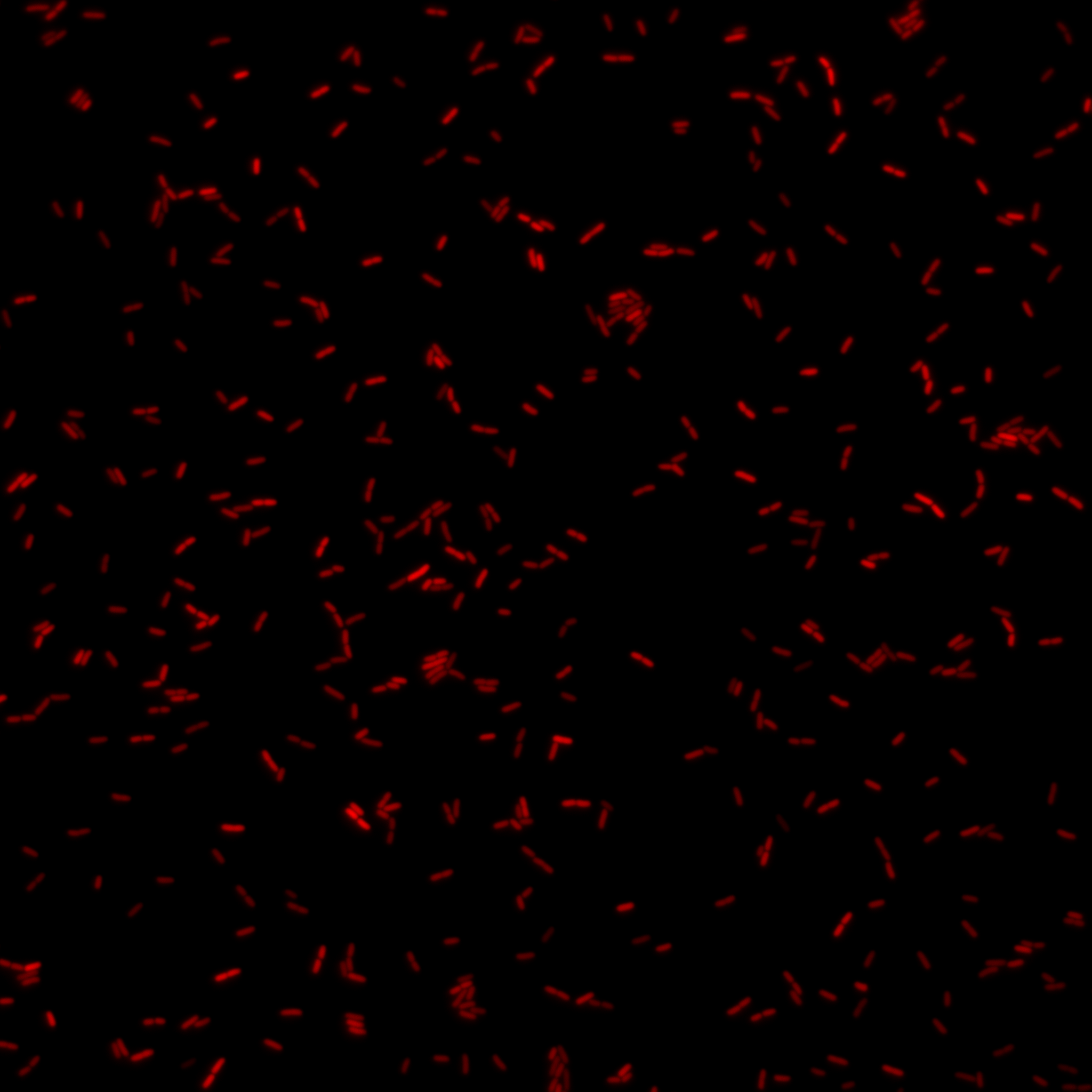

Supplement: Supplementary file 7 — Source data Fig. 5 [file 44318_2025_595_MOESM7_ESM.zip › Fig. 5/5A/5A_TlsN(R4D)-mCherry.tif]

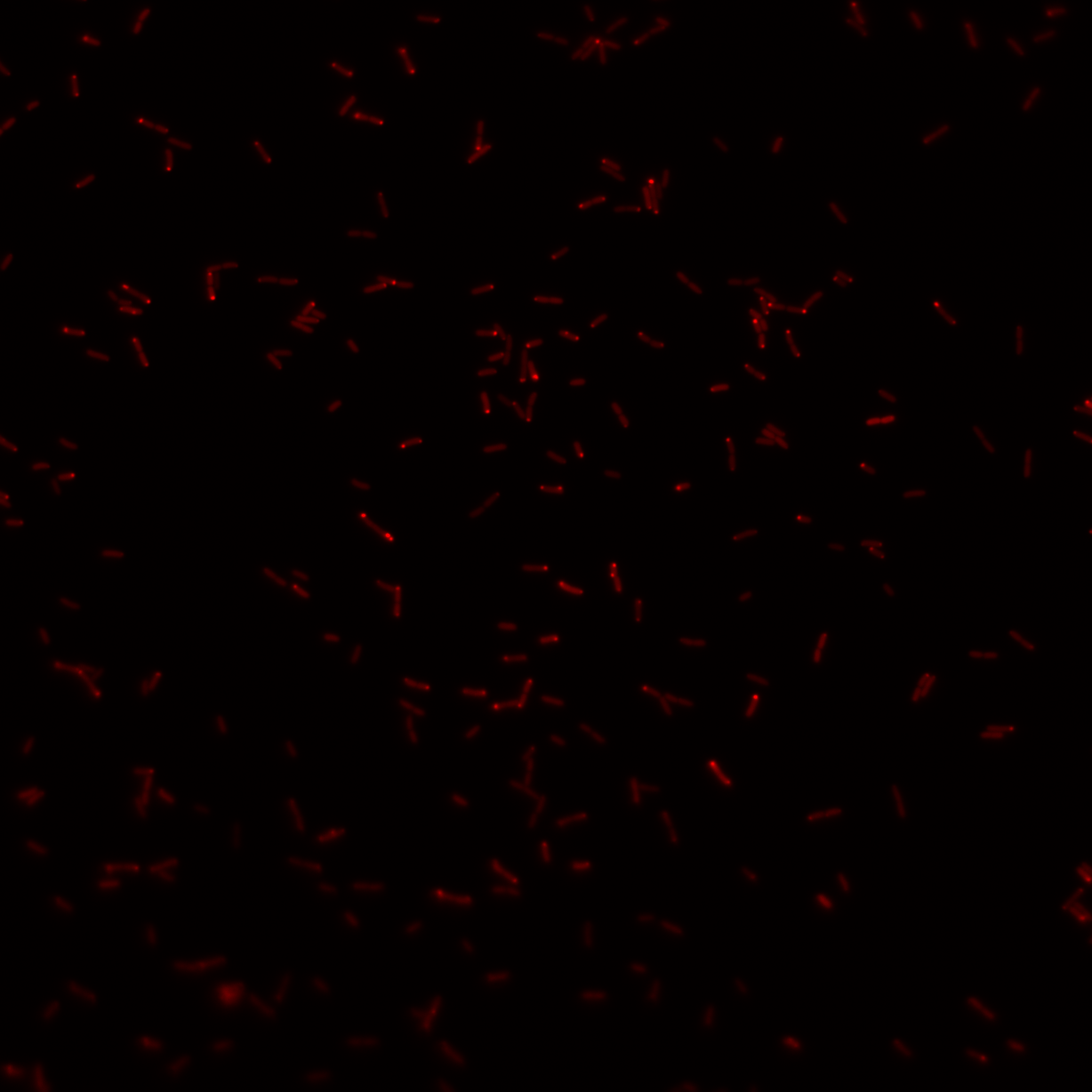

Supplement: Supplementary file 7 — Source data Fig. 5 [file 44318_2025_595_MOESM7_ESM.zip › Fig. 5/5A/5A_TlsN(R4K)-mCherry.tif]

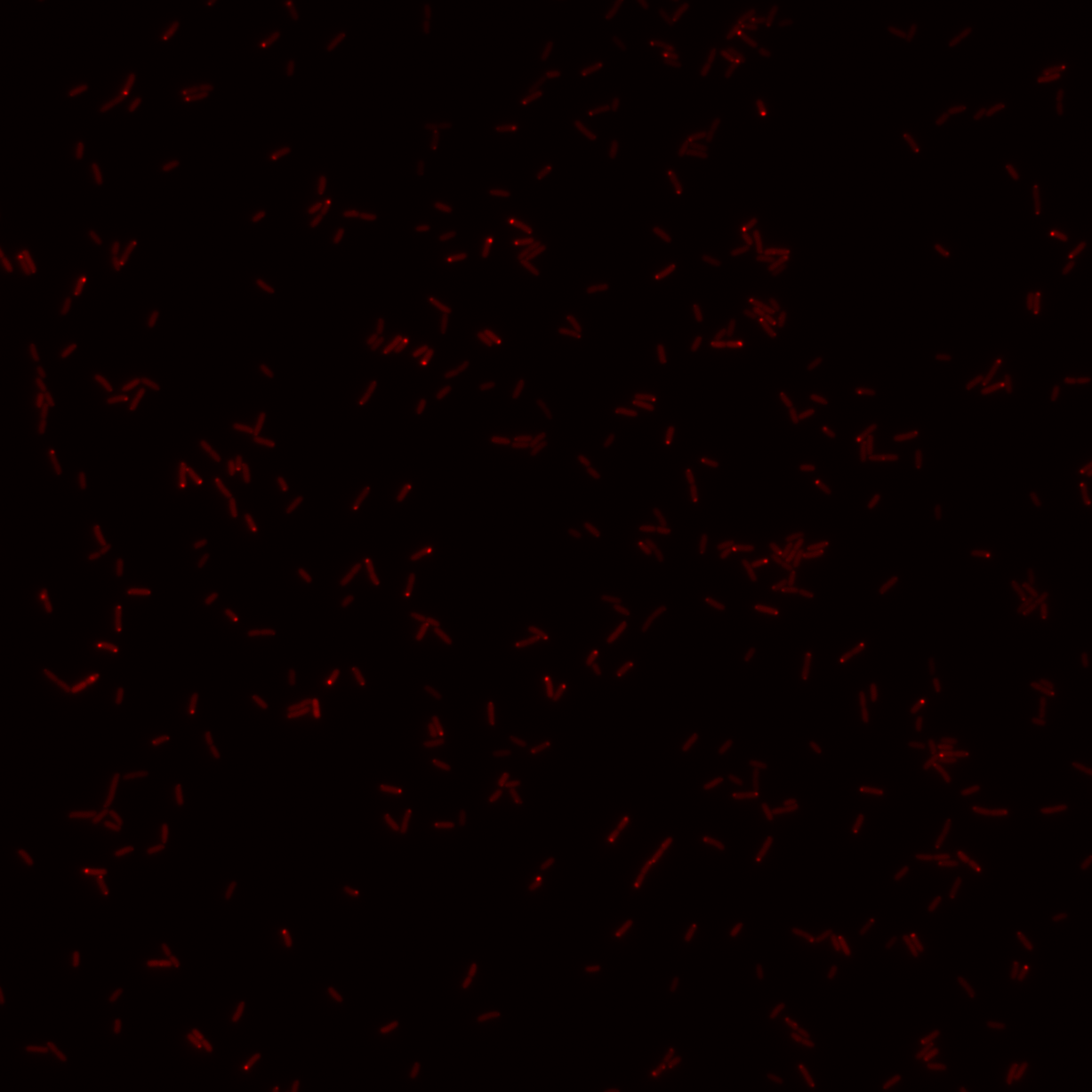

Supplement: Supplementary file 7 — Source data Fig. 5 [file 44318_2025_595_MOESM7_ESM.zip › Fig. 5/5A/5A_TlsN-mCherry.tif]

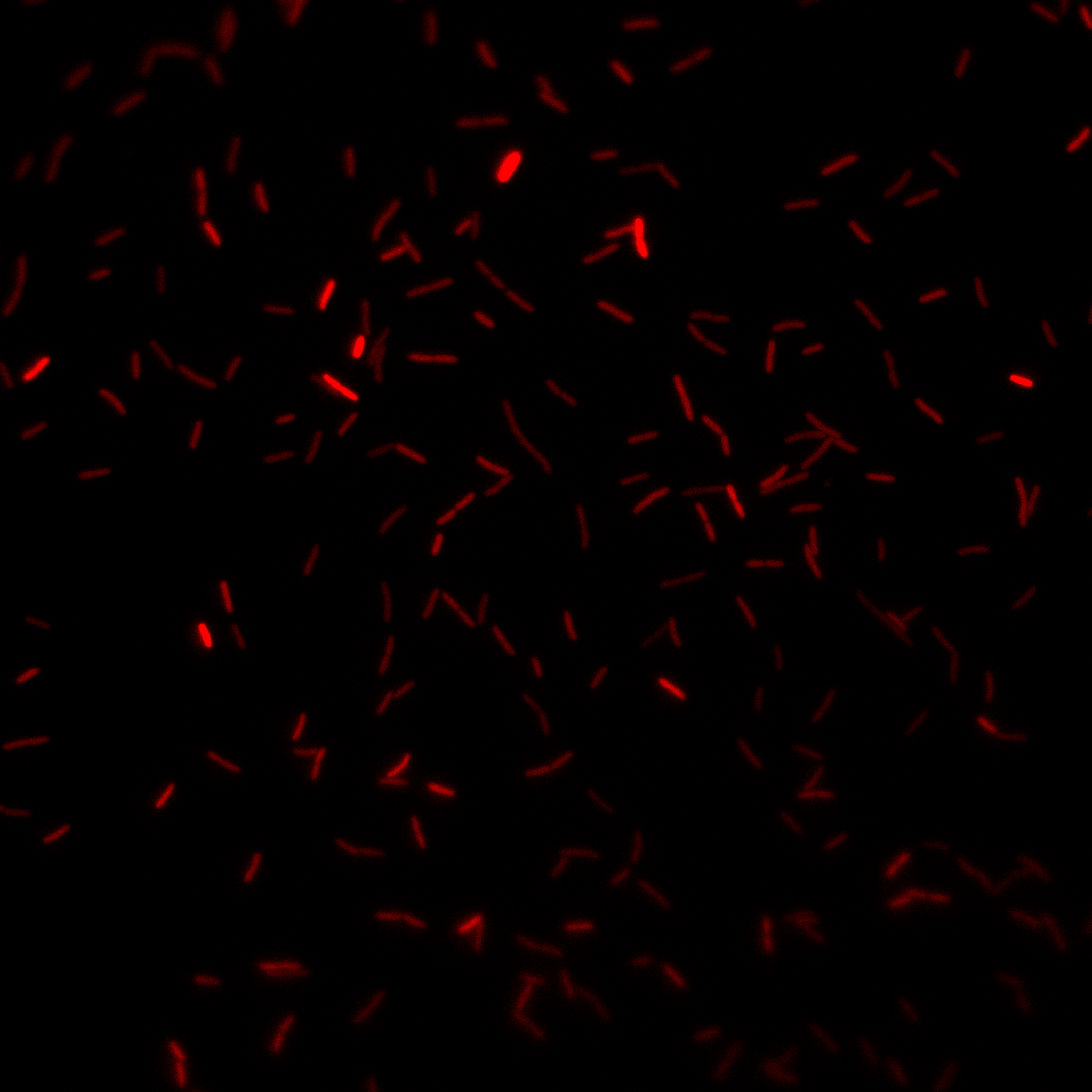

Supplement: Supplementary file 7 — Source data Fig. 5 [file 44318_2025_595_MOESM7_ESM.zip › Fig. 5/5A/5A_TlsN-mCherry_(Solid).tif]

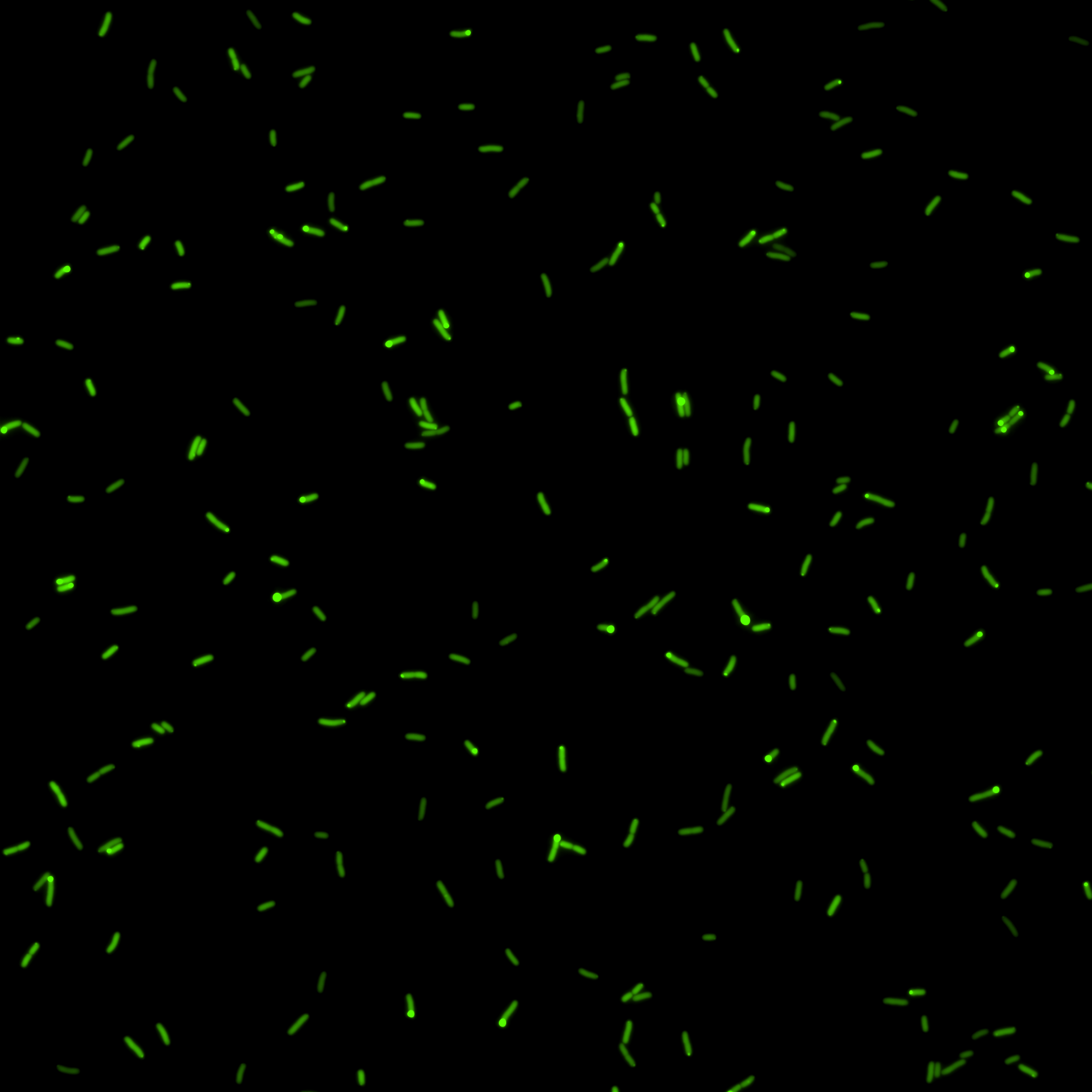

Supplement: Supplementary file 7 — Source data Fig. 5 [file 44318_2025_595_MOESM7_ESM.zip › Fig. 5/5C/5C_Liquid_N149Y.tif]

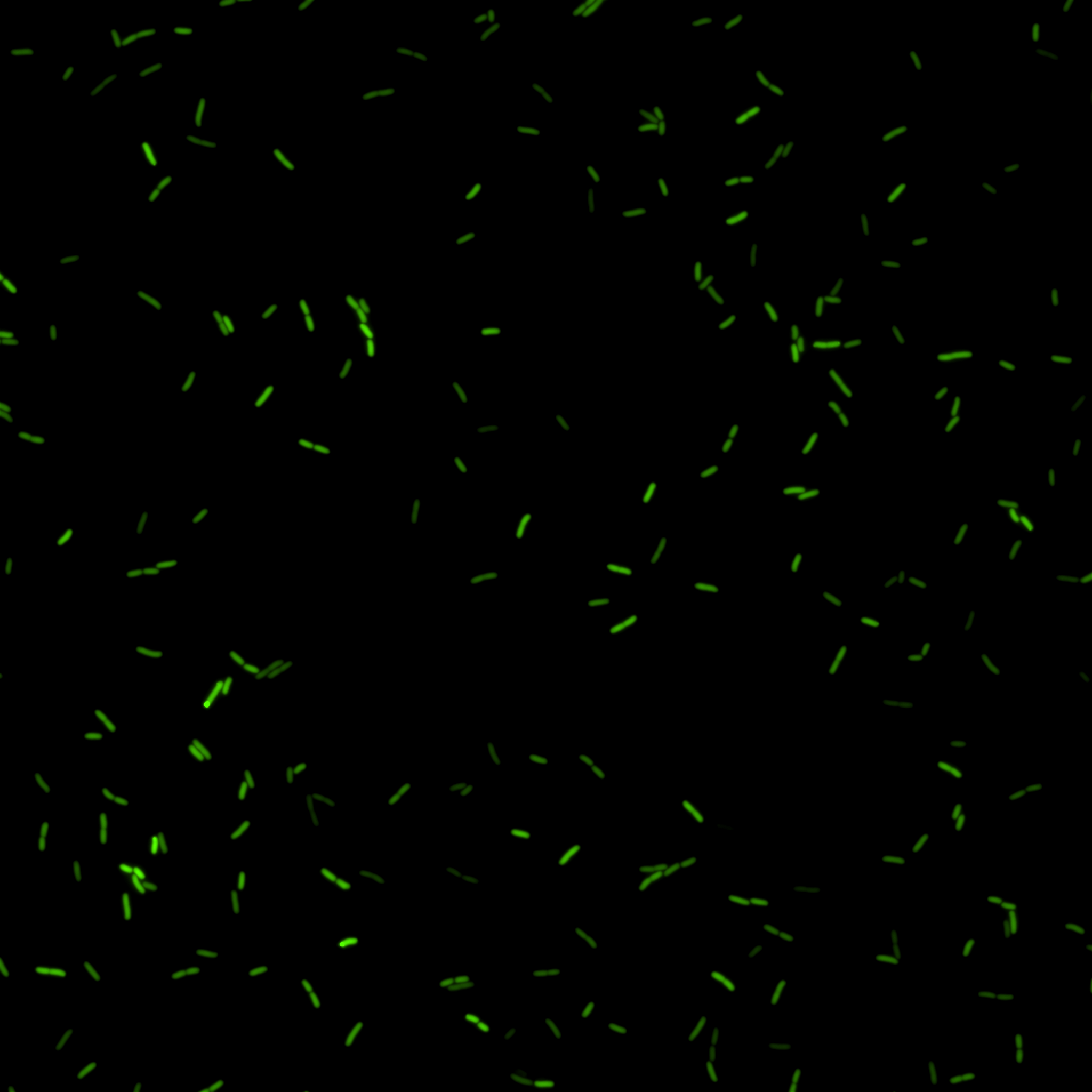

Supplement: Supplementary file 7 — Source data Fig. 5 [file 44318_2025_595_MOESM7_ESM.zip › Fig. 5/5C/5C_Liquid_original.tif]

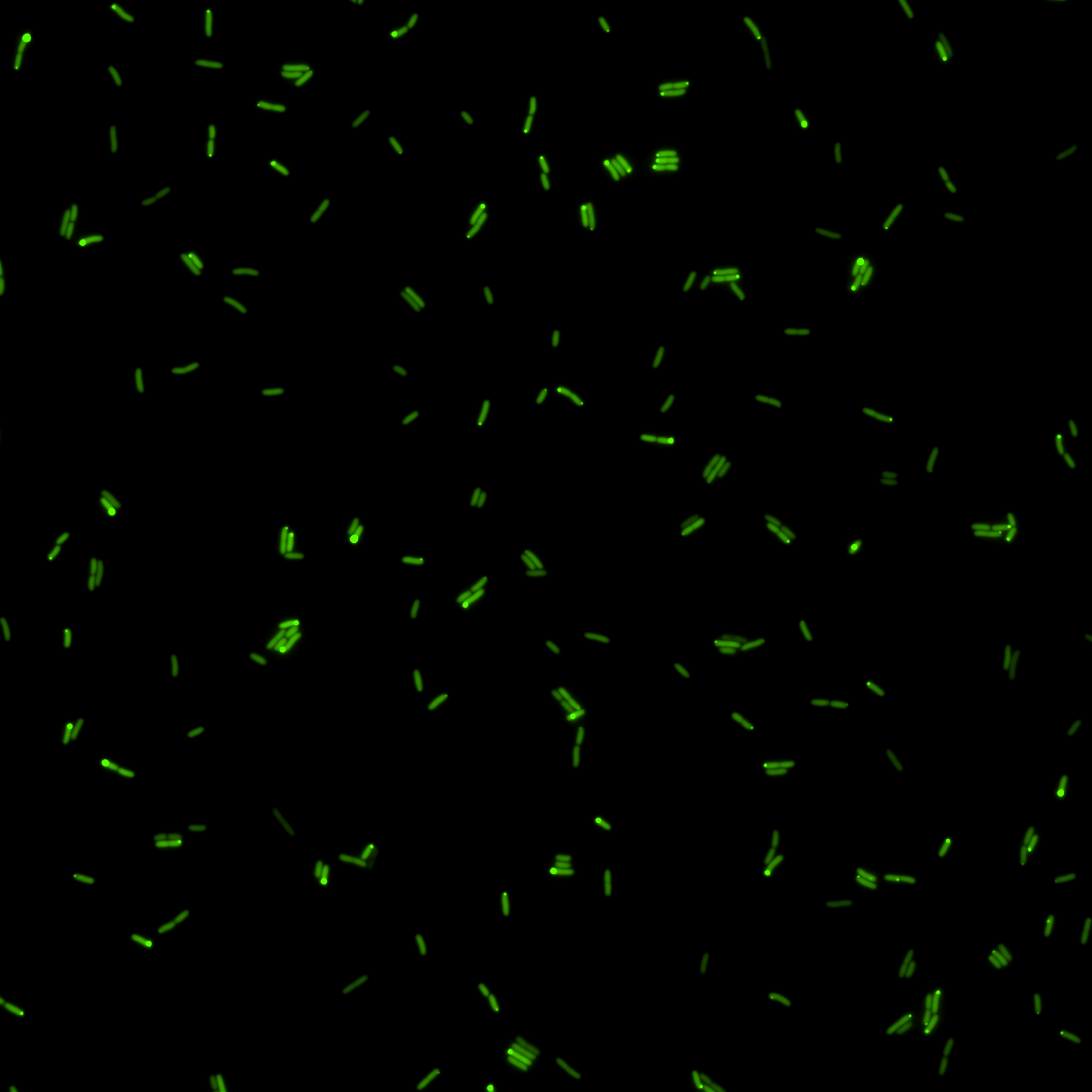

Supplement: Supplementary file 7 — Source data Fig. 5 [file 44318_2025_595_MOESM7_ESM.zip › Fig. 5/5C/5C_Liquid_R206A.tif]

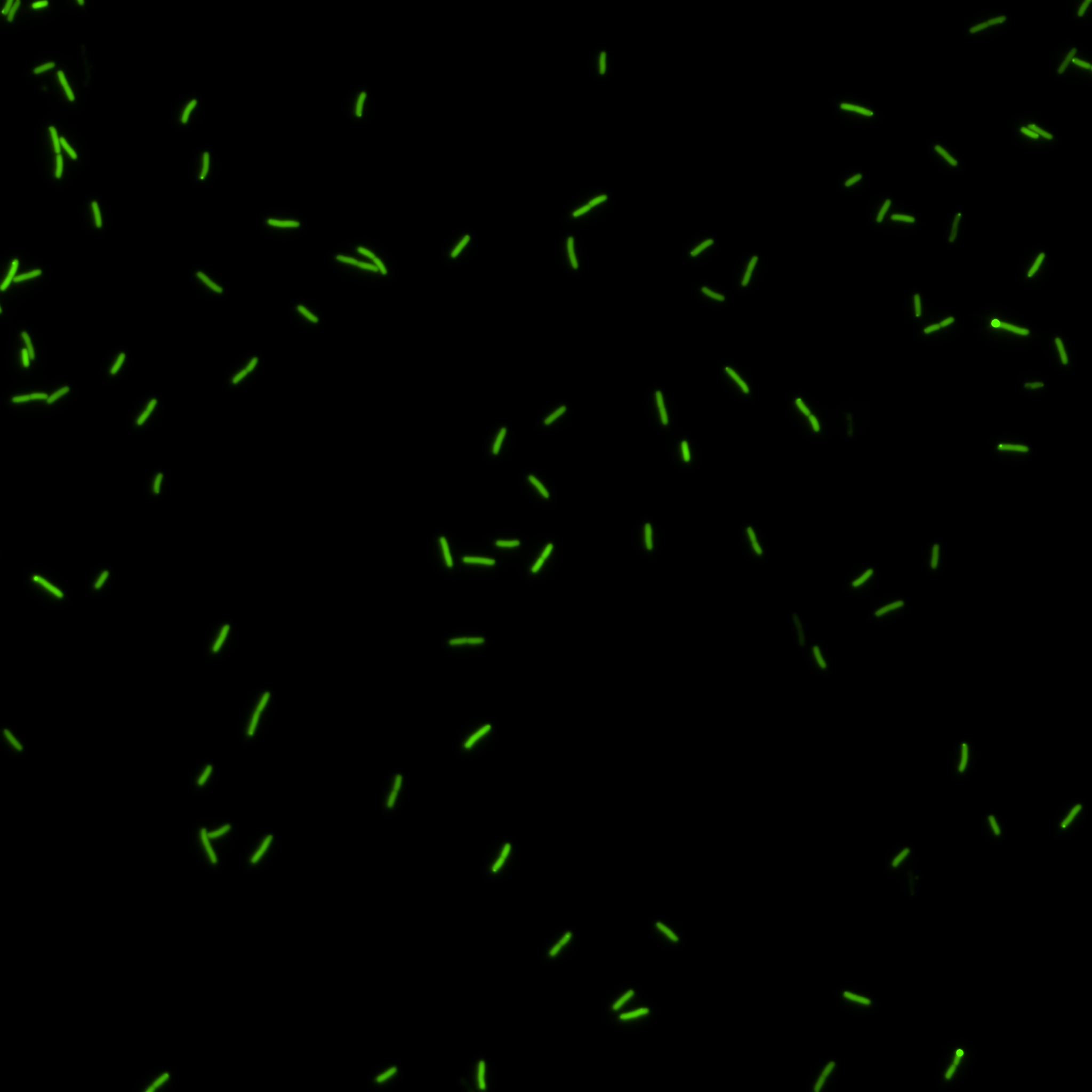

Supplement: Supplementary file 7 — Source data Fig. 5 [file 44318_2025_595_MOESM7_ESM.zip › Fig. 5/5C/5C_Solid_N149Y.tif]

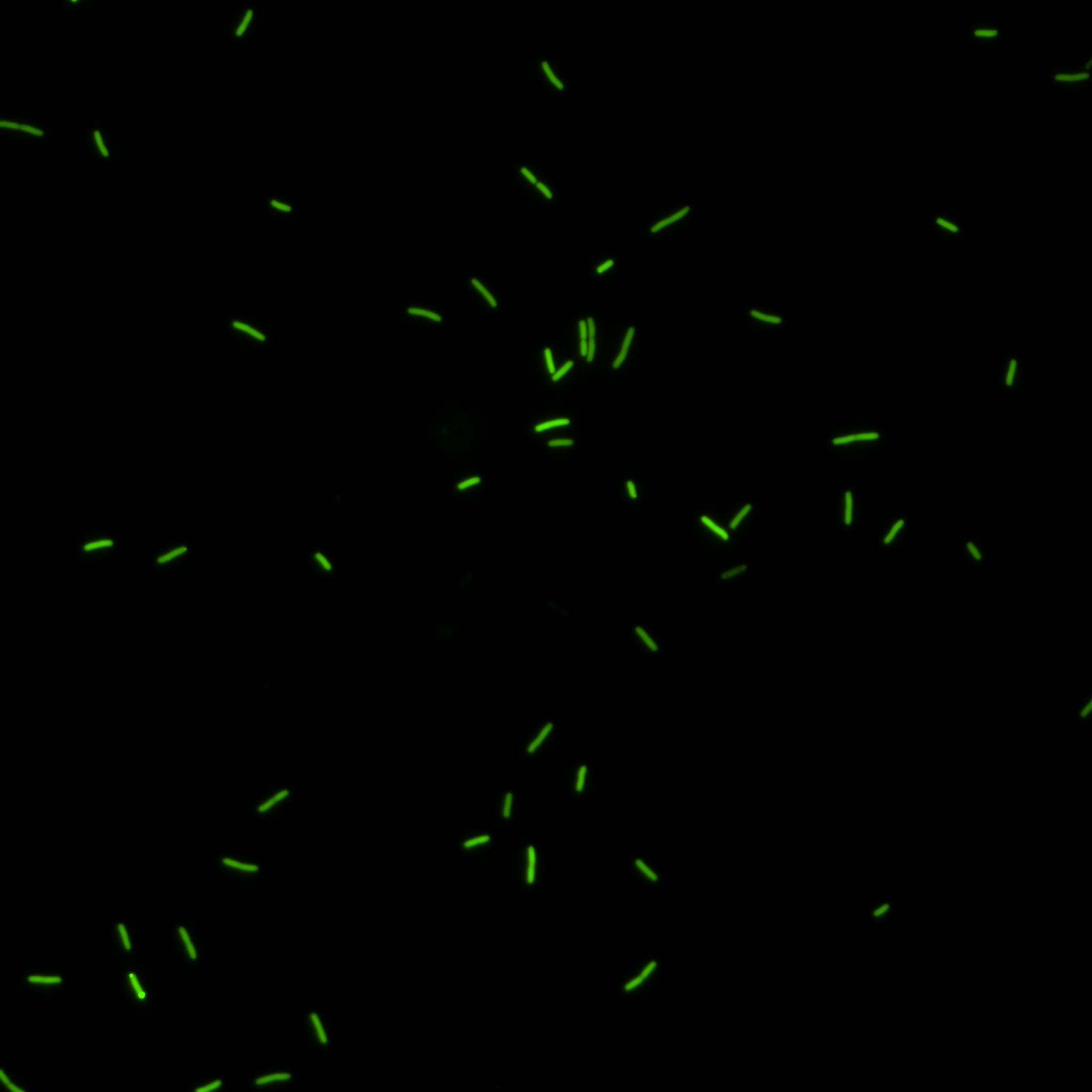

Supplement: Supplementary file 7 — Source data Fig. 5 [file 44318_2025_595_MOESM7_ESM.zip › Fig. 5/5C/5C_Solid_original.tif]

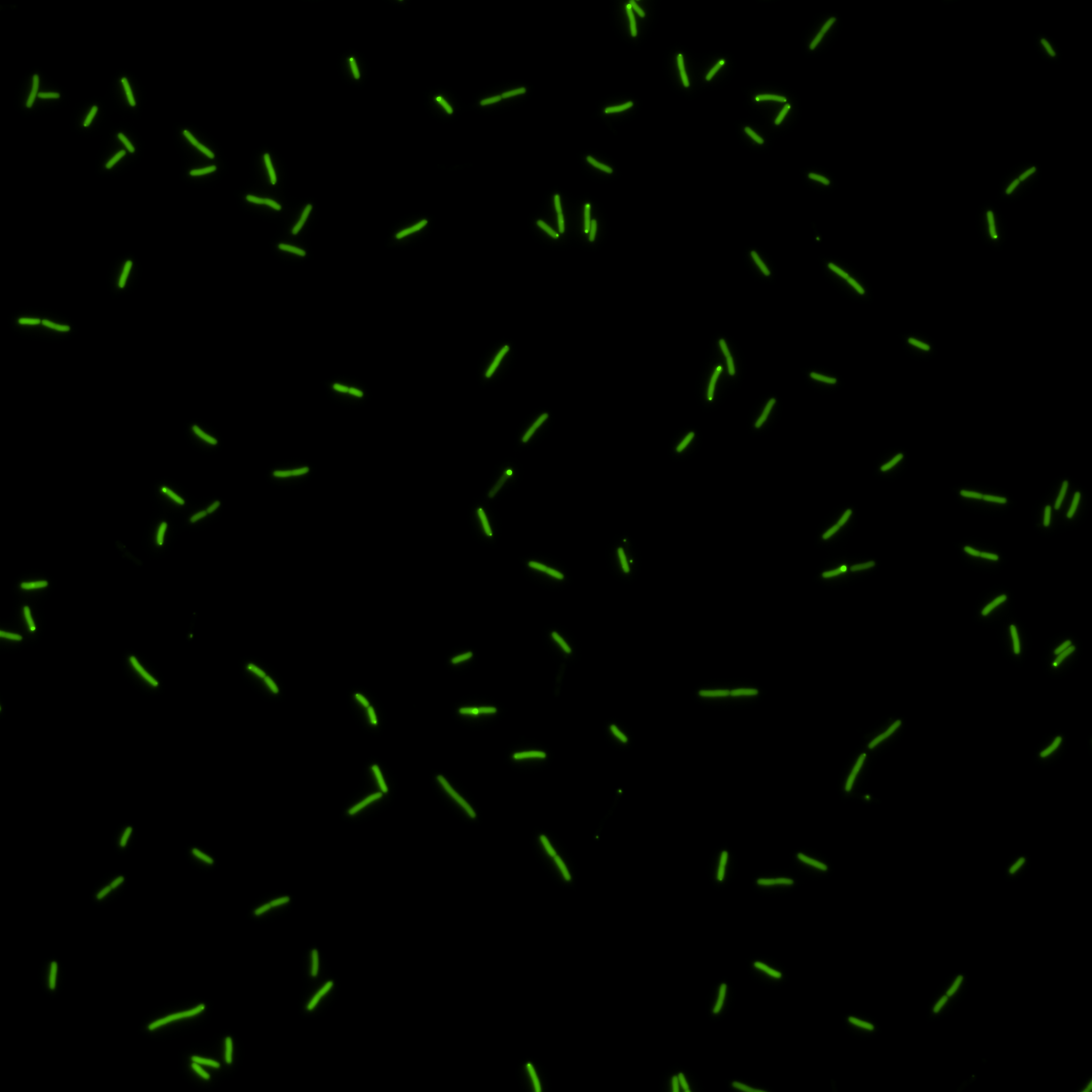

Supplement: Supplementary file 7 — Source data Fig. 5 [file 44318_2025_595_MOESM7_ESM.zip › Fig. 5/5C/5C_Solid_R206A.tif]

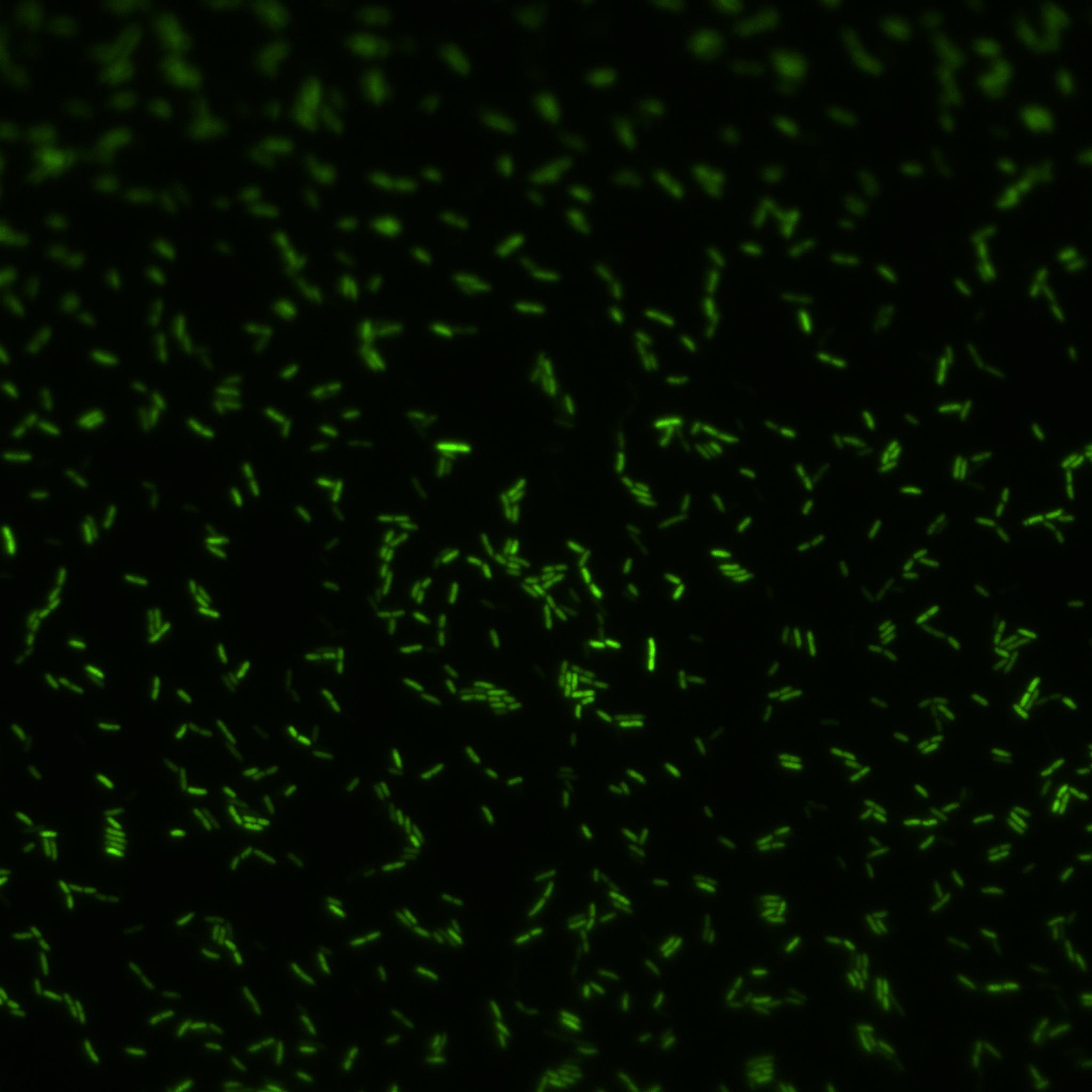

Supplement: Supplementary file 8 — Source data Fig. 6 [file 44318_2025_595_MOESM8_ESM.zip › Fig. 6/6A/6A_FlhDC-sfGFP.tif]

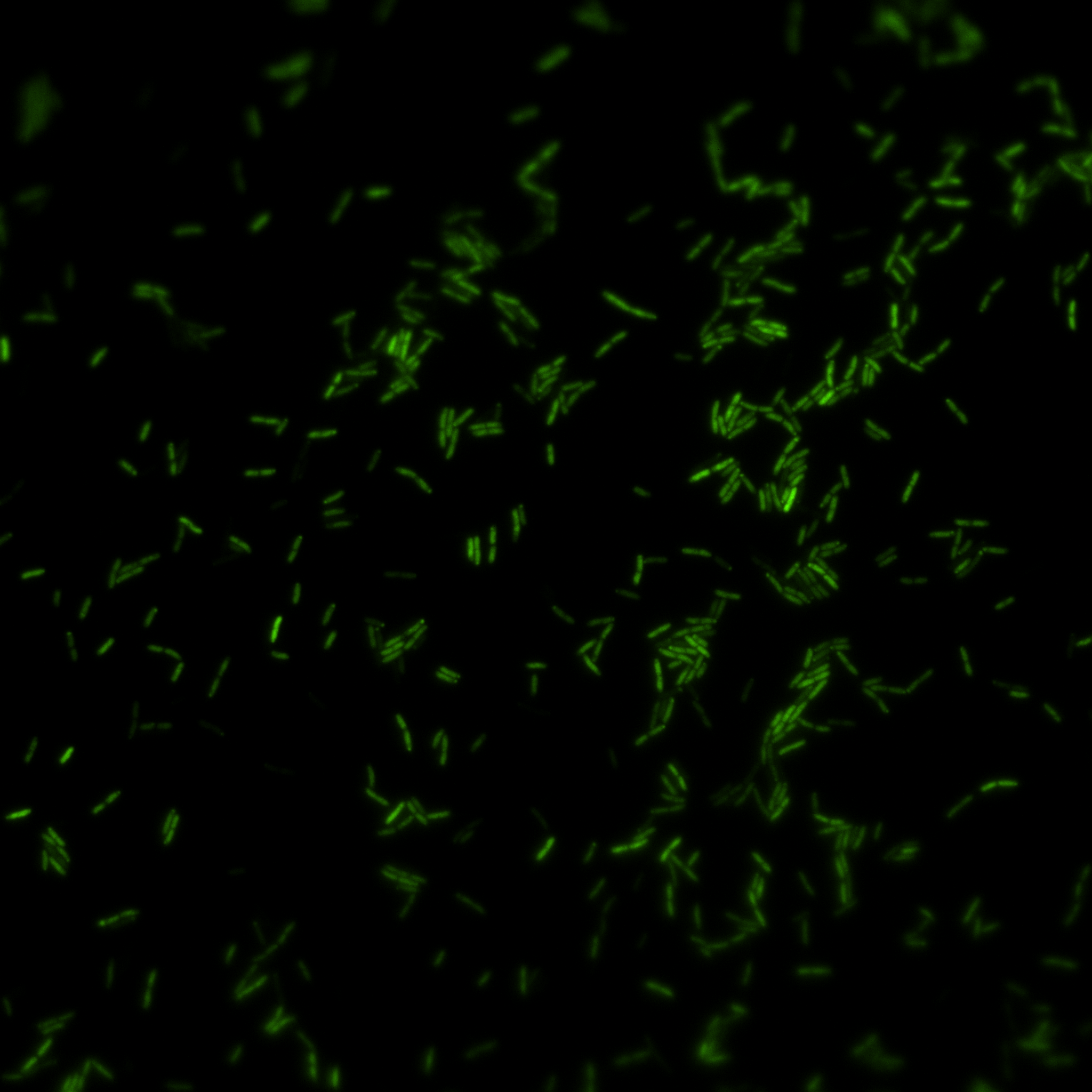

Supplement: Supplementary file 8 — Source data Fig. 6 [file 44318_2025_595_MOESM8_ESM.zip › Fig. 6/6A/6A_Tls+FlhDC-sfGFP.tif]

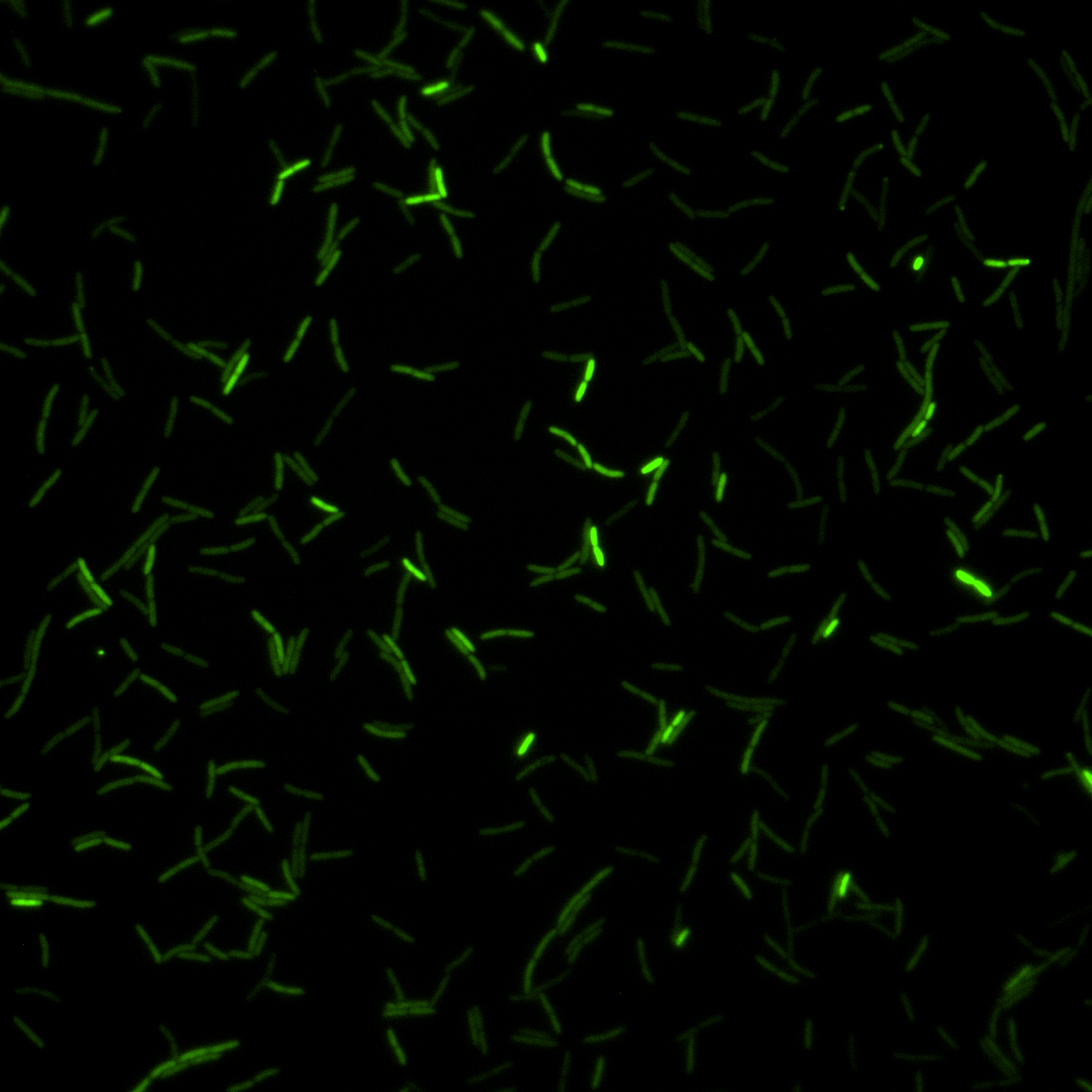

Supplement: Supplementary file 8 — Source data Fig. 6 [file 44318_2025_595_MOESM8_ESM.zip › Fig. 6/6B/6B_Tls+FlhDC-sfGFP_(Solid).tif]

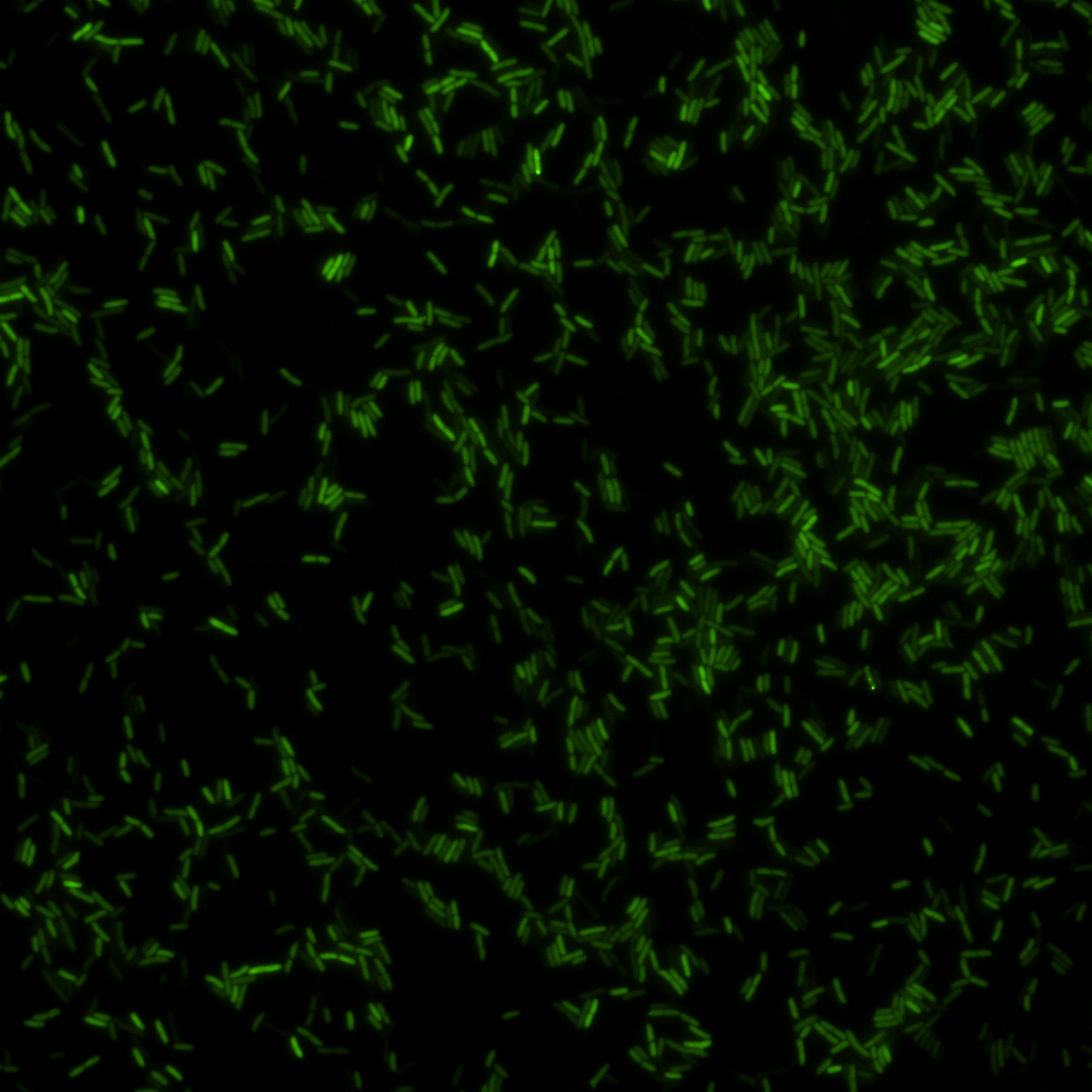

Supplement: Supplementary file 8 — Source data Fig. 6 [file 44318_2025_595_MOESM8_ESM.zip › Fig. 6/6D/6D_FlhDC-sfGFP mut..tif]

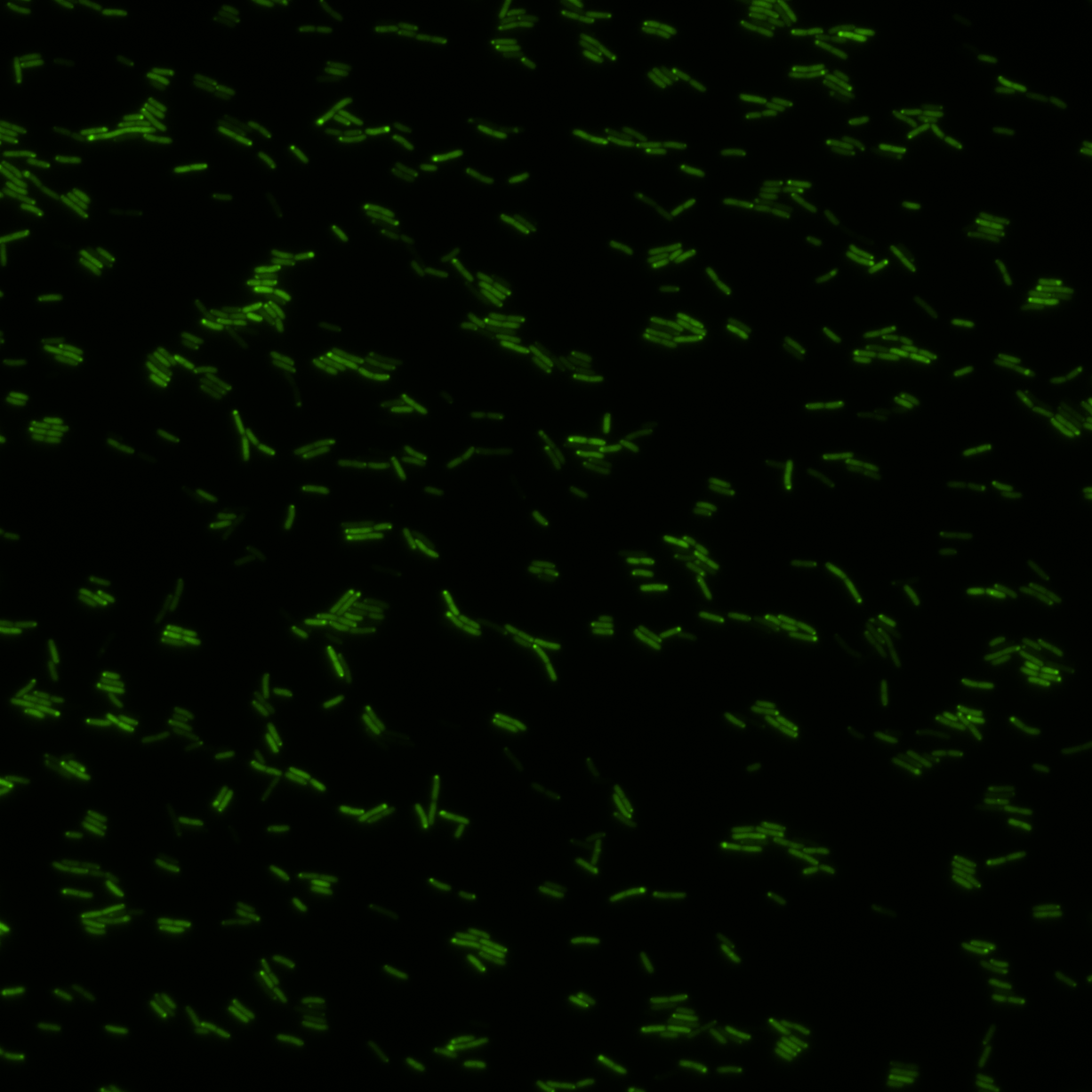

Supplement: Supplementary file 8 — Source data Fig. 6 [file 44318_2025_595_MOESM8_ESM.zip › Fig. 6/6D/6D_Tls+FlhDC-sfGFP mut..tif]

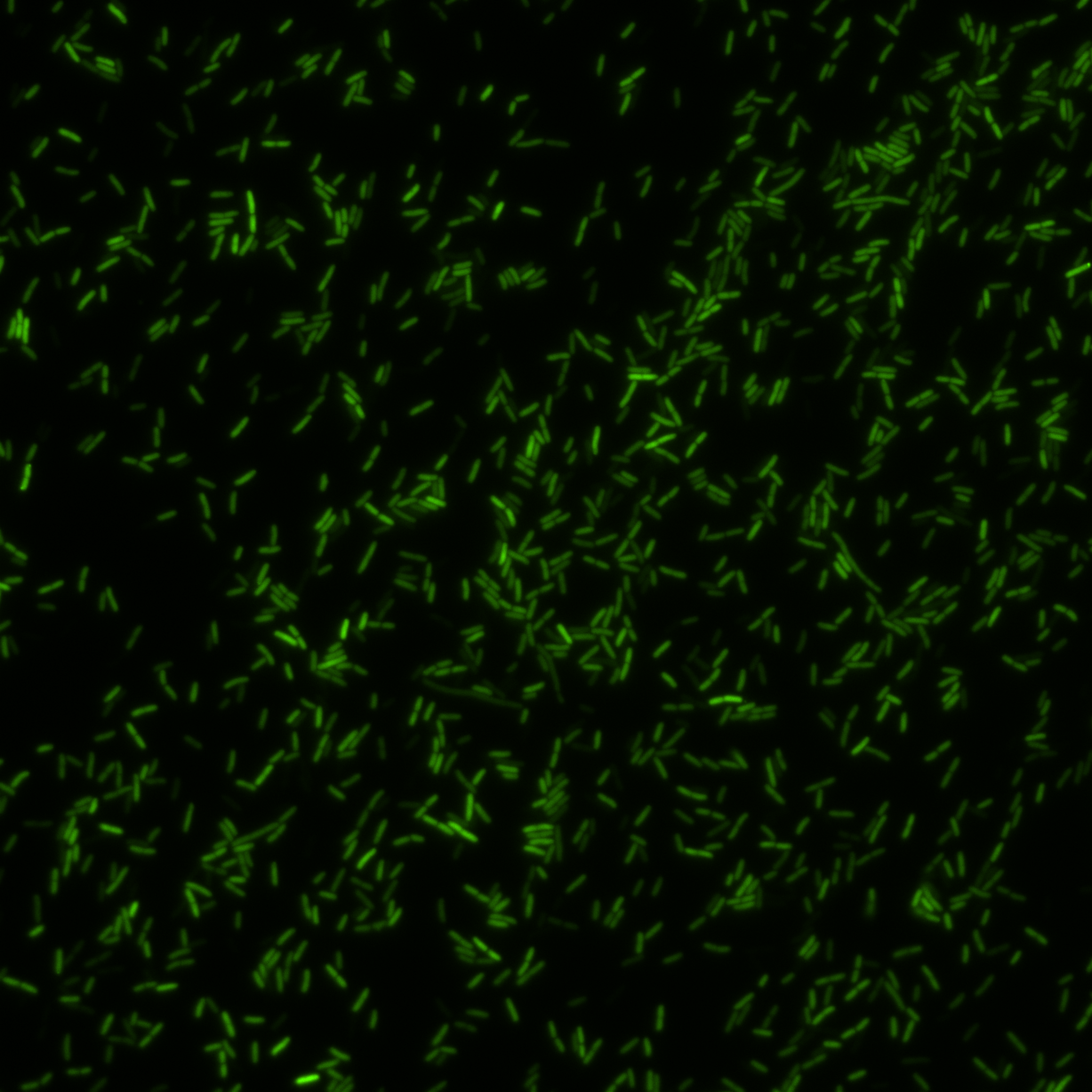

Supplement: Supplementary file 8 — Source data Fig. 6 [file 44318_2025_595_MOESM8_ESM.zip › Fig. 6/6D/6D_Trg+FlhDC-sfGFP mut..tif]

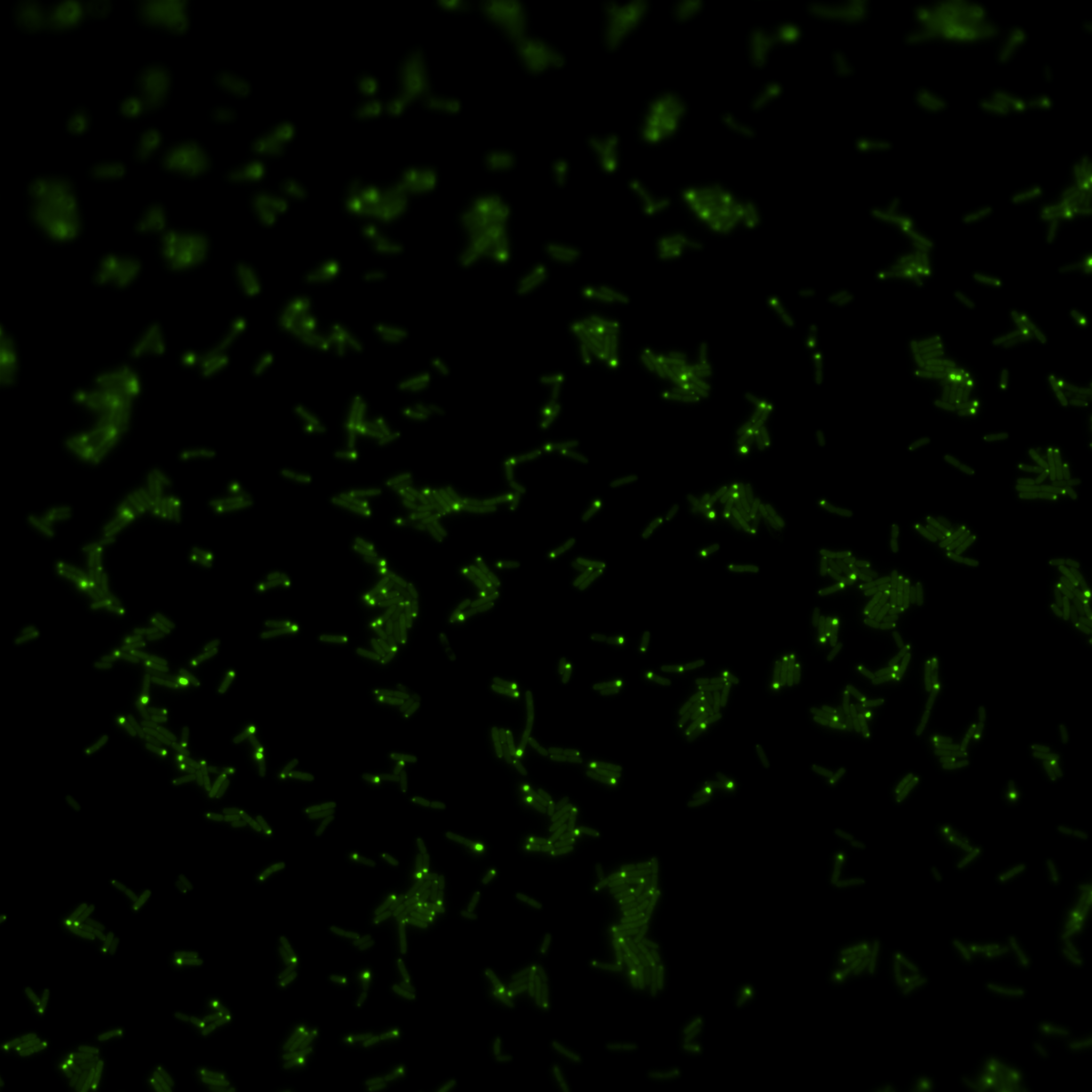

Supplement: Supplementary file 11 — Figure EV3 Source Data [file 44318_2025_595_MOESM11_ESM.zip › Fig. EV3/3E/3E.tif]

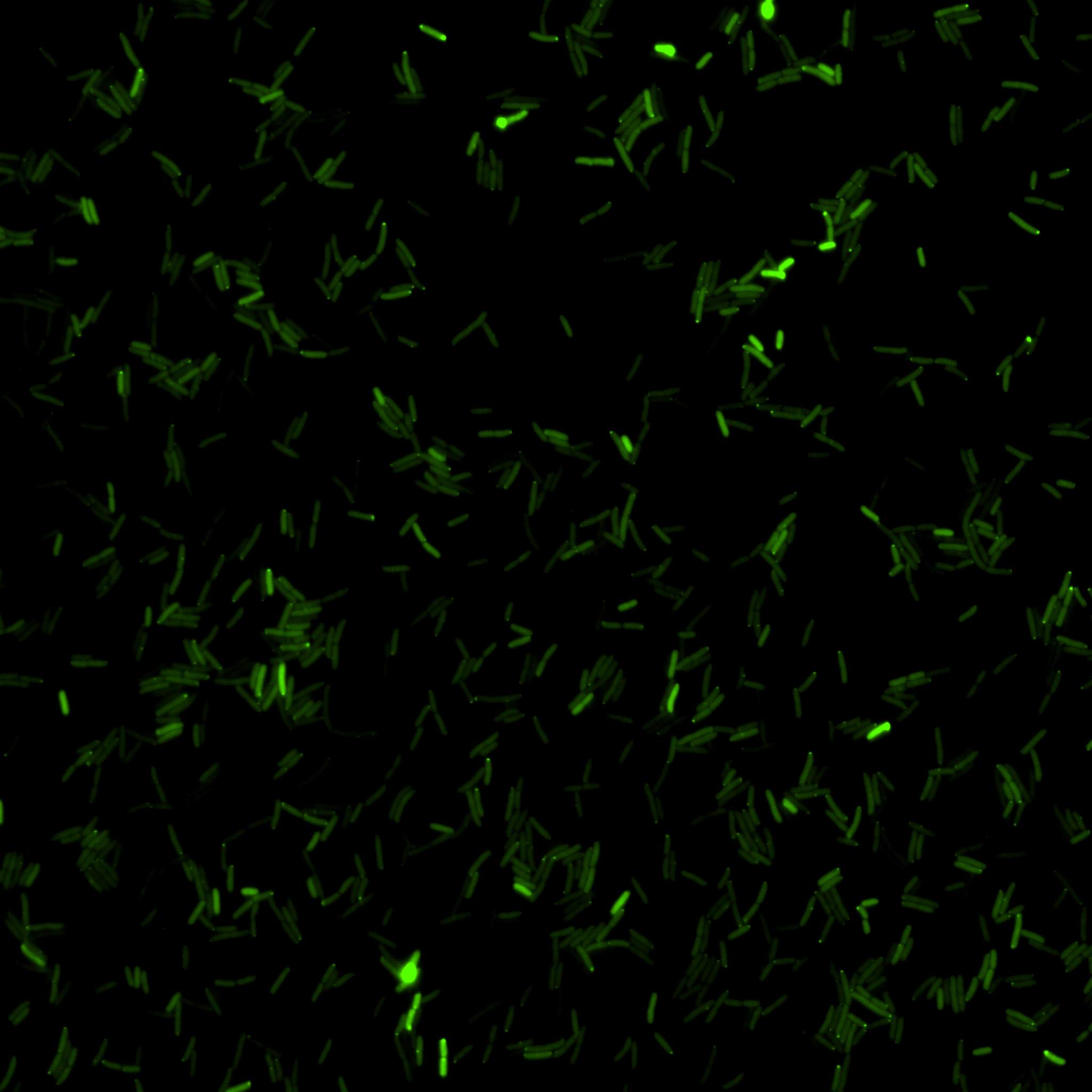

Supplement: Supplementary file 11 — Figure EV3 Source Data [file 44318_2025_595_MOESM11_ESM.zip › Fig. EV3/3H/3H.tif]

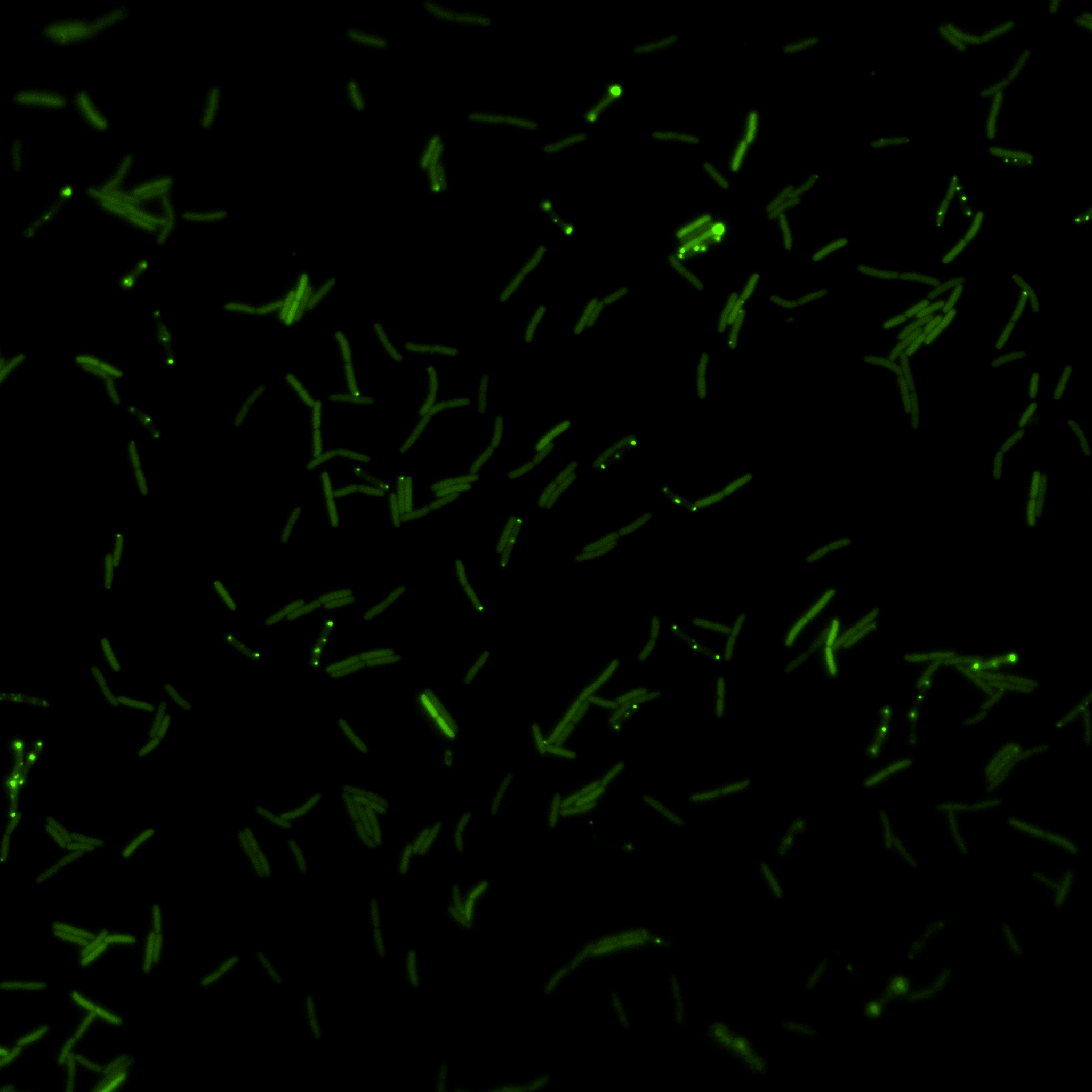

Supplement: Supplementary file 11 — Figure EV3 Source Data [file 44318_2025_595_MOESM11_ESM.zip › Fig. EV3/3F/3F.tif]

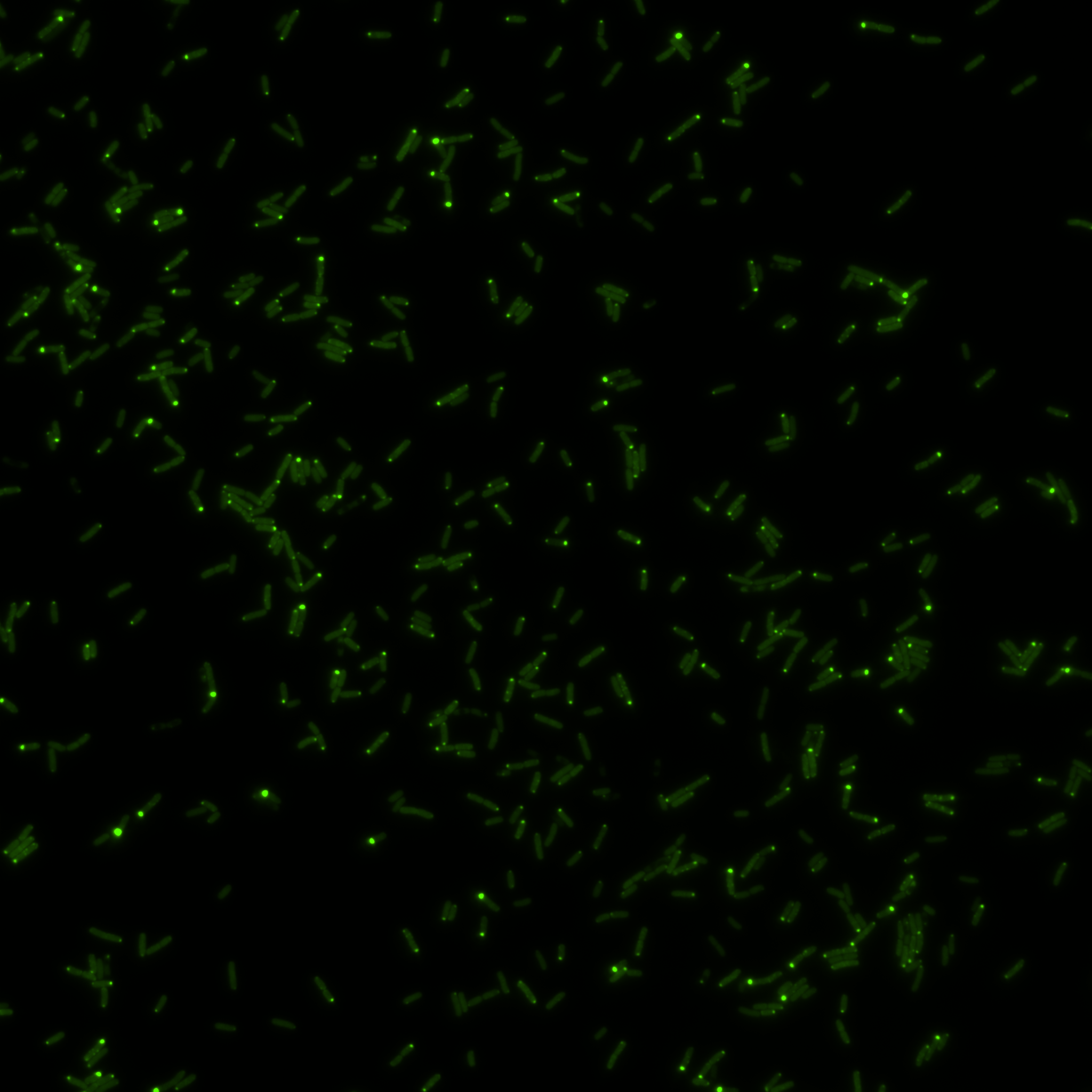

Supplement: Supplementary file 11 — Figure EV3 Source Data [file 44318_2025_595_MOESM11_ESM.zip › Fig. EV3/3G/3G.tif]

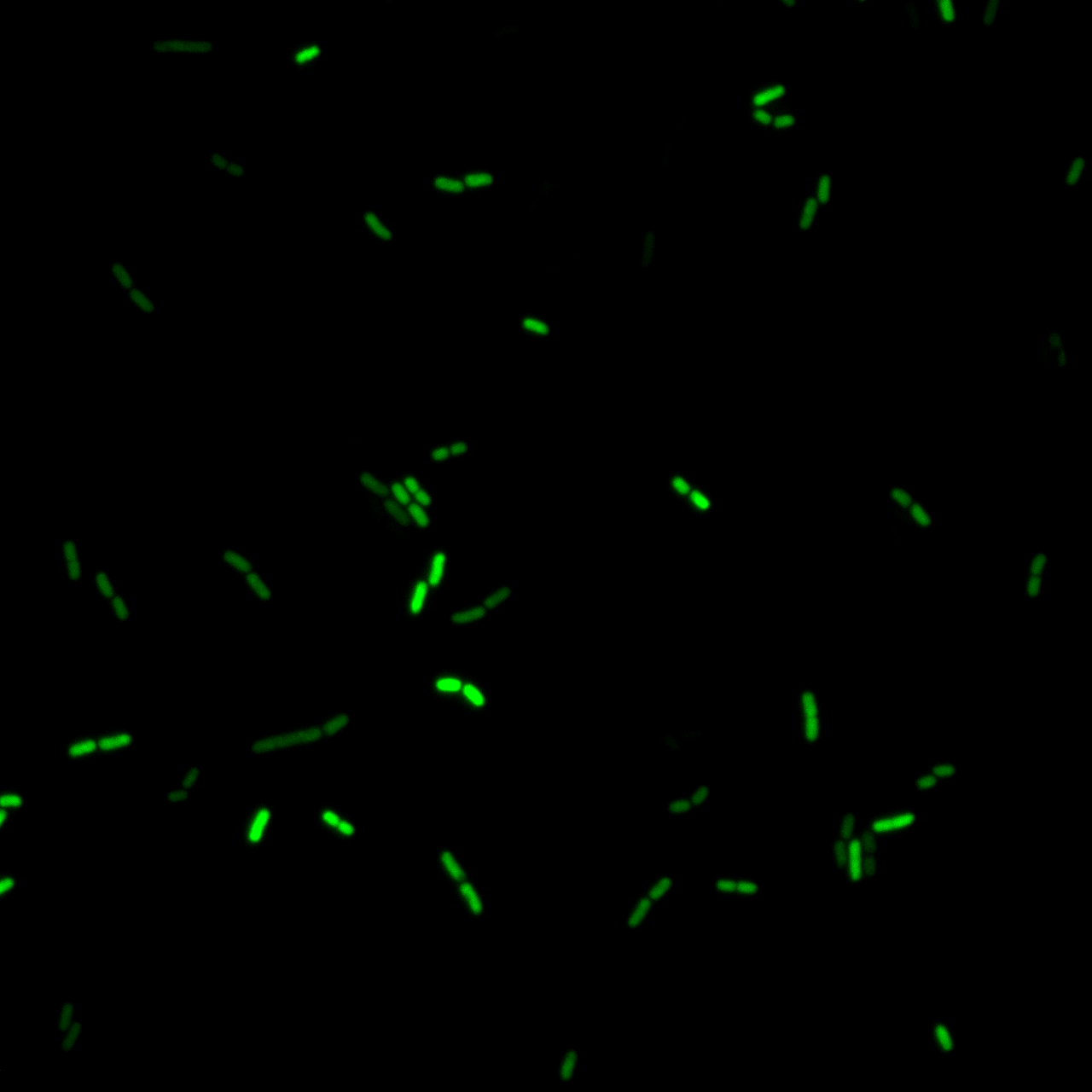

Supplement: Supplementary file 12 — Figure EV4 Source Data [file 44318_2025_595_MOESM12_ESM.zip › Fig. EV4/4E/4E_GFP_up.tif]

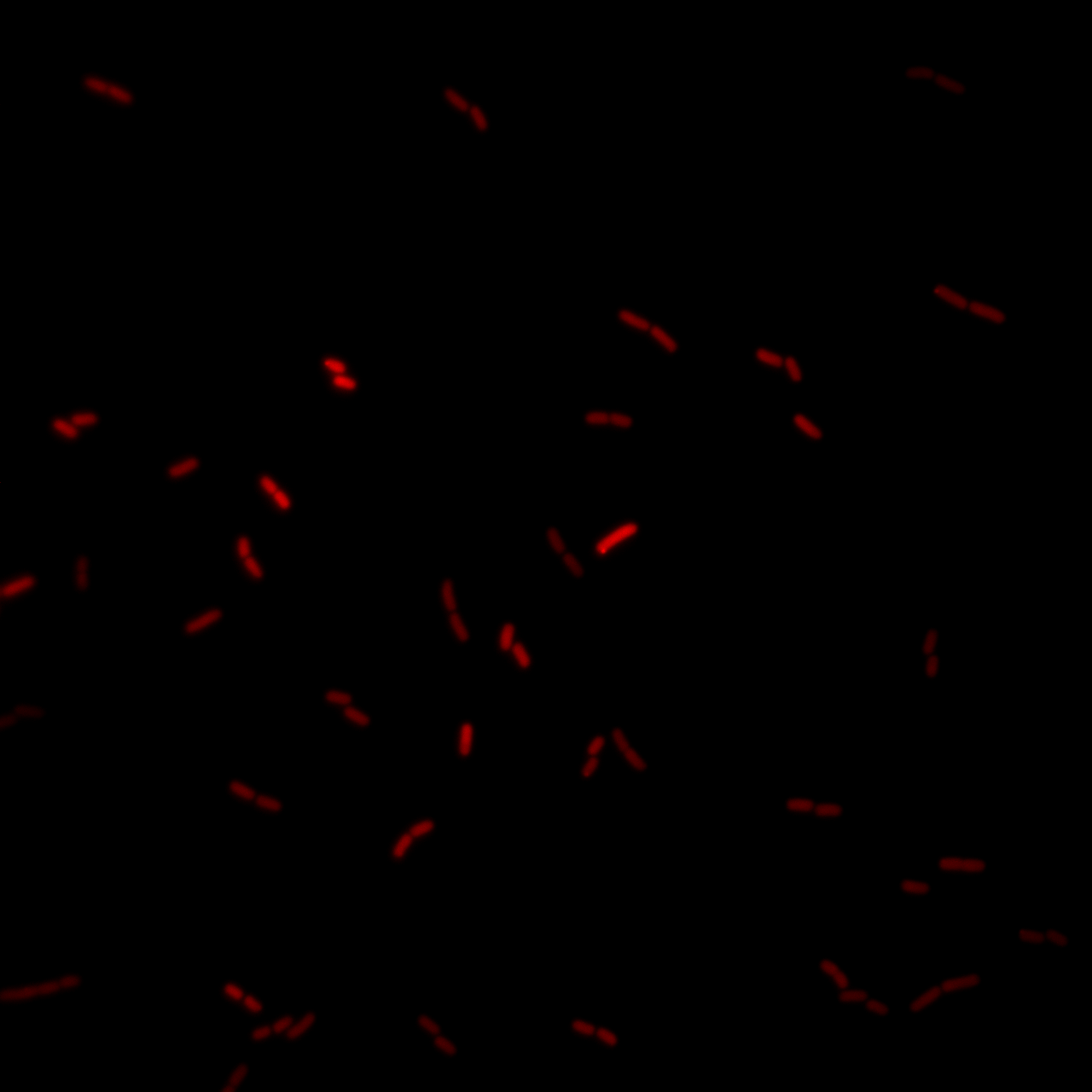

Supplement: Supplementary file 12 — Figure EV4 Source Data [file 44318_2025_595_MOESM12_ESM.zip › Fig. EV4/4E/4E_mCherry_down.tif]

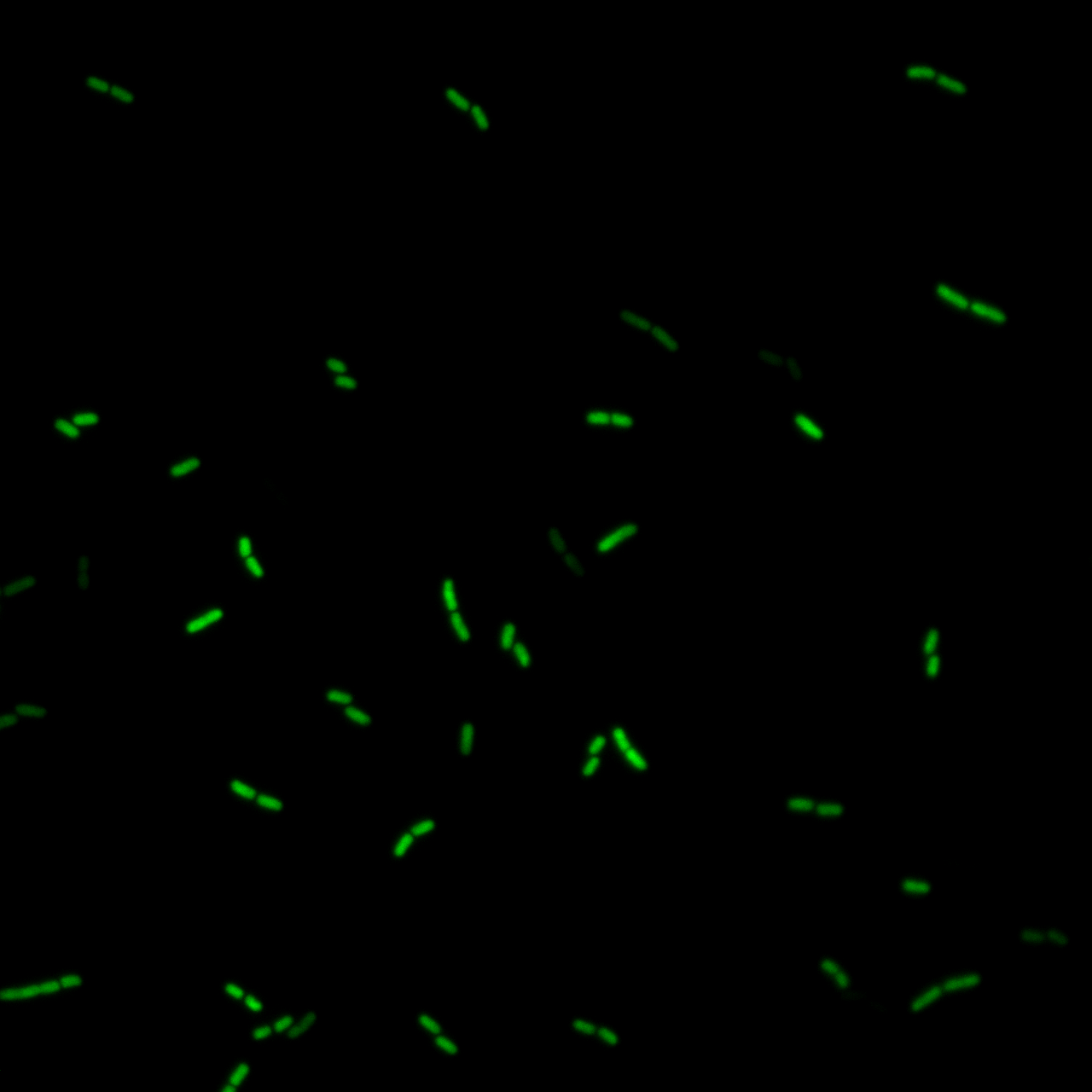

Supplement: Supplementary file 12 — Figure EV4 Source Data [file 44318_2025_595_MOESM12_ESM.zip › Fig. EV4/4E/4E_GFP_down.tif]

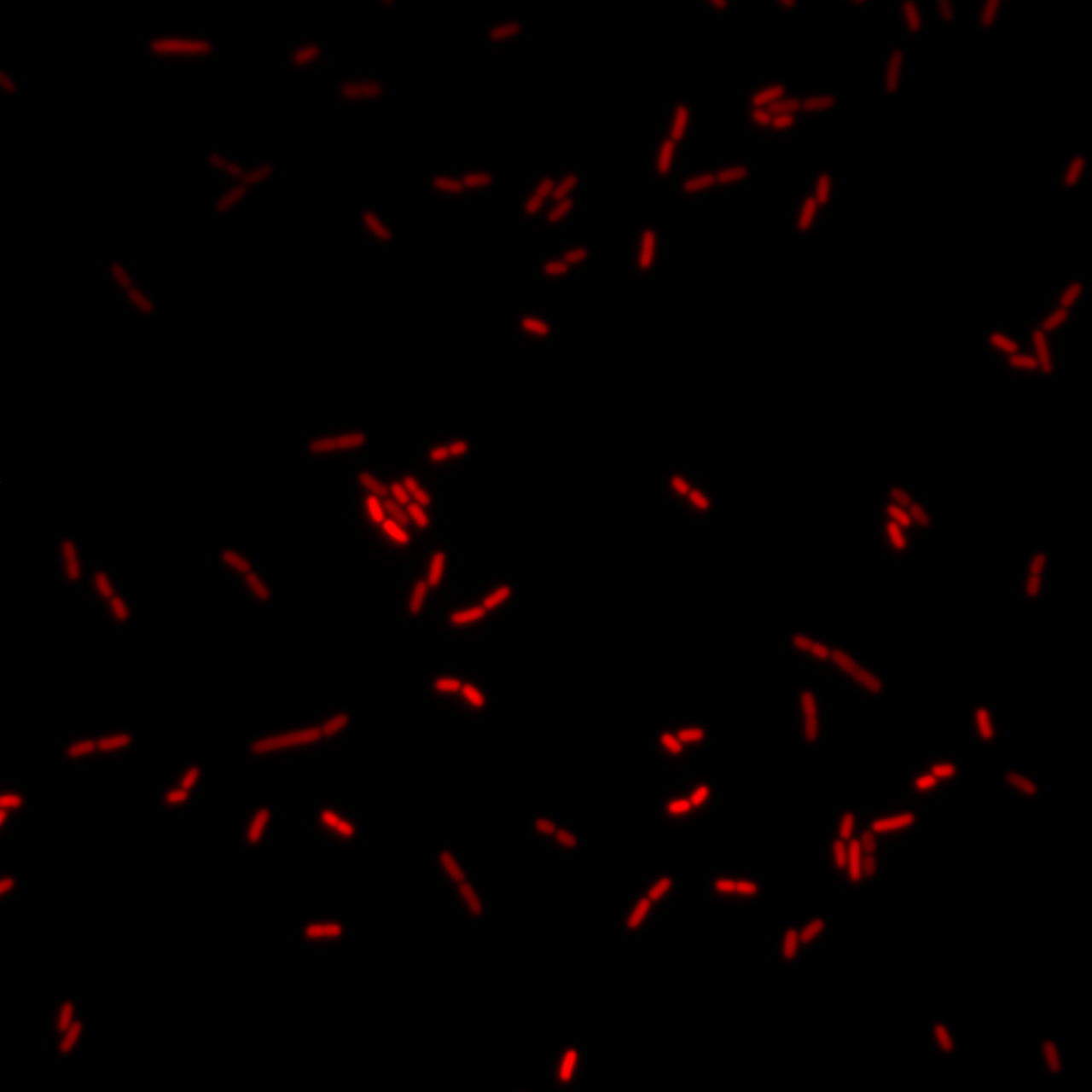

Supplement: Supplementary file 12 — Figure EV4 Source Data [file 44318_2025_595_MOESM12_ESM.zip › Fig. EV4/4E/4E_mCherry_up.tif]

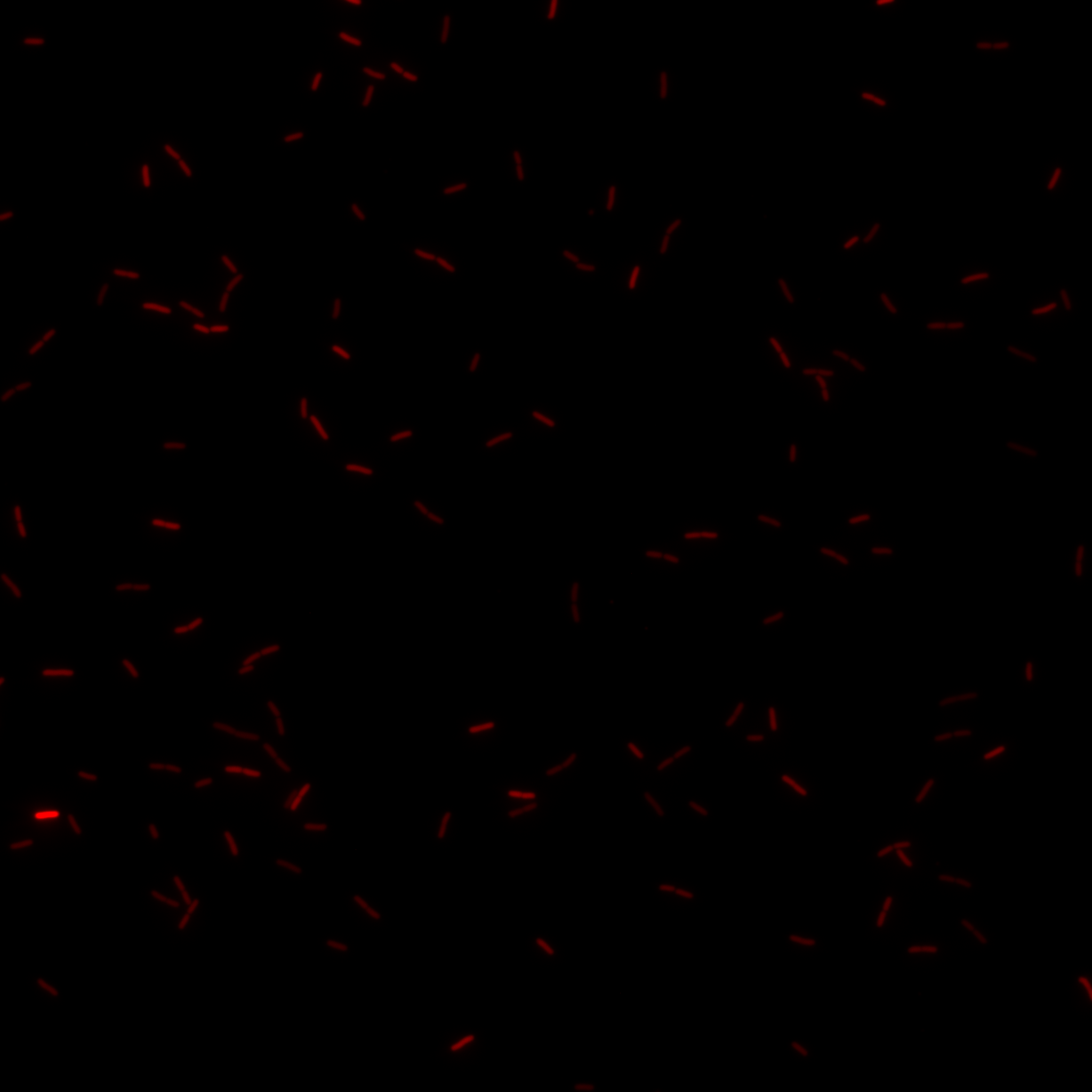

Supplement: Supplementary file 12 — Figure EV4 Source Data [file 44318_2025_595_MOESM12_ESM.zip › Fig. EV4/4B/4B_mCherry_up.tif]

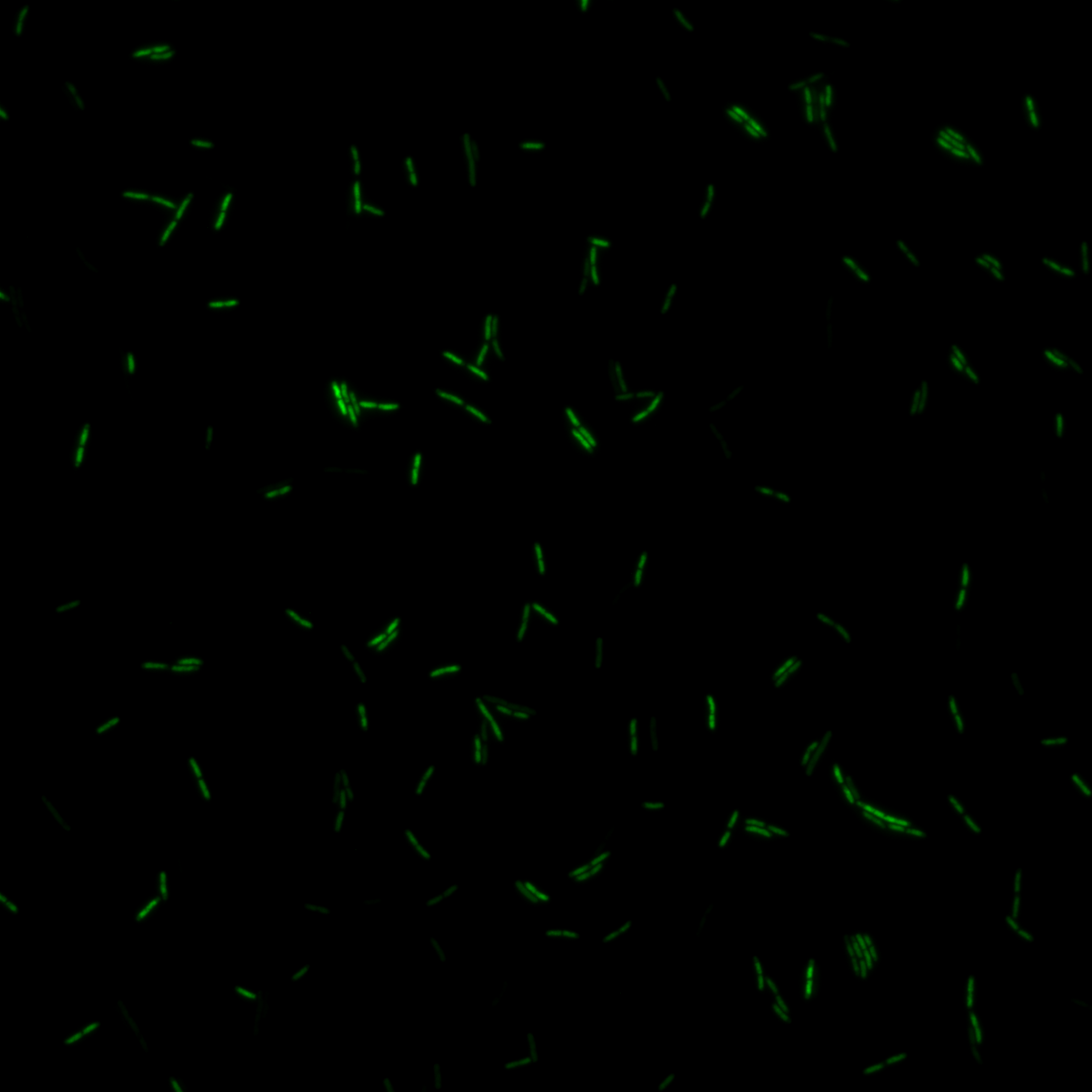

Supplement: Supplementary file 12 — Figure EV4 Source Data [file 44318_2025_595_MOESM12_ESM.zip › Fig. EV4/4B/4B_GFP_down.tif]

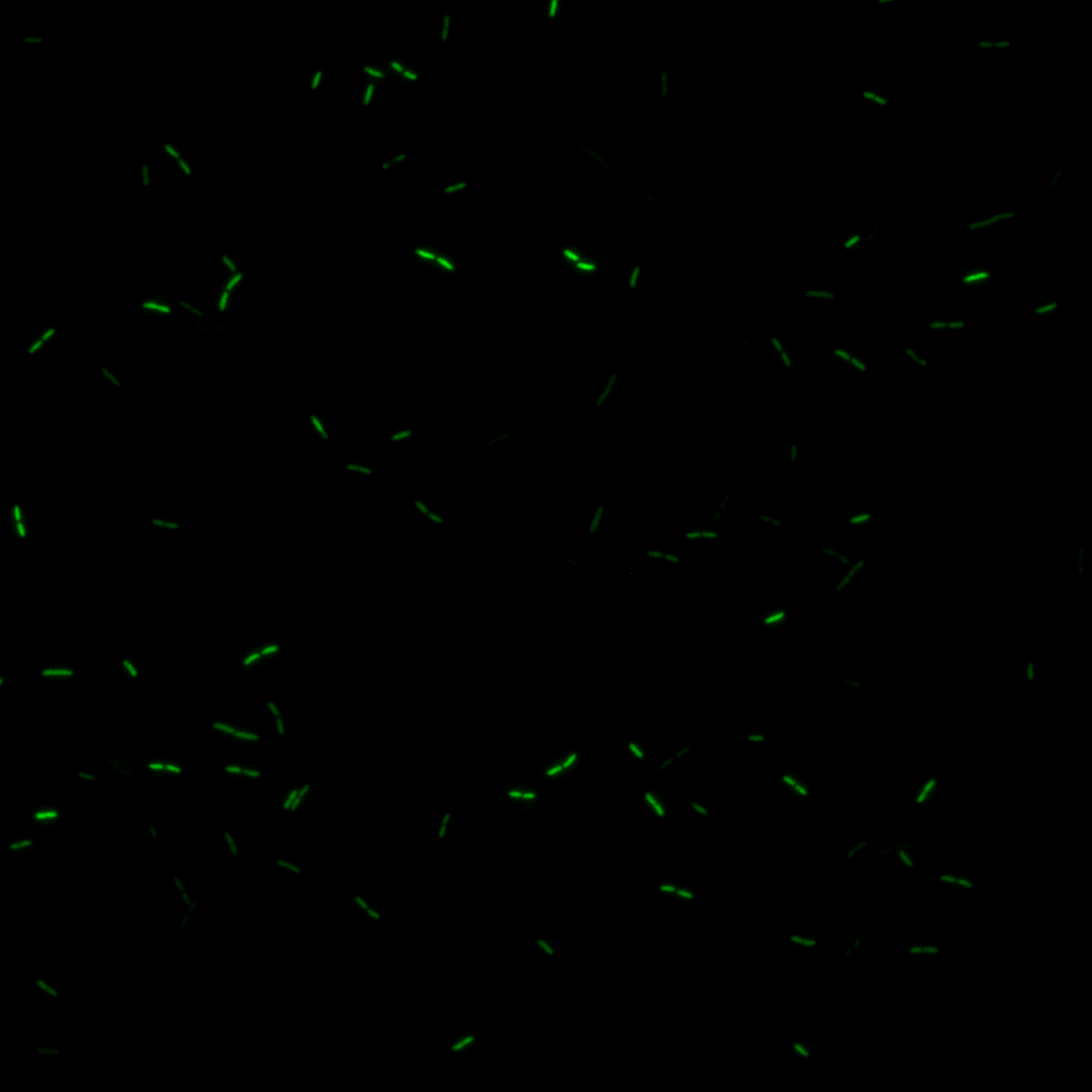

Supplement: Supplementary file 12 — Figure EV4 Source Data [file 44318_2025_595_MOESM12_ESM.zip › Fig. EV4/4B/4B_GFP_up.tif]

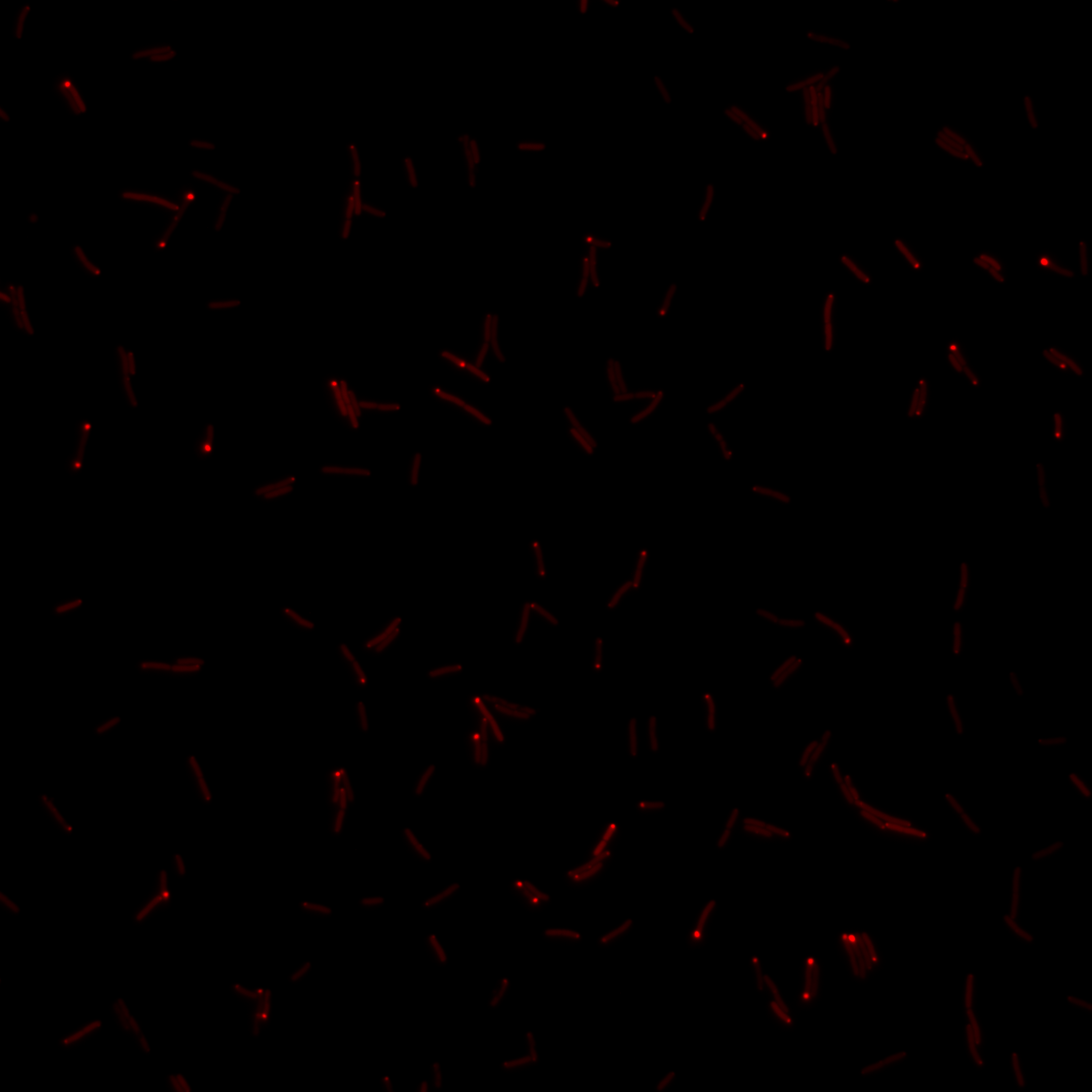

Supplement: Supplementary file 12 — Figure EV4 Source Data [file 44318_2025_595_MOESM12_ESM.zip › Fig. EV4/4B/4B_mCherry_down.tif]

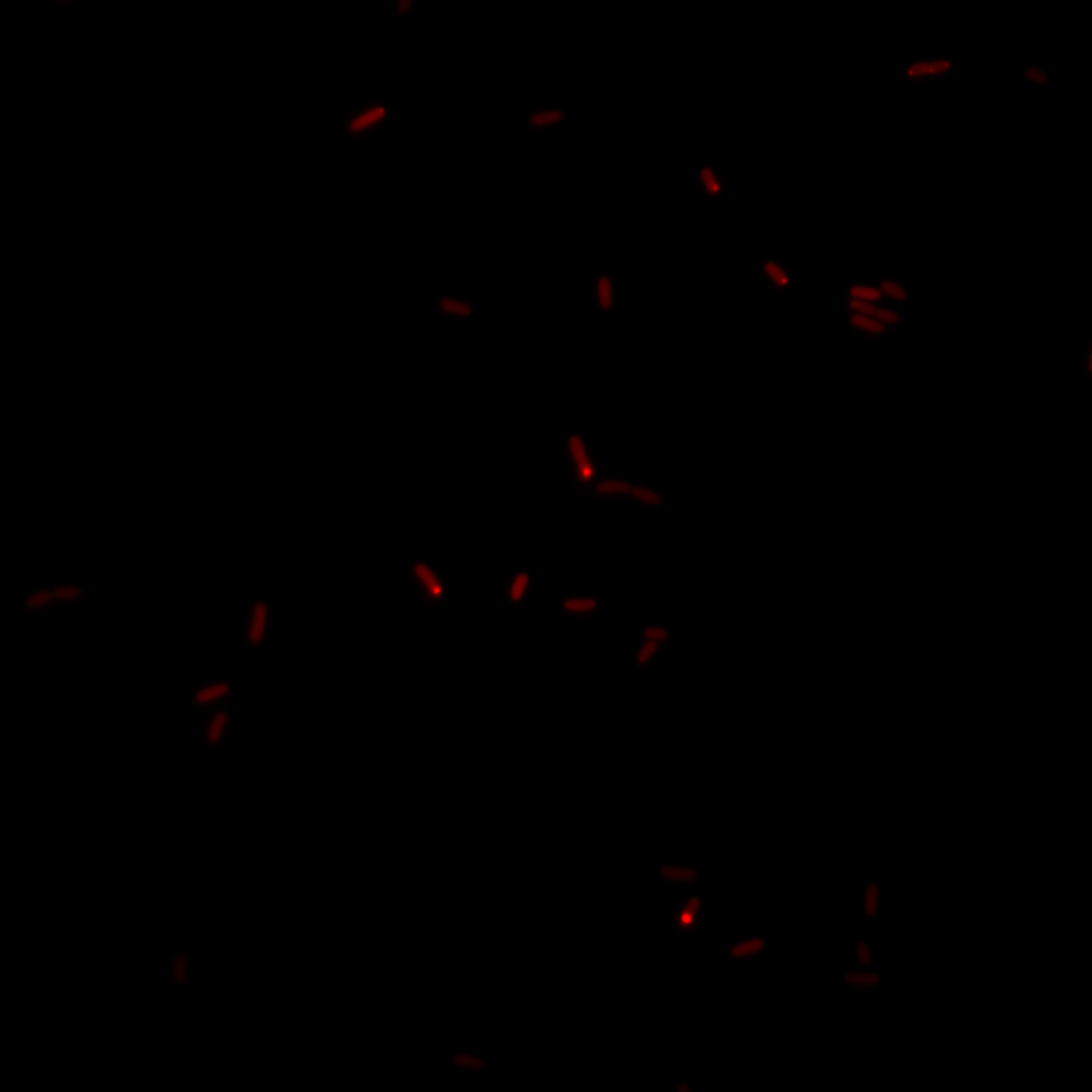

Supplement: Supplementary file 12 — Figure EV4 Source Data [file 44318_2025_595_MOESM12_ESM.zip › Fig. EV4/4D/4D_mCherry_down.tif]

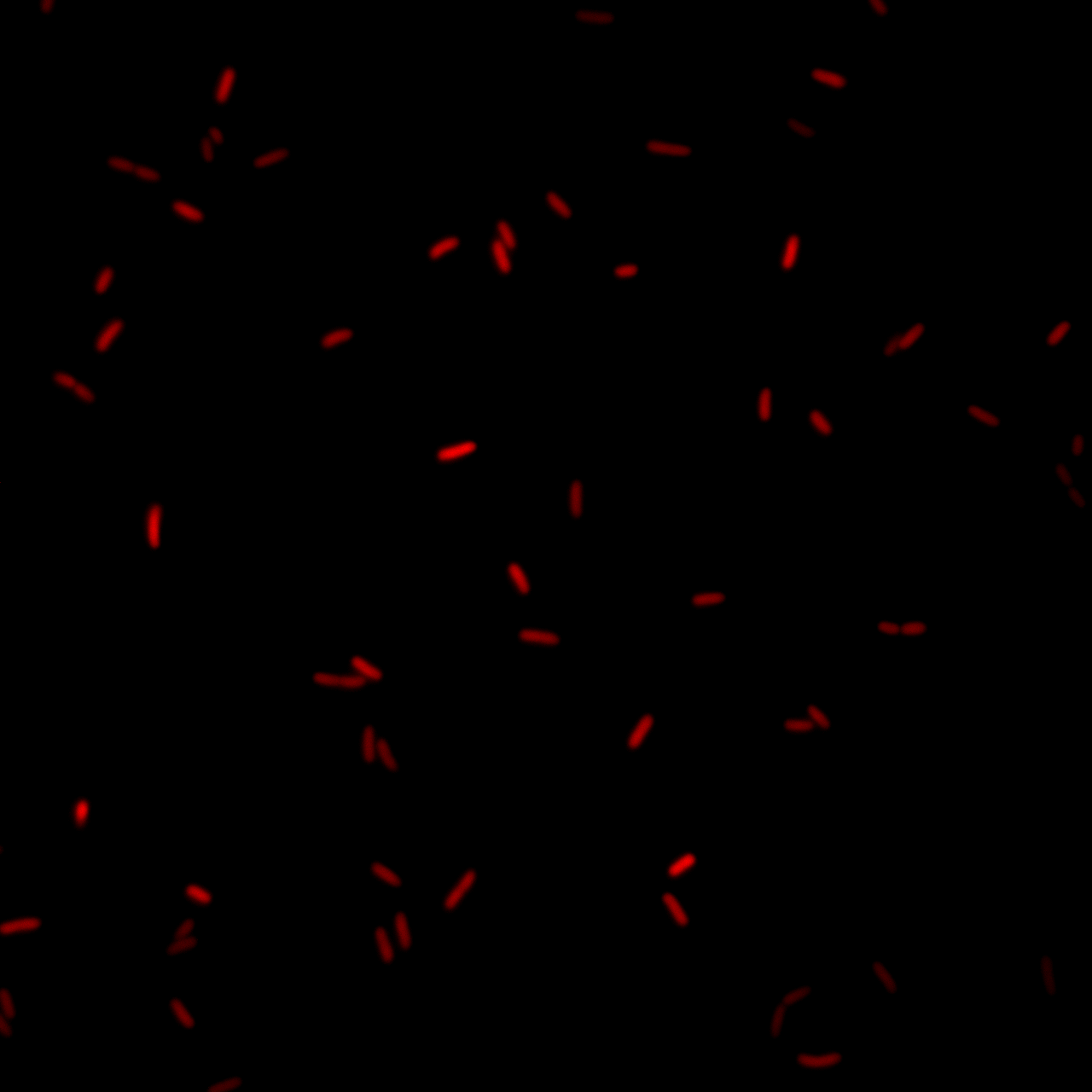

Supplement: Supplementary file 12 — Figure EV4 Source Data [file 44318_2025_595_MOESM12_ESM.zip › Fig. EV4/4D/4D_mCherry_up.tif]

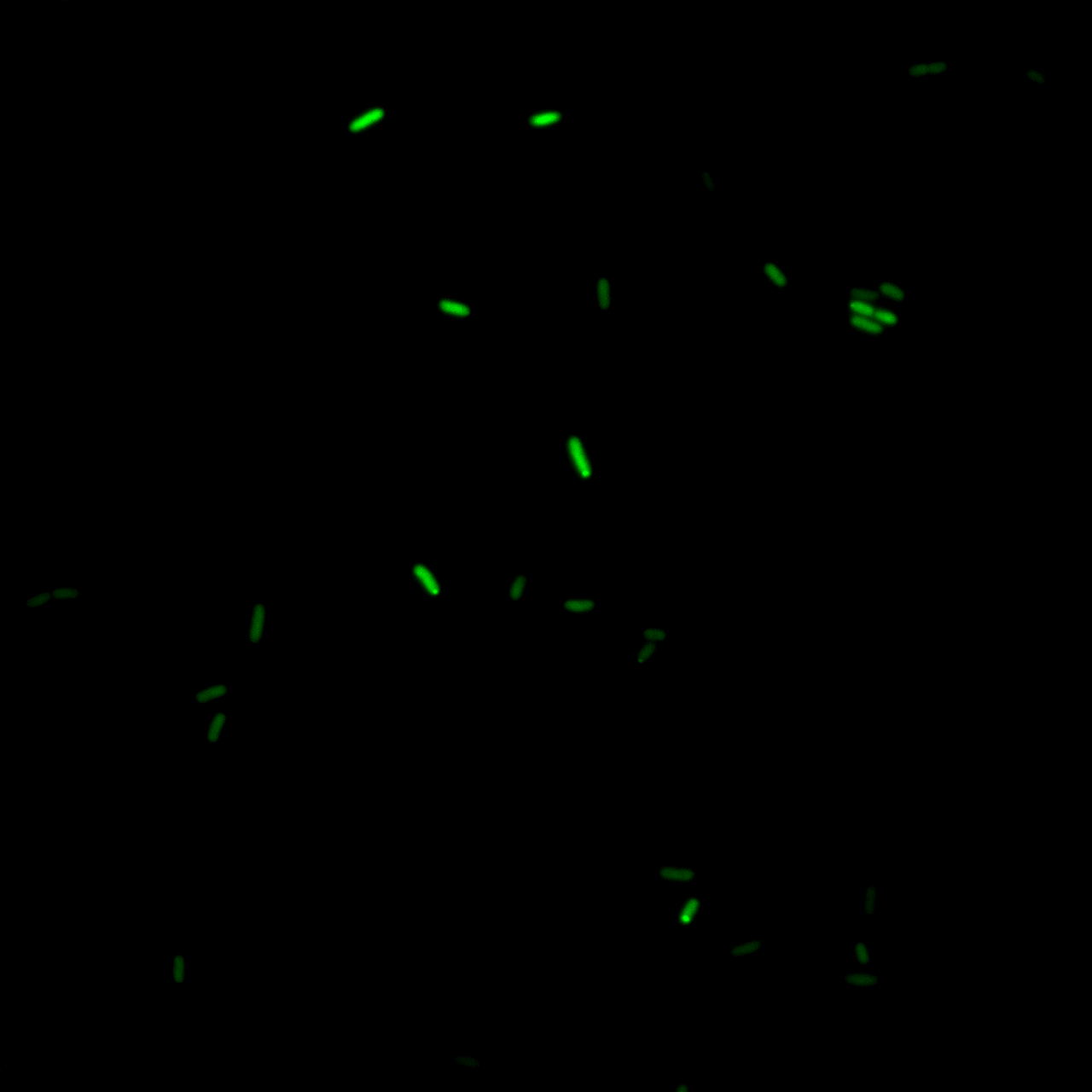

Supplement: Supplementary file 12 — Figure EV4 Source Data [file 44318_2025_595_MOESM12_ESM.zip › Fig. EV4/4D/4D_GFP_down.tif]

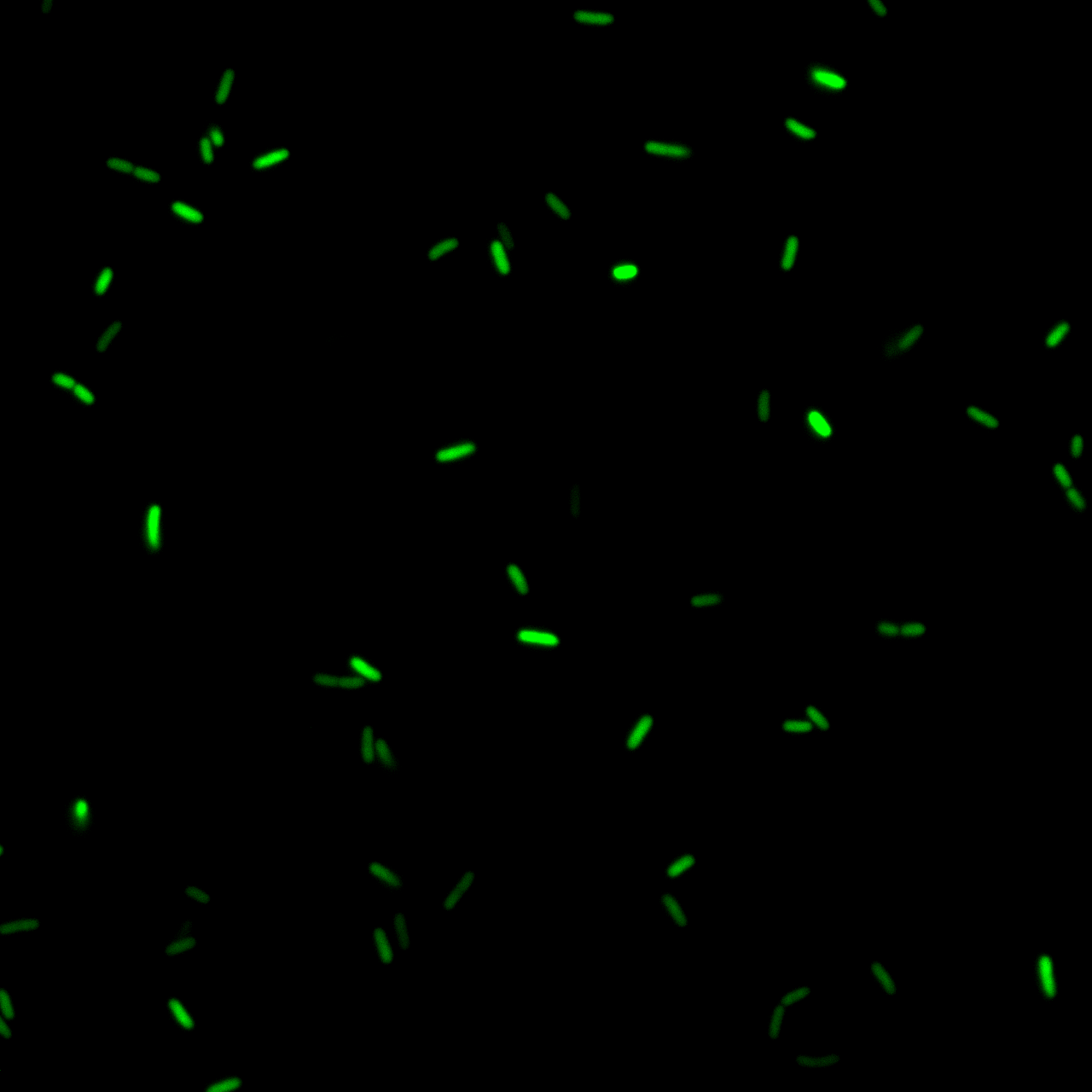

Supplement: Supplementary file 12 — Figure EV4 Source Data [file 44318_2025_595_MOESM12_ESM.zip › Fig. EV4/4D/4D_GFP_up.tif]

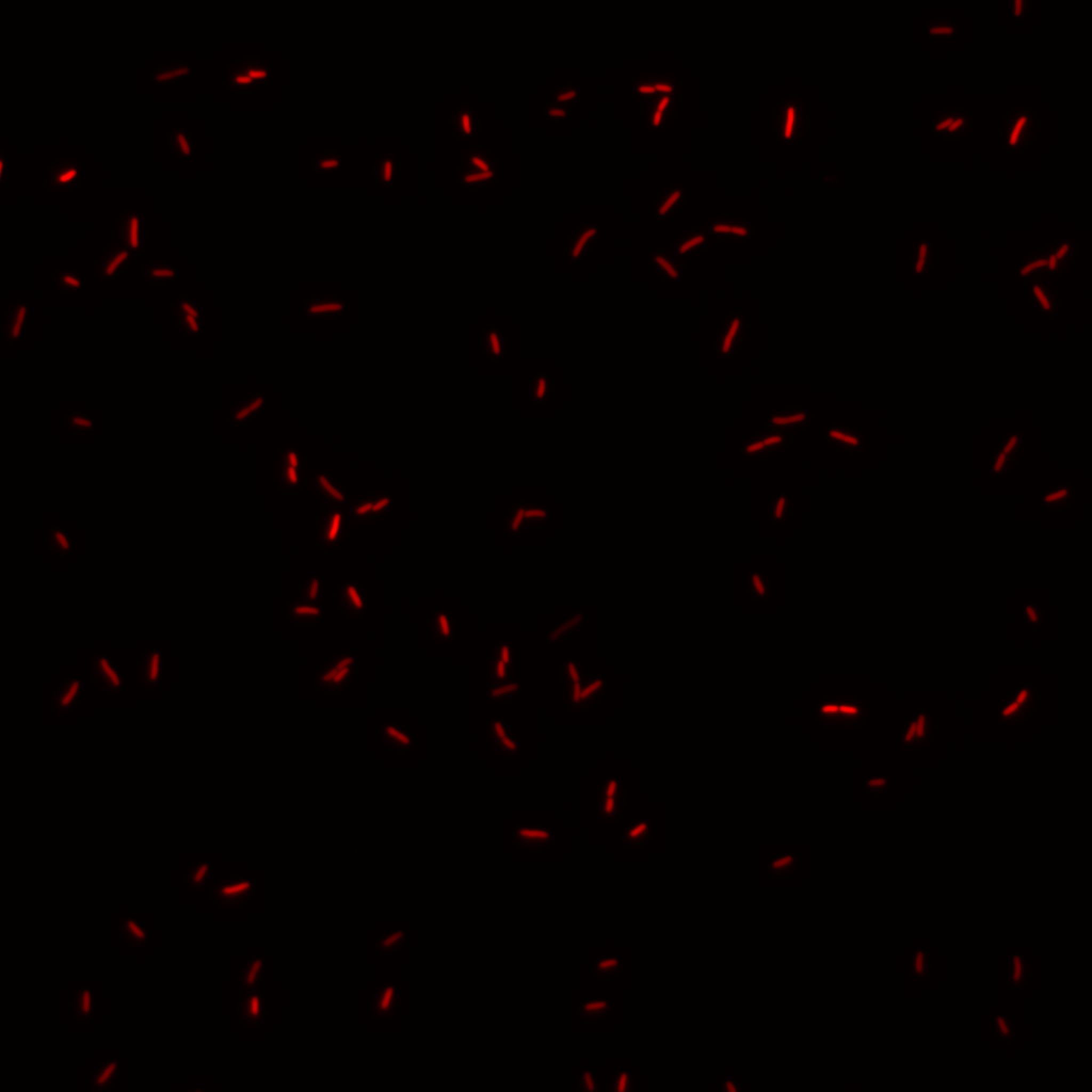

Supplement: Supplementary file 12 — Figure EV4 Source Data [file 44318_2025_595_MOESM12_ESM.zip › Fig. EV4/4A/4A_mCherry_up.tif]

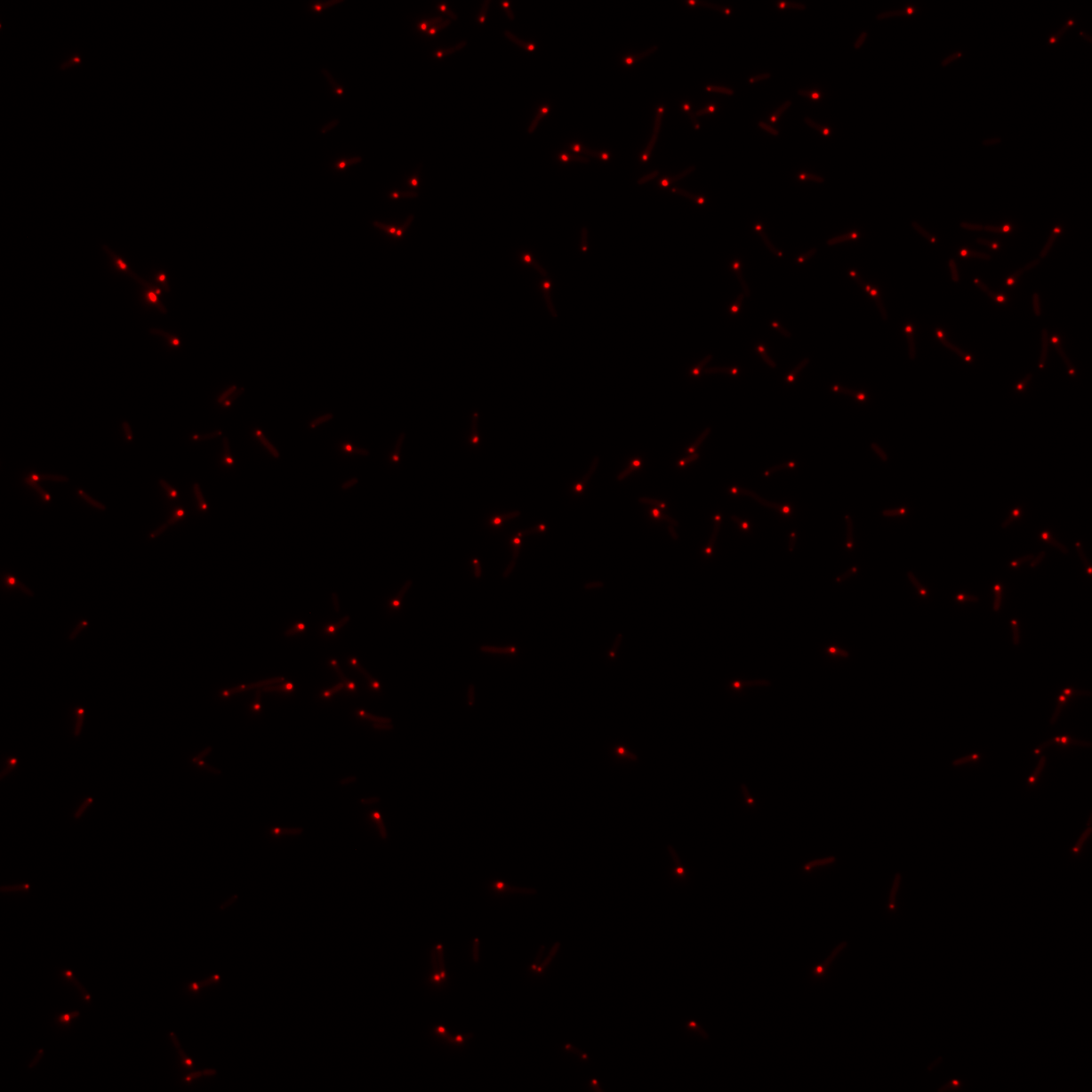

Supplement: Supplementary file 12 — Figure EV4 Source Data [file 44318_2025_595_MOESM12_ESM.zip › Fig. EV4/4A/4A_mCherry_down.tif]

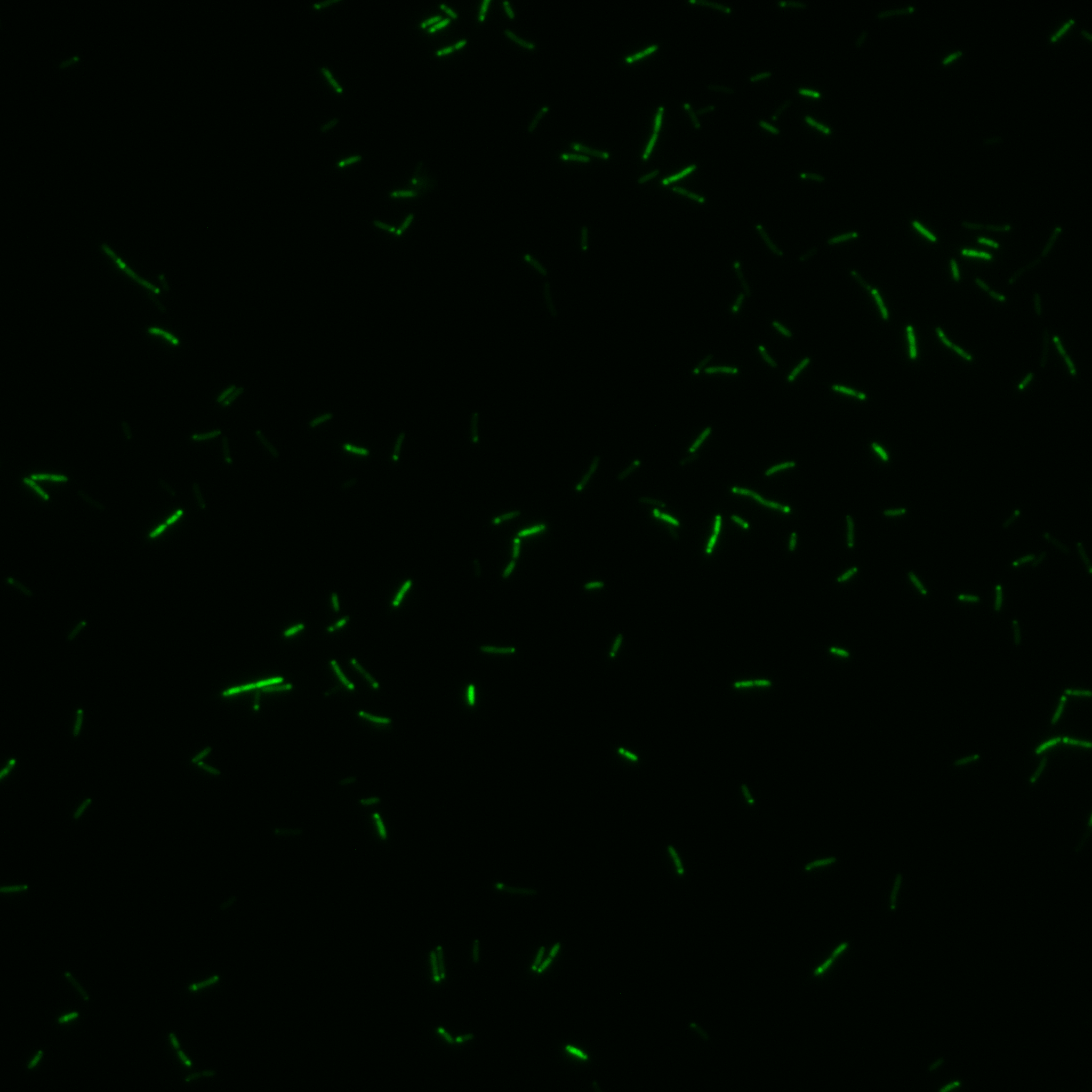

Supplement: Supplementary file 12 — Figure EV4 Source Data [file 44318_2025_595_MOESM12_ESM.zip › Fig. EV4/4A/4A_GFP_down.tif]

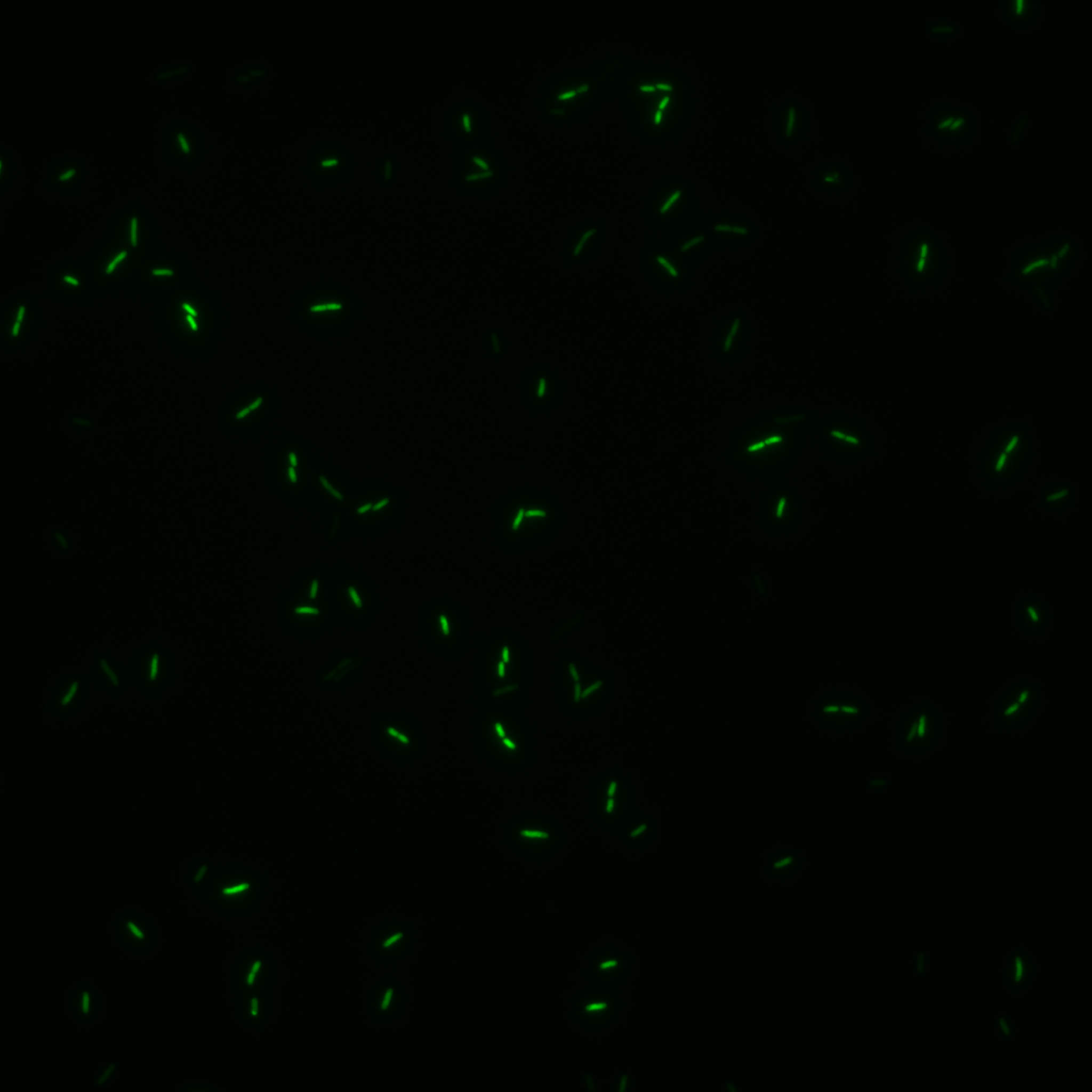

Supplement: Supplementary file 12 — Figure EV4 Source Data [file 44318_2025_595_MOESM12_ESM.zip › Fig. EV4/4A/4A_GFP_up.tif]

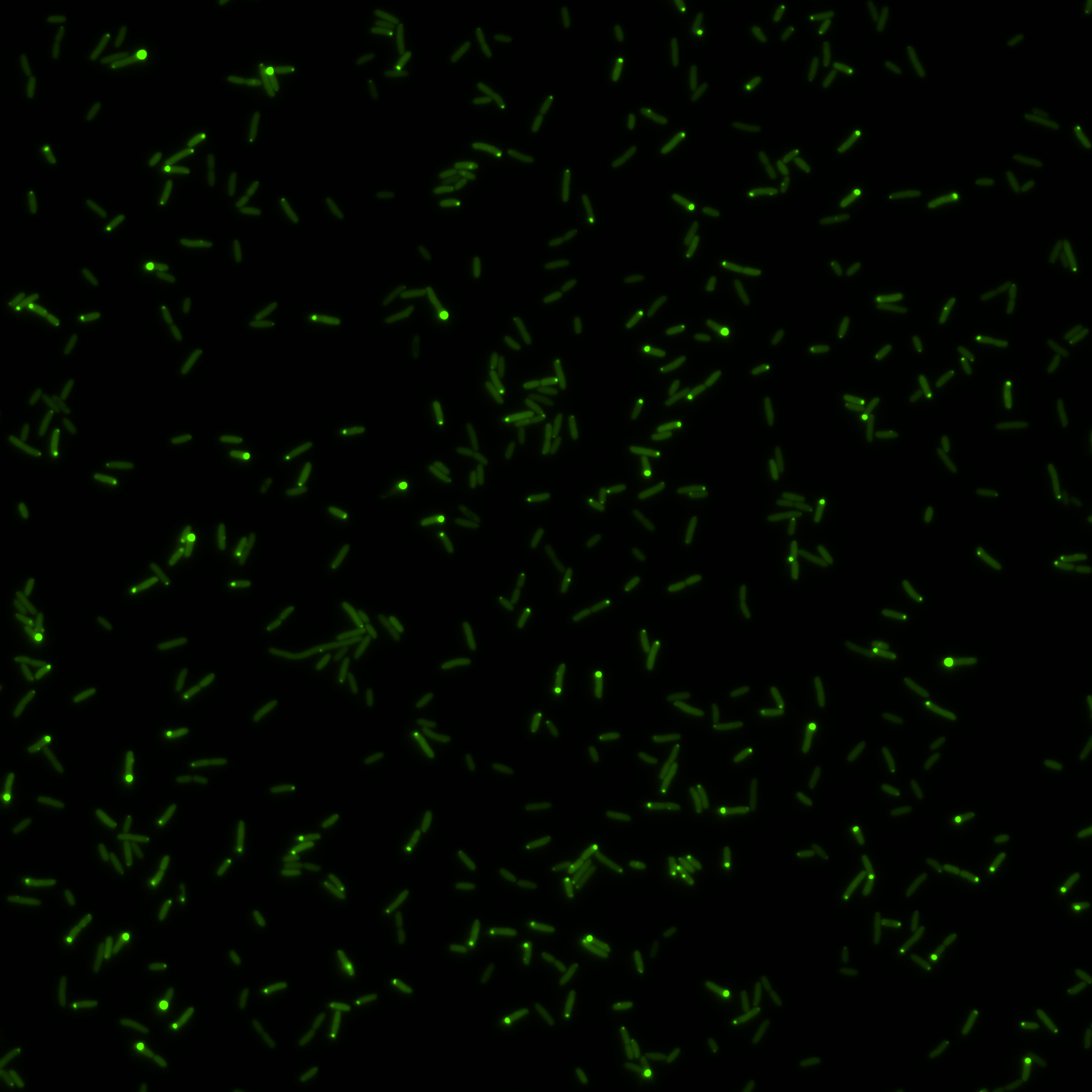

Supplement: Supplementary file 14 — Appendix Figure Source Data [file 44318_2025_595_MOESM14_ESM.zip › Appendix figure/Appendix Fig. S7/7D/7D10.tif]

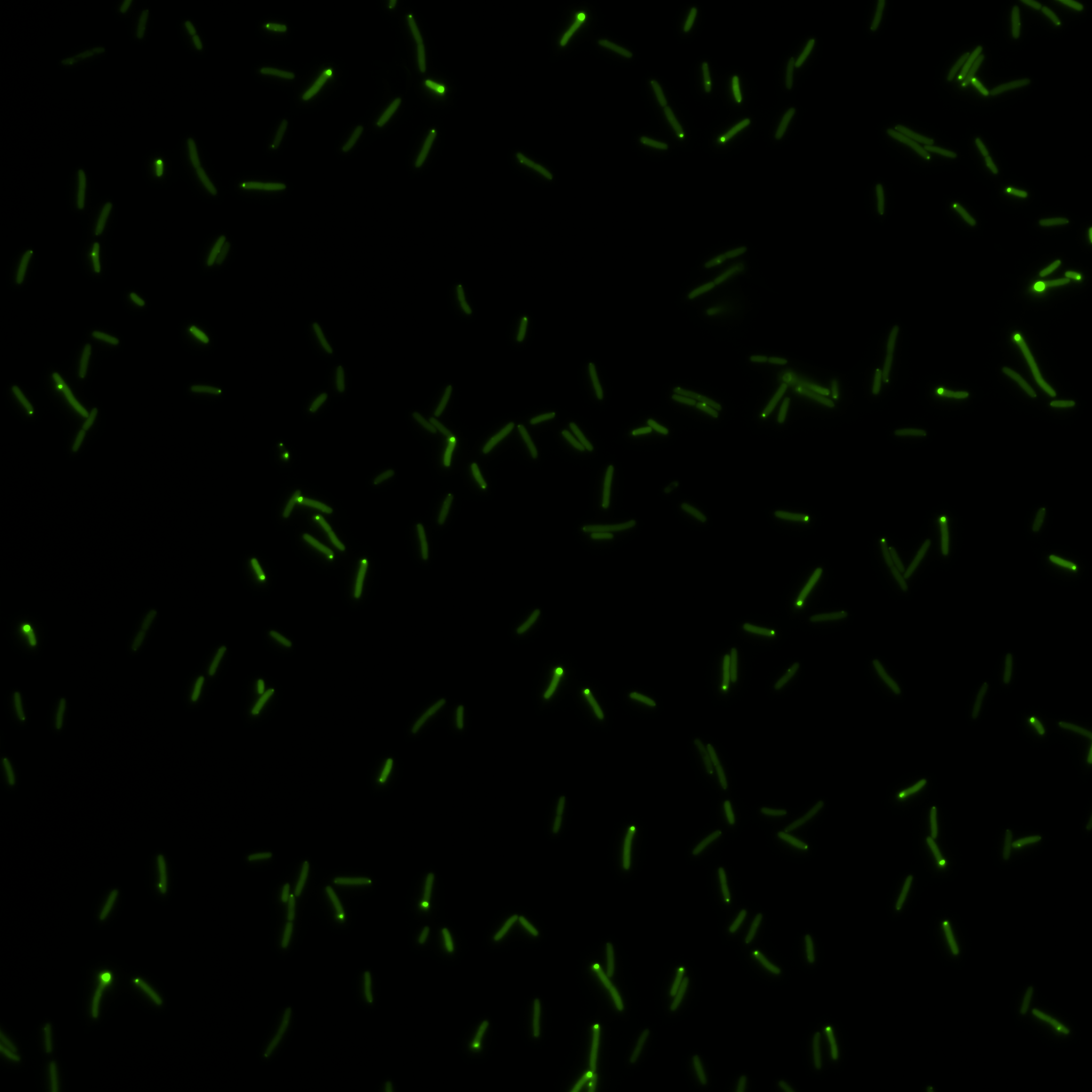

Supplement: Supplementary file 14 — Appendix Figure Source Data [file 44318_2025_595_MOESM14_ESM.zip › Appendix figure/Appendix Fig. S7/7D/7D30.tif]

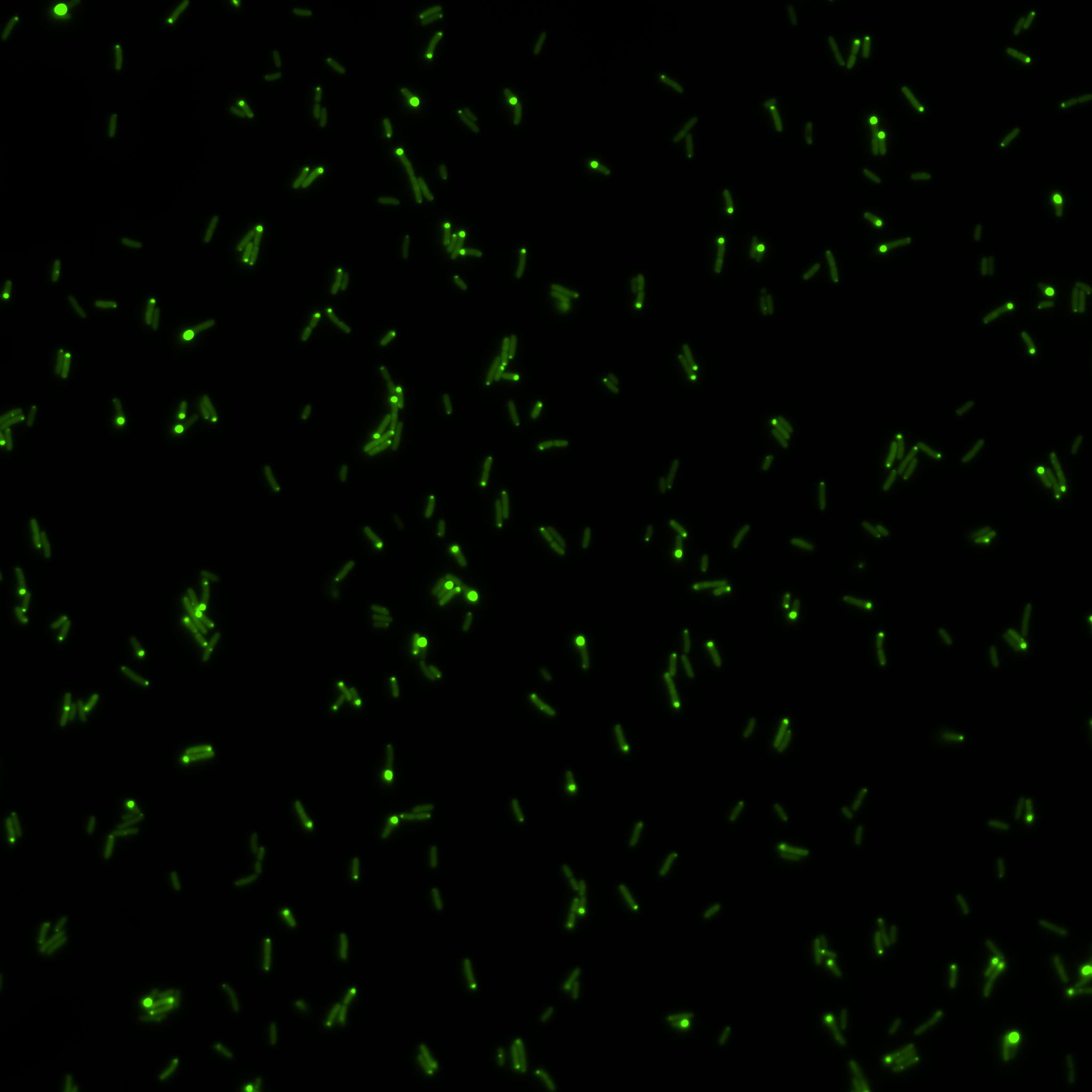

Supplement: Supplementary file 14 — Appendix Figure Source Data [file 44318_2025_595_MOESM14_ESM.zip › Appendix figure/Appendix Fig. S7/7D/7D0.tif]

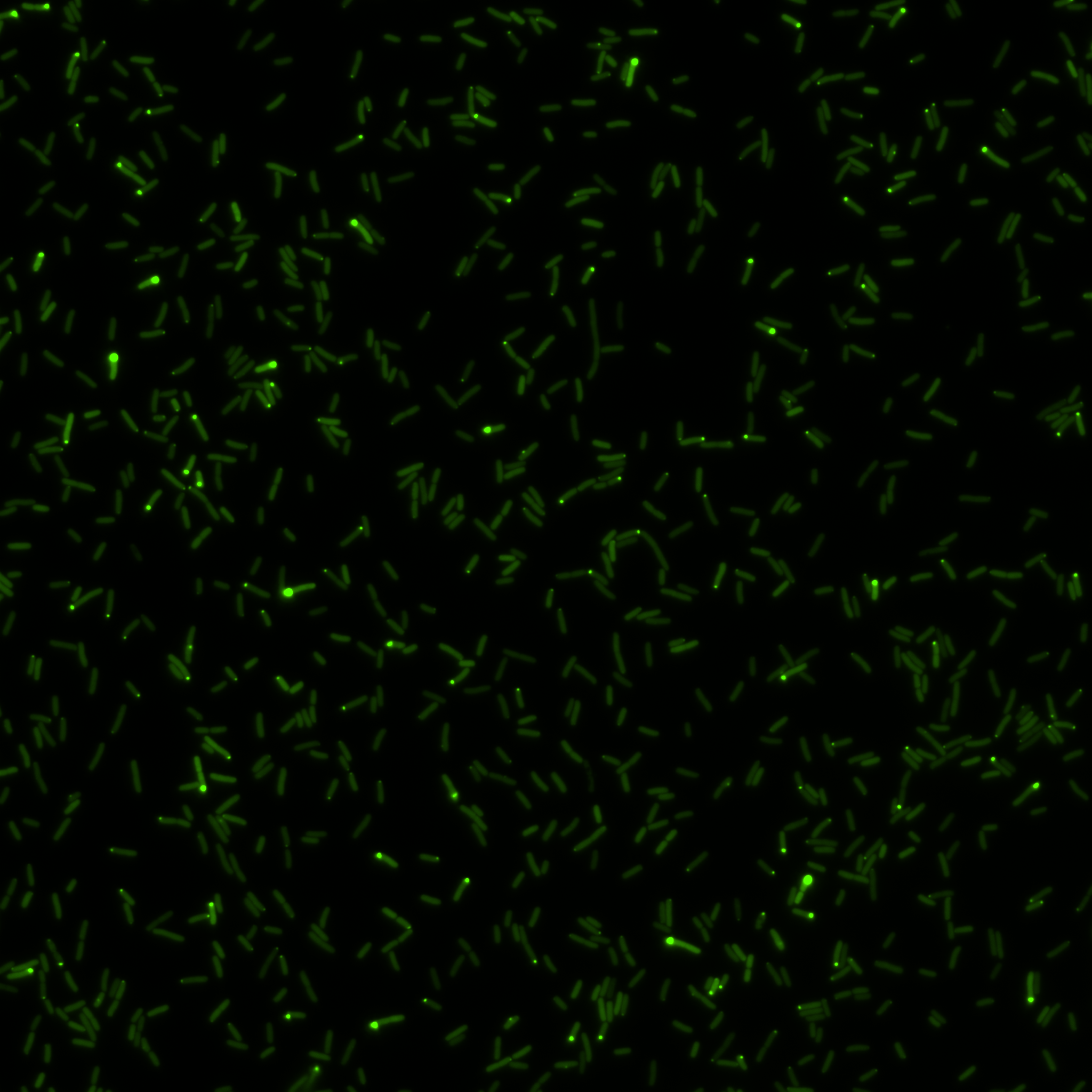

Supplement: Supplementary file 14 — Appendix Figure Source Data [file 44318_2025_595_MOESM14_ESM.zip › Appendix figure/Appendix Fig. S7/7D/7D90.tif]

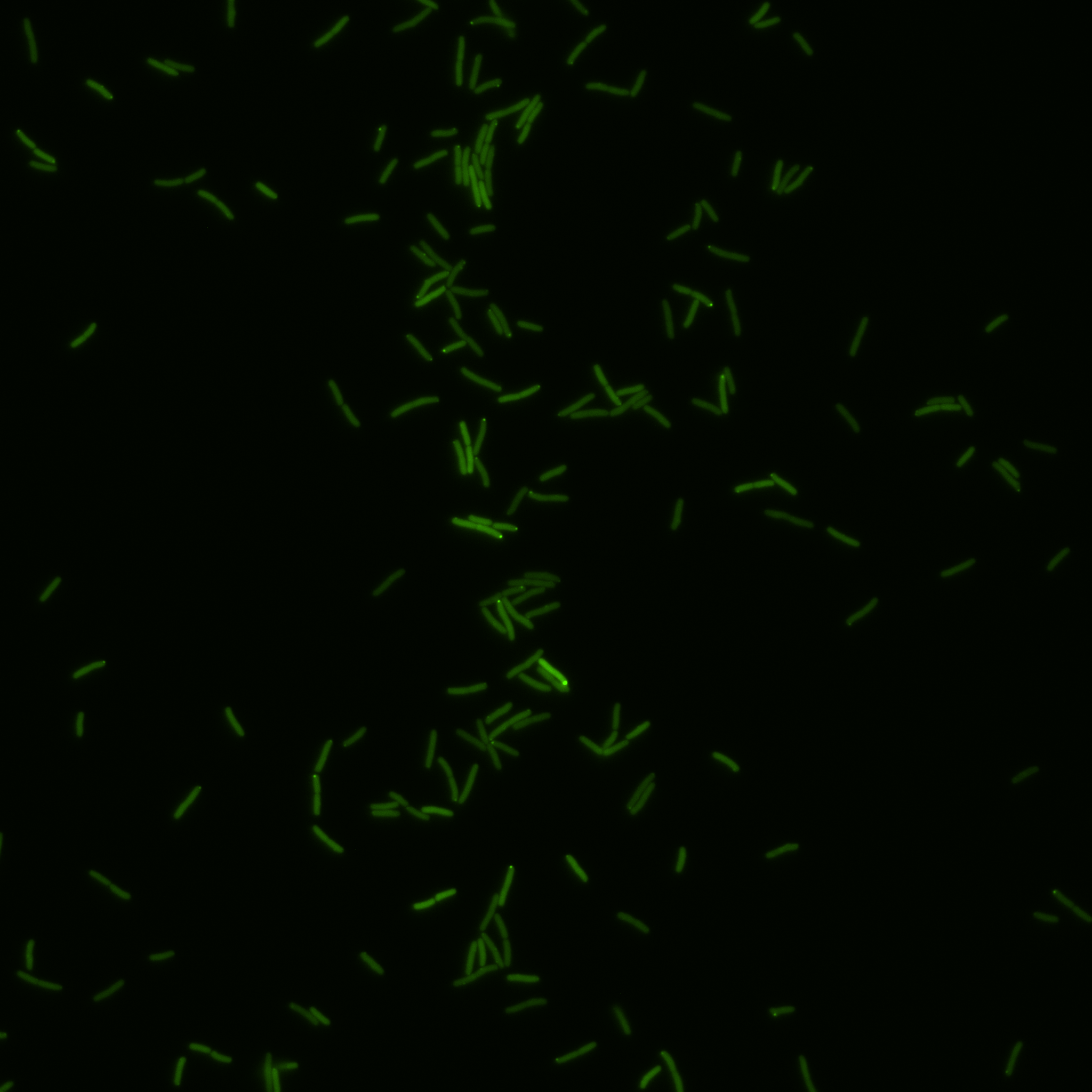

Supplement: Supplementary file 14 — Appendix Figure Source Data [file 44318_2025_595_MOESM14_ESM.zip › Appendix figure/Appendix Fig. S7/7C/7C.tif]

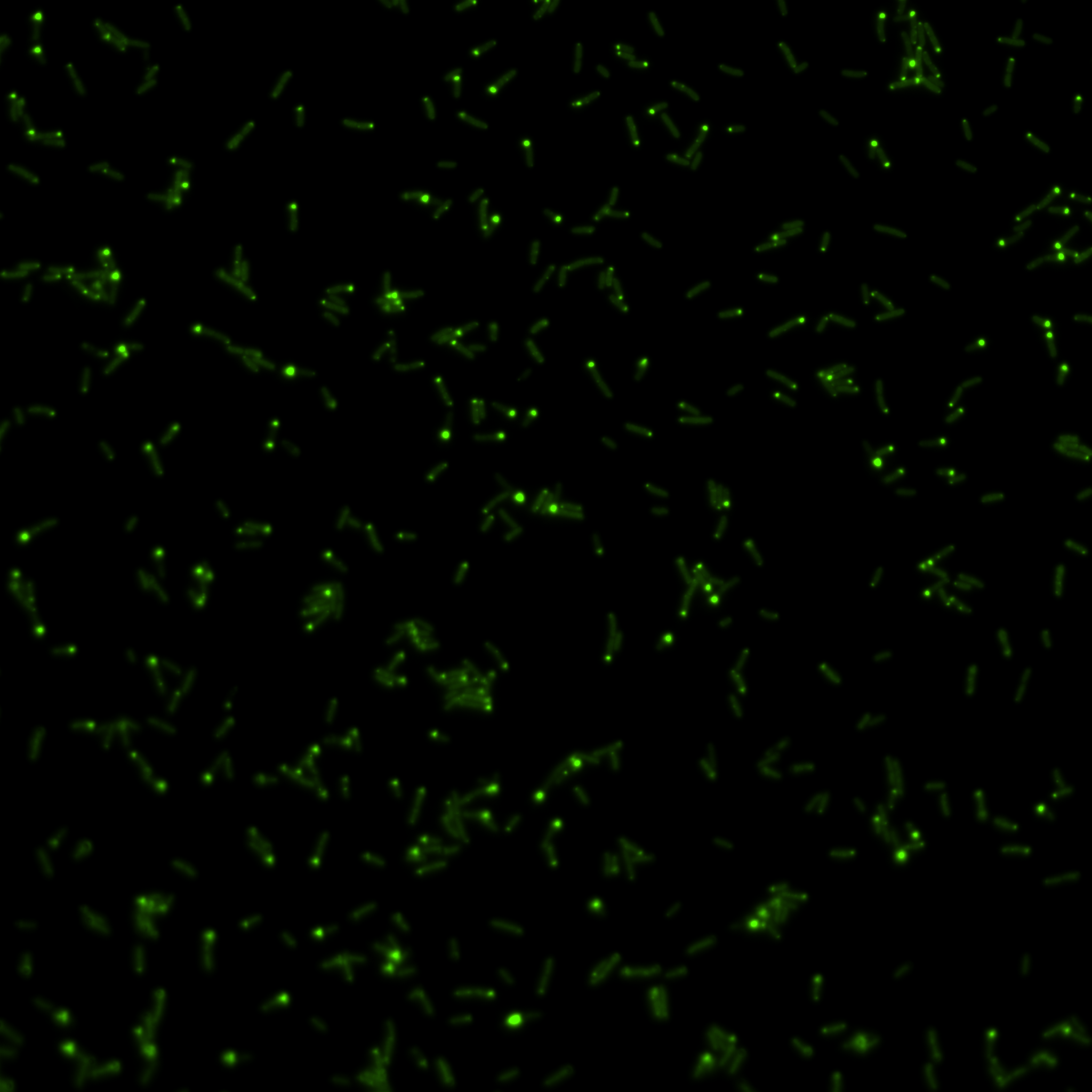

Supplement: Supplementary file 14 — Appendix Figure Source Data [file 44318_2025_595_MOESM14_ESM.zip › Appendix figure/Appendix Fig. S7/7B/7B.tif]

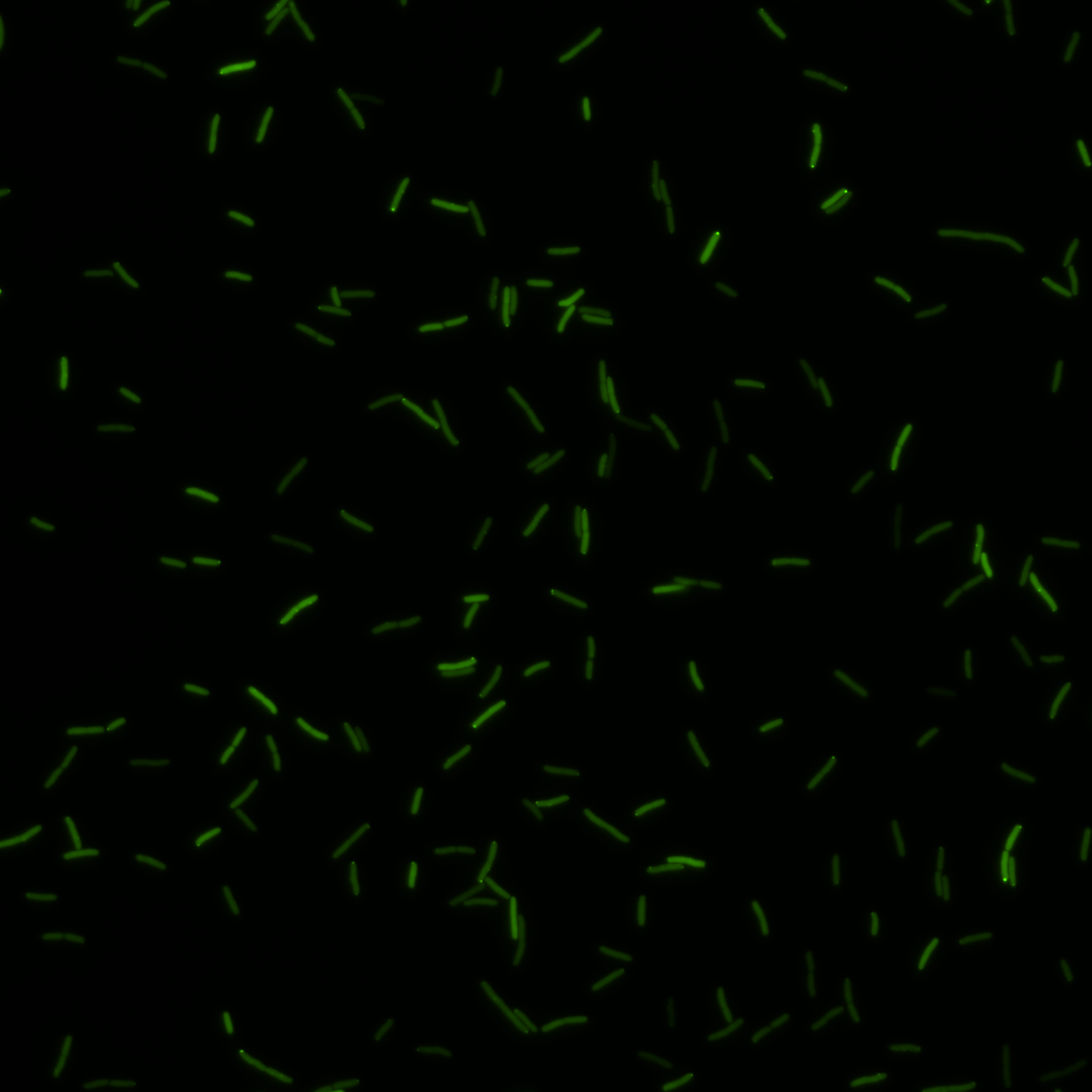

Supplement: Supplementary file 14 — Appendix Figure Source Data [file 44318_2025_595_MOESM14_ESM.zip › Appendix figure/Appendix Fig. S7/7E/7E10.tif]

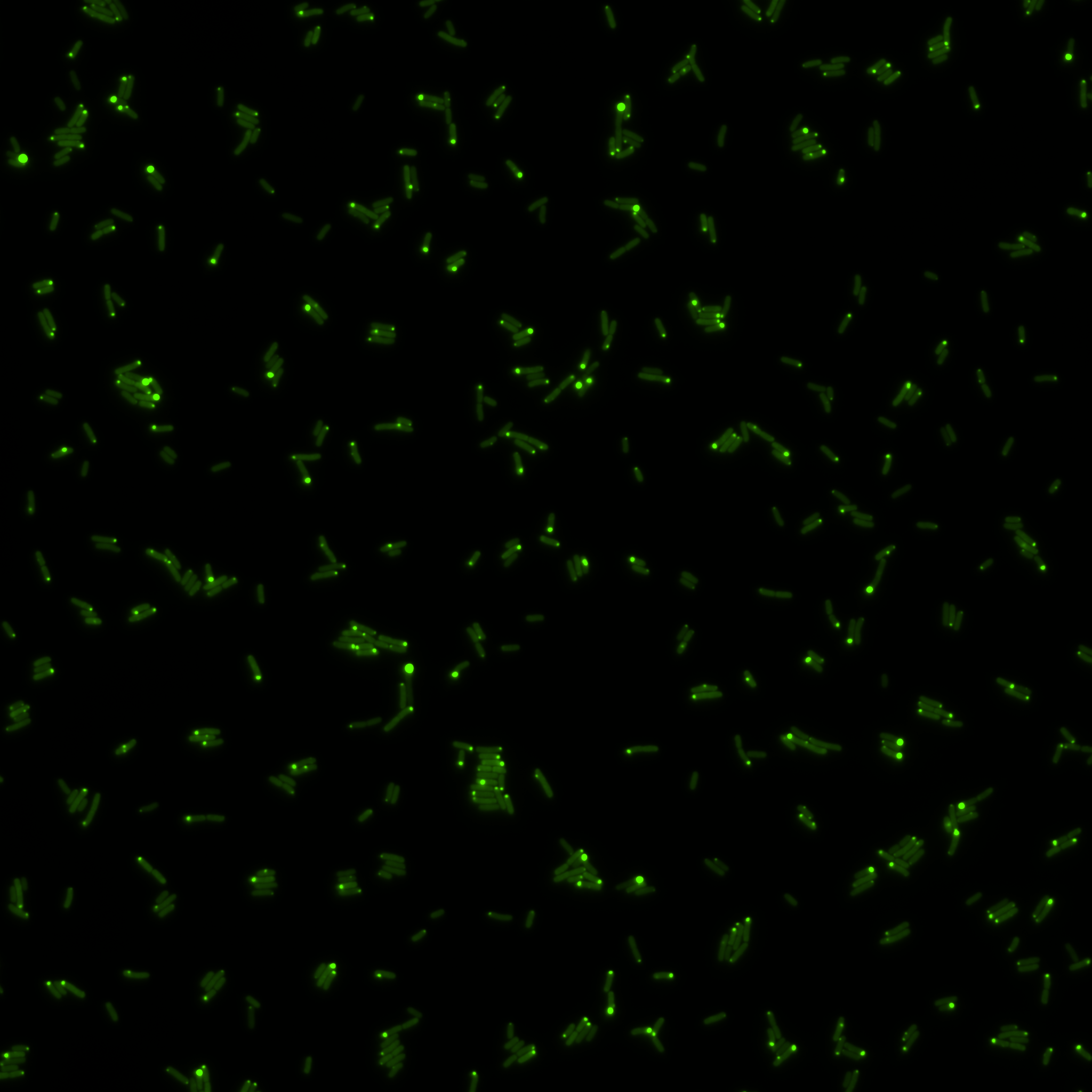

Supplement: Supplementary file 14 — Appendix Figure Source Data [file 44318_2025_595_MOESM14_ESM.zip › Appendix figure/Appendix Fig. S7/7E/7E90.tif]

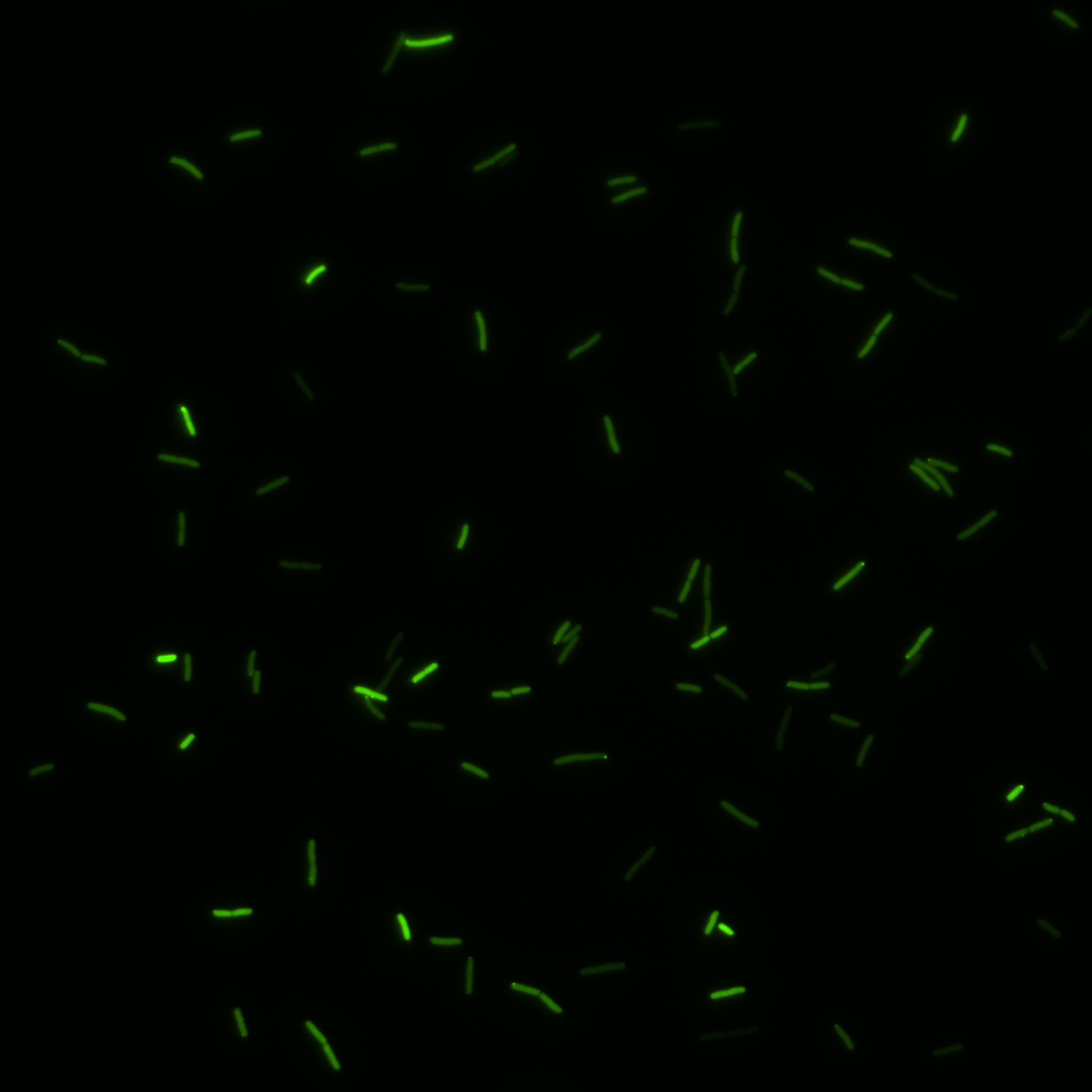

Supplement: Supplementary file 14 — Appendix Figure Source Data [file 44318_2025_595_MOESM14_ESM.zip › Appendix figure/Appendix Fig. S7/7E/7E0.tif]

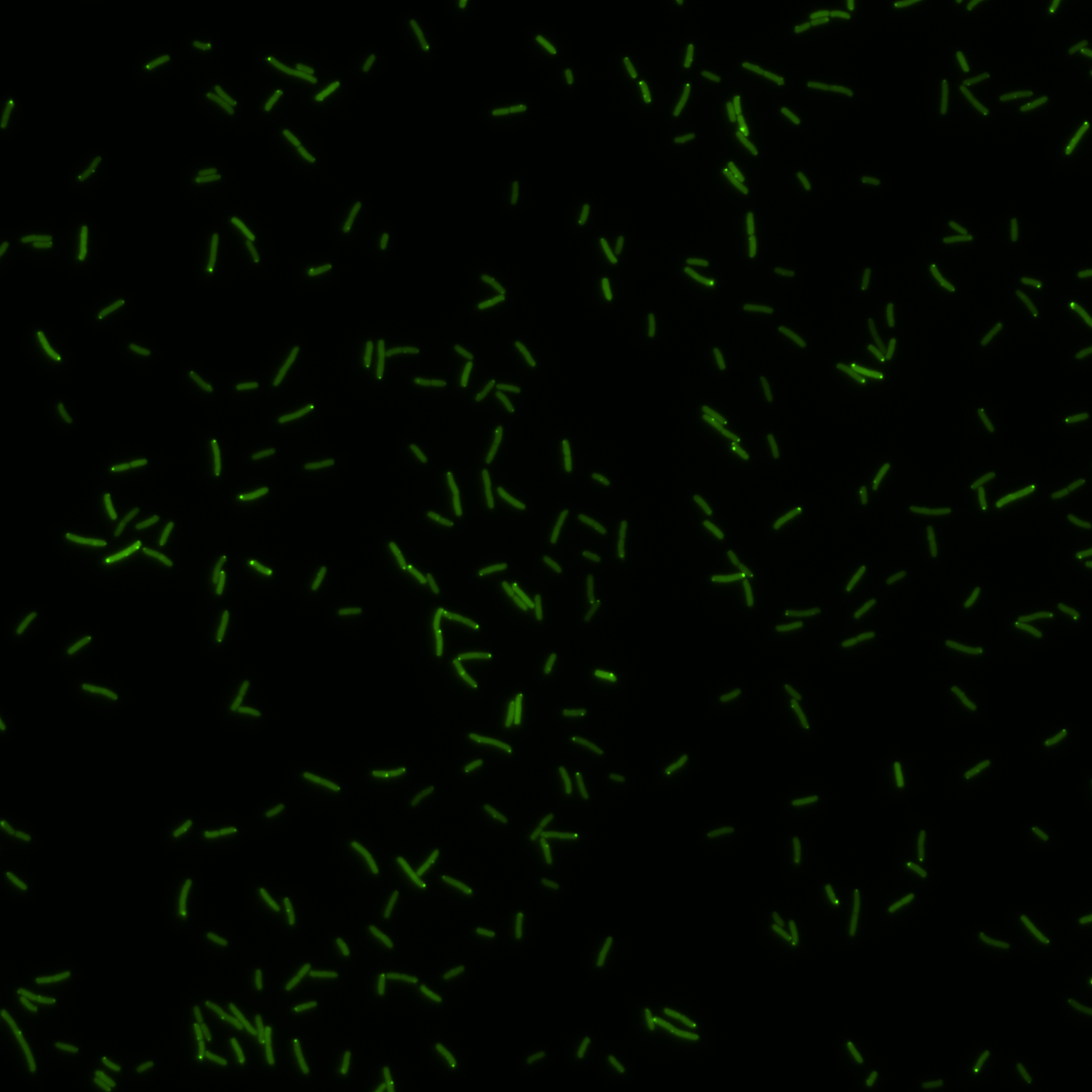

Supplement: Supplementary file 14 — Appendix Figure Source Data [file 44318_2025_595_MOESM14_ESM.zip › Appendix figure/Appendix Fig. S7/7E/7E30.tif]

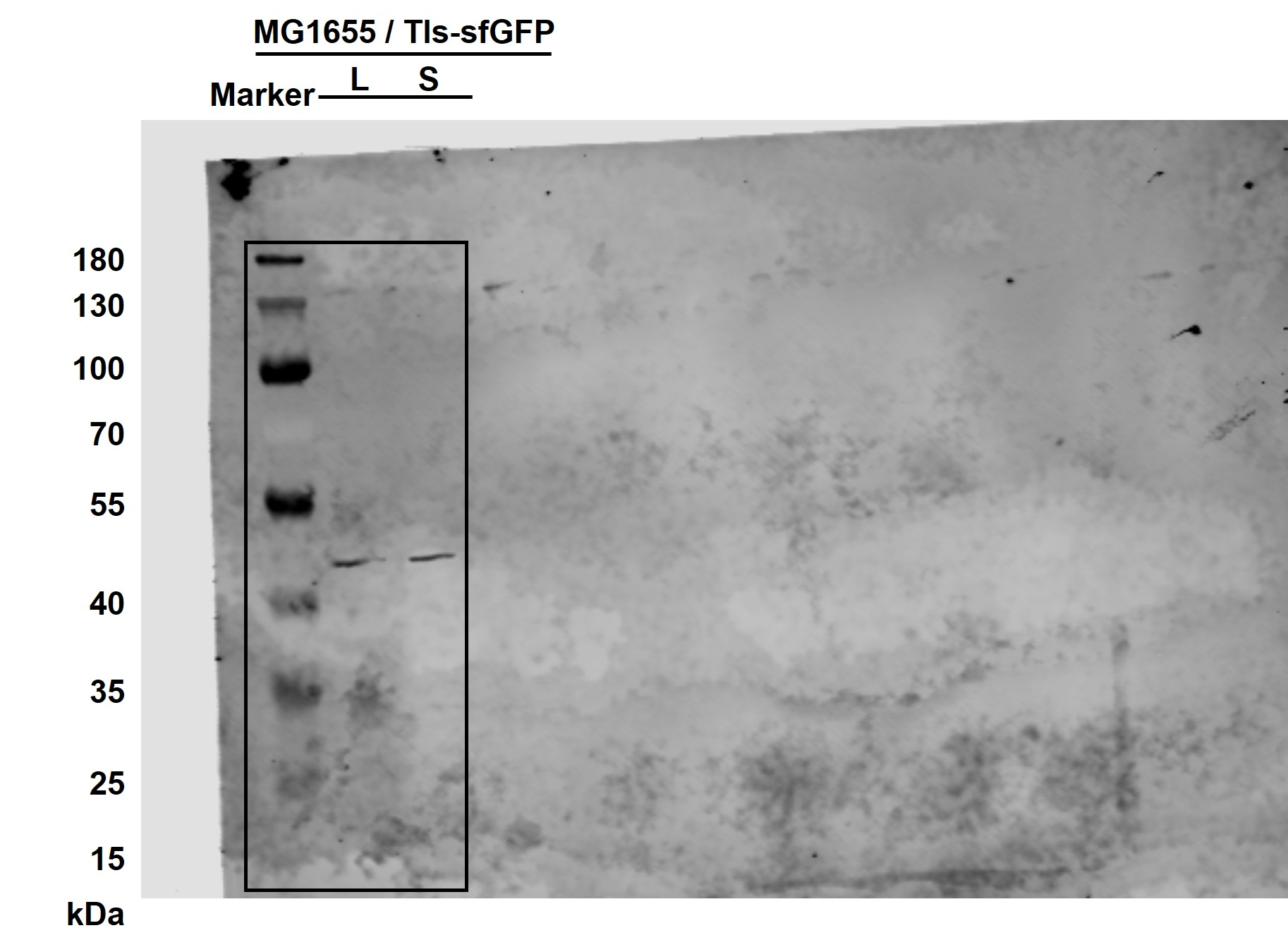

Supplement: Supplementary file 14 — Appendix Figure Source Data [file 44318_2025_595_MOESM14_ESM.zip › Appendix figure/Appendix Fig. S7/7A/7A.tif]

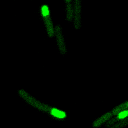

Supplement: Supplementary file 14 — Appendix Figure Source Data [file 44318_2025_595_MOESM14_ESM.zip › Appendix figure/Appendix Fig. S9/9A/Pre.tif]

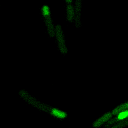

Supplement: Supplementary file 14 — Appendix Figure Source Data [file 44318_2025_595_MOESM14_ESM.zip › Appendix figure/Appendix Fig. S9/9A/52.8.tif]

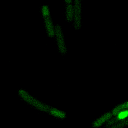

Supplement: Supplementary file 14 — Appendix Figure Source Data [file 44318_2025_595_MOESM14_ESM.zip › Appendix figure/Appendix Fig. S9/9A/14.2.tif]
