# Supplementary material for: Control of flagellar gene expression by a chemotaxis receptor-like regulator in pathogenic Escherichia coli
Source: EMBO J. 2025 Oct 13;44(22):6675–703. doi: 10.1038/s44318-025-00595-x (PMC12623472; doi:10.1038/s44318-025-00595-x)
Supplement: Supplementary file 15 — Expanded View Figures [file 44318_2025_595_MOESM15_ESM.pdf]

Expanded View Figures

Figure EV1. Effect of TIs on flagellar gene expression and motility in *E. coli* MG1655.

(**A**) Fluorescence levels of P*fliC-gfp* normalized by OD_{600} values in MG1655 carrying either empty vector pTrc99A or pTrc99A-TIs induced with indicated concentrations of IPTG. Measurements were performed in the log phase of growth in a plate reader. Promoter region of *gadX*, RpoS-dependent gene unrelated to motility, was used as a control. Values represent the means and standard deviations of a minimum of three biological replicates, in each case normalized to the reporter activity in the reference strain (MG1655/pTrc99A). Statistical significance was determined using unpaired two-tailed Student's *t* test. The *P* values are denoted as ns ($P > 0.05$), * ($P < 0.05$), ** ($P < 0.005$), *** ($P < 0.001$). (*P* values from left to right: ***$P = 8.88E\text{-}06$, ***$P = 3.35E\text{-}09$, ***$P = 1.59E\text{-}06$, ***$P = 3.60E\text{-}09$, ***$P = 2.52E\text{-}11$, ***$P = 4.71E\text{-}12$, ns = 0.0721). (**B–E**) Fraction of swimmers (**B**), swimming speed (**C**), chemotactic velocity (**D**), and chemotactic bias (**E**) for MG1655 carrying either empty vector pTrc99A or pTrc99A-TIs induced by indicated concentrations (**B**, **C**) or 50 µM (**D**, **E**) of IPTG. See Methods for details. Values represent the means and standard deviations of three biological replicates. Statistical significance was determined using unpaired two-tailed Student's *t* test (*P* values from left to right: ns = 0.36, *$P = 0.0067$, ***$P = 0.0003$ (**B**); ns = 0.1903, **$P = 0.0029$, ***$P = 0.0002$ (**C**); ns = 0.12 (**D**); ns = 0.74 (**E**). Source data are available online for this figure.

© The Author(s)

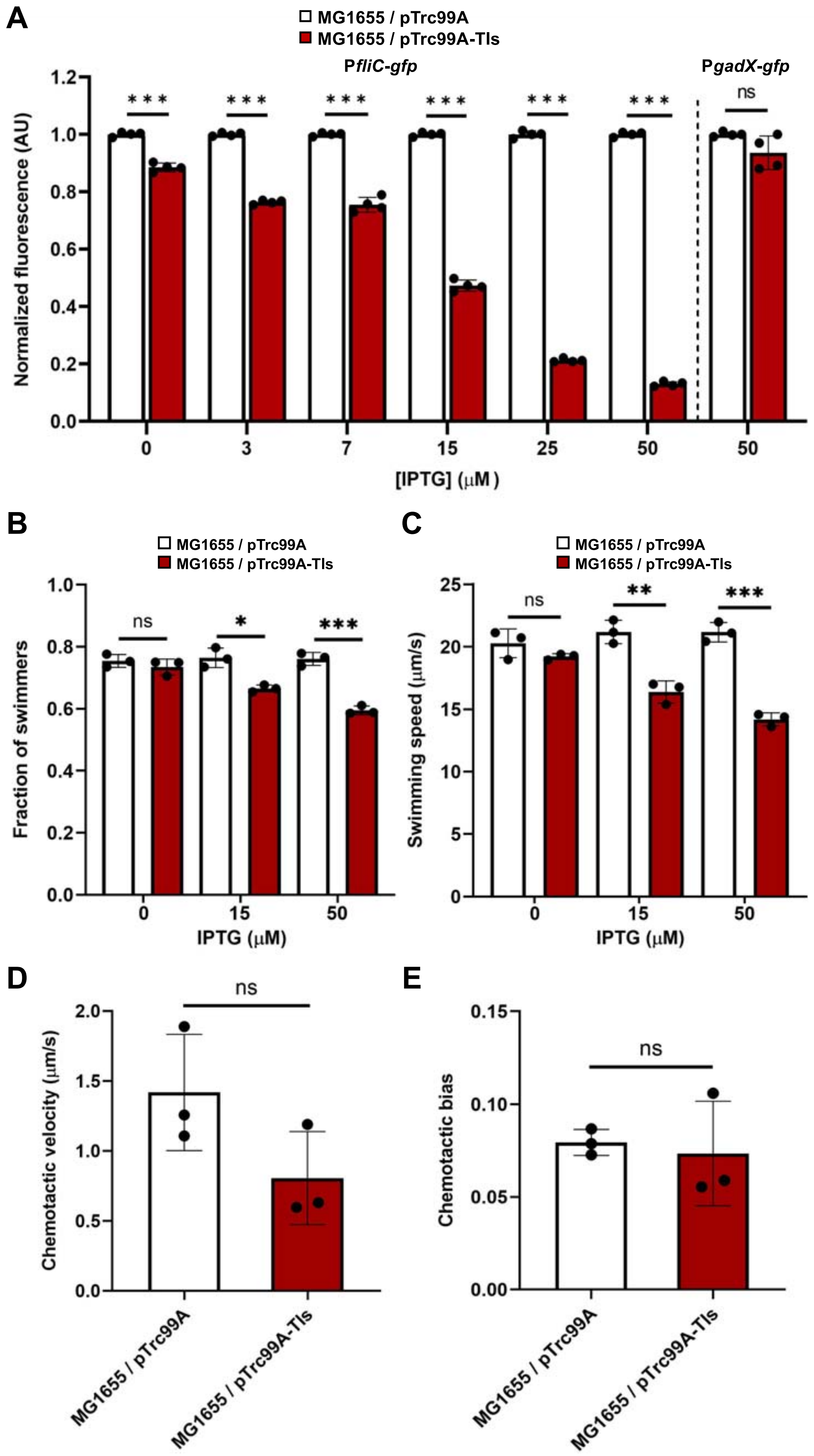
A
MG1655 / pTrc99A
MG1655 / pTrc99A-Tls
PfliC-gfp
PgadX-gfp
Normalized fluorescence (AU)
***
ns
[IPTG] (μM)
0
3
7
15
25
50
50
B
MG1655 / pTrc99A
MG1655 / pTrc99A-Tls
Fraction of swimmers
IPTG (μM)
ns
*
***
C
MG1655 / pTrc99A
MG1655 / pTrc99A-Tls
Swimming speed (μm/s)
IPTG (μM)
ns
**
***
D
Chemotactic velocity (μm/s)
ns
MG1655 / pTrc99A
MG1655 / pTrc99A-Tls
E
Chemotactic bias
ns
MG1655 / pTrc99A
MG1655 / pTrc99A-Tls

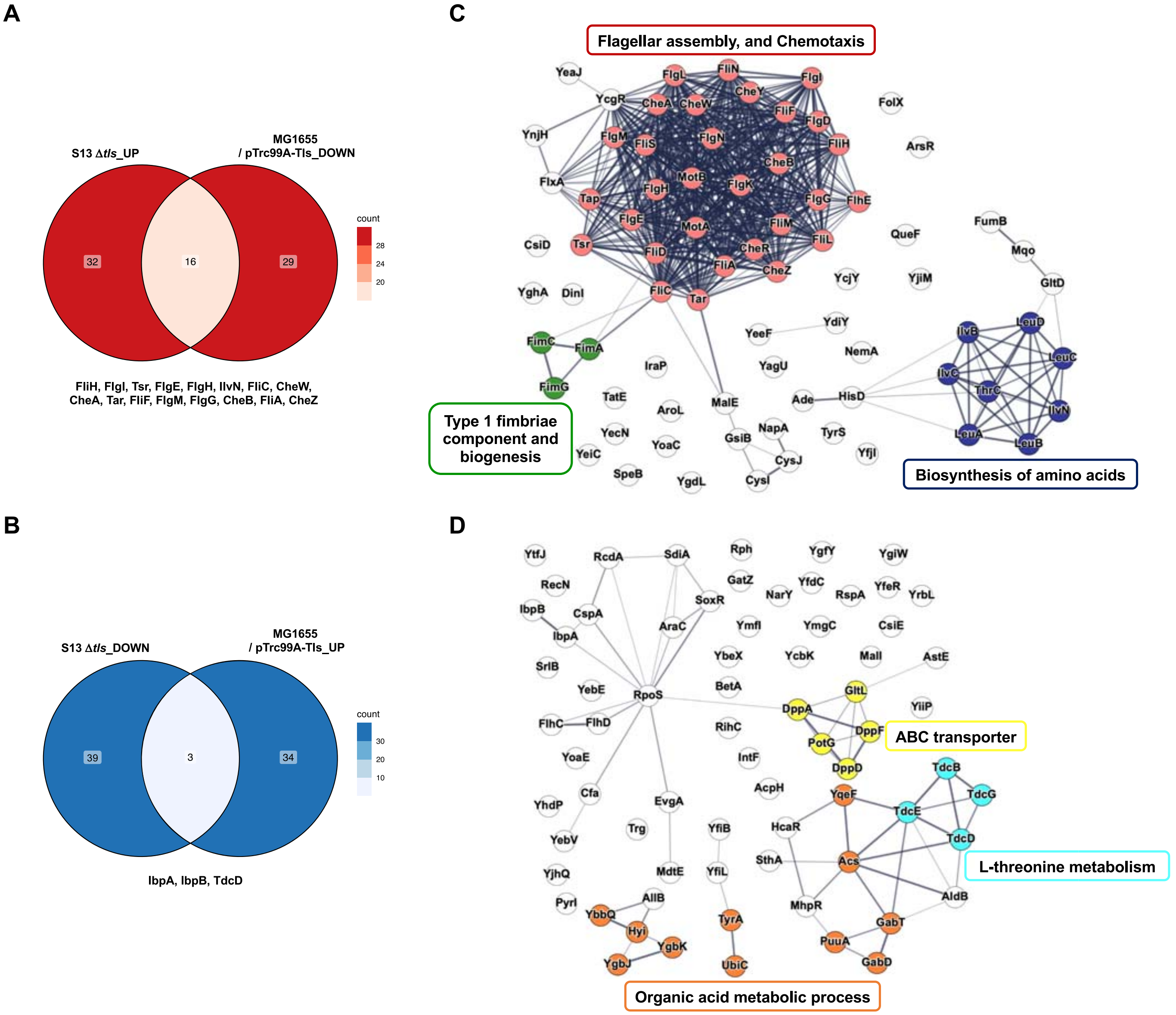

Figure EV2. Downregulation of class 2 and class 3 flagellar proteins by Tls.

(**A**) Venn diagram of proteins upregulated in *E. coli* S13 upon deletion of *tls* and downregulated in *E. coli* MG1655 upon expression of Tls from pTrc99A-Tls at 15 μM IPTG induction. (**B**) Venn diagram of proteins downregulated in *E. coli* S13 upon deletion of *tls* and upregulated in *E. coli* MG1655 upon expression of Tls from pTrc99A-Tls at 15 μM IPTG induction. The protein groups shared by both strains are shown below of each Venn diagrams, and all proteins identified in both strains are listed in Appendix Tables S2–S5. (**C**, **D**) The STRING diagram of the clustering of proteins downregulated (**C**) or upregulated (**D**) by Tls in one or both strains. For the downregulated proteins, the clusters of flagellar assembly and chemotaxis, biosynthesis of amino acids, and type 1 fimbriae component and biogenesis are highlighted in red, blue, and green, respectively. For the upregulated proteins, the clusters of ABC transporter, L-threonine metabolism, and organic acid metabolic process are highlighted in yellow, cyan, and orange, respectively. The thickness of the lines indicates the strength of data support in STRING.

 © The Author(s)

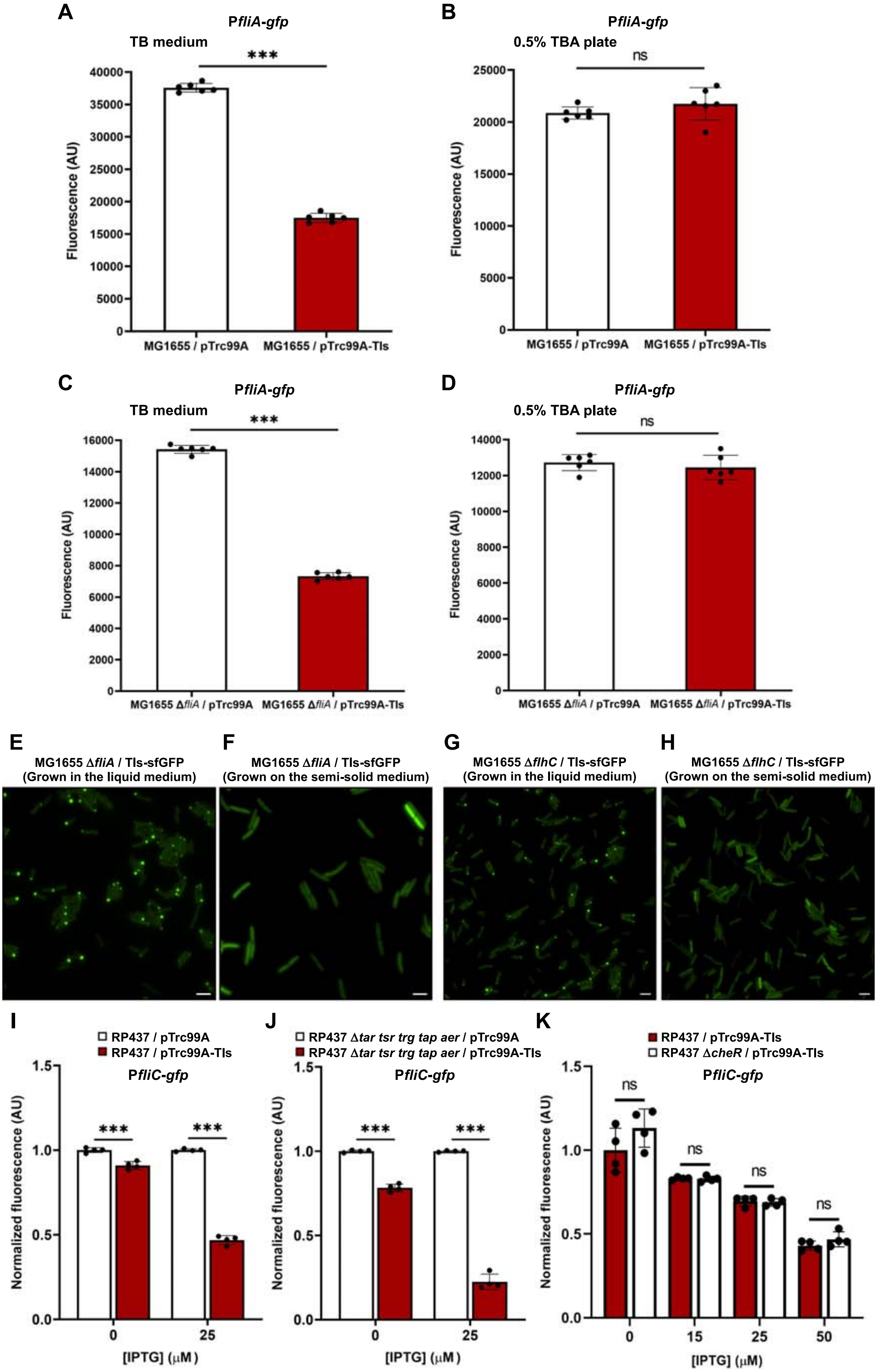

A
PfliA-gfp
TB medium
***
Fluorescence (AU)
MG1655 / pTrc99A
MG1655 / pTrc99A-Tls
B
PfliA-gfp
0.5% TBA plate
ns
C
PfliA-gfp
TB medium
***
MG1655 ΔfliA / pTrc99A
MG1655 ΔfliA / pTrc99A-Tls
D
PfliA-gfp
0.5% TBA plate
ns
E
MG1655 ΔfliA / Tls-sfGFP
(Grown in the liquid medium)
F
MG1655 ΔfliA / Tls-sfGFP
(Grown on the semi-solid medium)
G
MG1655 ΔflhC / Tls-sfGFP
(Grown in the liquid medium)
H
MG1655 ΔflhC / Tls-sfGFP
(Grown on the semi-solid medium)
I
RP437 / pTrc99A
RP437 / pTrc99A-Tls
PfliC-gfp
Normalized fluorescence (AU)
[IPTG] (μM)
J
RP437 Δtar tsr trg tap aer / pTrc99A
RP437 Δtar tsr trg tap aer / pTrc99A-Tls
PfliC-gfp
K
RP437 / pTrc99A-Tls
RP437 ΔcheR / pTrc99A-Tls
PfliC-gfp
ns

Figure EV3. Surface-sensitive regulation of flagellar gene expression by Tls in chemotaxis components-independent manner.

(**A–D**) Fluorescence levels of P*fliA-gfp* at log phase normalized by OD_{600} in MG1655 (**A**, **B**) and MG1655 Δ*fliA* (**C**, **D**) cells carrying either empty pTrc99A vector or pTrc99A-Tls expression plasmid induced with 15 µM IPTG, grown in liquid TB medium (**A**, **C**) or on the surface of 0.5% TB agar (TBA) plate (**B**, **D**). Values represent the means and standard deviations of a minimum of three biological replicates. Statistical significance was determined using unpaired two-tailed Student's *t* test. The *P* values are denoted as ns ($P > 0.05$), * ($P < 0.05$), ** ($P < 0.005$), *** ($P < 0.001$). (***$P = 2.00\text{E-}13$ (**A**); ns = 0.2208 (**B**); ***$P = 4.90\text{E-}14$ (**C**); ns = 0.43 (**D**)). (**E–H**) Cellular localization of Tls-sfGFP in MG1655 Δ*fliA* (**E**, **F**) or MG1655 Δ*flhC* (G and H) cells grown either in the liquid TB medium (**E**, **G**) or on 0.5% TBA plate (**F**, **H**). Representative images are shown from three biological replicates. Scale bars, 3 µm. (**I–K**) Fluorescence levels of P*fliC-gfp* normalized by OD_{600} values in RP437 (**I**) and its receptor-less (**J**) or *cheR* (**K**) mutant carrying either empty vector pTrc99A or pTrc99A-Tls induced with indicated concentrations of IPTG. Measurements were performed in the log phase of growth in a plate reader. Values represent the means and standard deviations of a minimum of three biological replicates, in each case normalized to the reporter activity in the reference strain (RP437/pTrc99A, RP437 Δ*tar tsr trg tap aer*/pTrc99A or RP437/pTrc99A-Tls). Statistical significance was determined using unpaired two-tailed Student's *t* test (*P* values from left to right: ***$P = 0.0005$, ***$P = 1.90\text{E-}08$ (**I**); ***$P = 1.50\text{E-}06$, ***$P = 4.60\text{E-}08$ (**J**); ns = 0.18, ns = 0.78, ns = 0.67, ns = 0.20 (**K**). Source data are available online for this figure.

 © The Author(s)

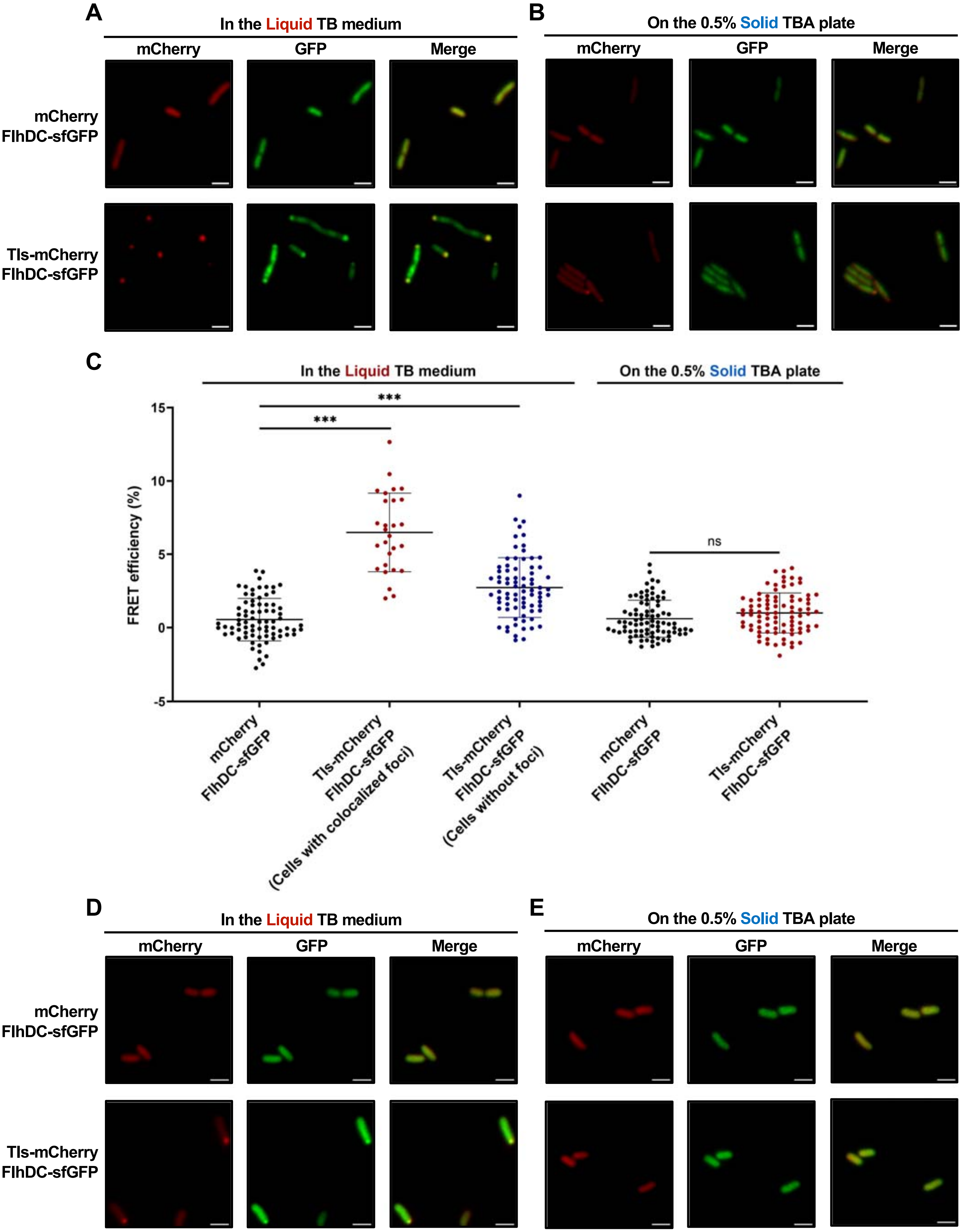
A
In the Liquid TB medium
mCherry
GFP
Merge
mCherry
FlhDC-sfGFP
Tls-mCherry
FlhDC-sfGFP
B
On the 0.5% Solid TBA plate
mCherry
GFP
Merge
C
In the Liquid TB medium
On the 0.5% Solid TBA plate
***
***
ns
FRET efficiency (%)
15
10
5
0
-5
mCherry FlhDC-sfGFP
Tls-mCherry FlhDC-sfGFP (Cells with colocalized foci)
Tls-mCherry FlhDC-sfGFP (Cells without foci)
mCherry FlhDC-sfGFP
Tls-mCherry FlhDC-sfGFP
D
In the Liquid TB medium
mCherry
GFP
Merge
mCherry
FlhDC-sfGFP
Tls-mCherry
FlhDC-sfGFP
E
On the 0.5% Solid TBA plate
mCherry
GFP
Merge

Figure EV4. Interaction of Tls with FlhDC.

(**A**, **B**) Localization of Tls-mCherry or mCherry alone and FlhDC-sfGFP in MG1655 cells grown either in liquid TB medium (**A**) or on 0.5% TBA plate (**B**), as described in Fig. 6F. The mCherry channel, GFP channel, and merged images are shown. Representative images are shown from three biological replicates. Scale bars, 2 µm. (**C**) FRET measurements of complex formation between FlhDC-sfGFP and Tls-mCherry, co-expressed in S13 cells grown either in liquid TB medium or on 0.5% TBA plate, as indicated. Co-expressed mCherry was used as a negative control. FlhDC-sfGFP expression was induced with 0.002% L-arabinose in liquid TB and with 0.004% on 0.5% TBA plate. Tls-mCherry expression was respectively induced with 15 µM or 30 µM IPTG and mCherry expression was respectively induced with 40 µM or 200 µM IPTG. FRET efficiency was determined by acceptor photobleaching in individual cells as described in the Methods. Symbols represent the FRET values in individual cells, measured in three biological replicates (7–33 cells per replicate), with the means and standard deviations being indicated. For liquid-grown cells, the distinction was made between cells that exhibit colocalized foci of Tls-mCherry and FlhDC-sfGFP and those that do not. No colocalized foci were observed in surface-grown cells. Statistical significance was determined using unpaired two-tailed Student's *t* test. The *P* values are denoted as ns ($P > 0.05$), * ($P < 0.05$), ** ($P < 0.005$), *** ($P < 0.001$). (*P* values from left to right: ***$P = 6.76E\text{-}27$, ***$P = 1.41E\text{-}12$, ns = 0.05). (**D**, **E**) Localization of mCherry only, or of Tls-mCherry and FlhDC-sfGFP in S13 cells grown either in liquid TB medium (**D**) or on 0.5% TBA plate (**E**), as described in (**C**). The mCherry channel, GFP channel, and merged images are shown. Representative images are shown from two biological replicates. Scale bars, 2 µm. Source data are available online for this figure.

© The Author(s)

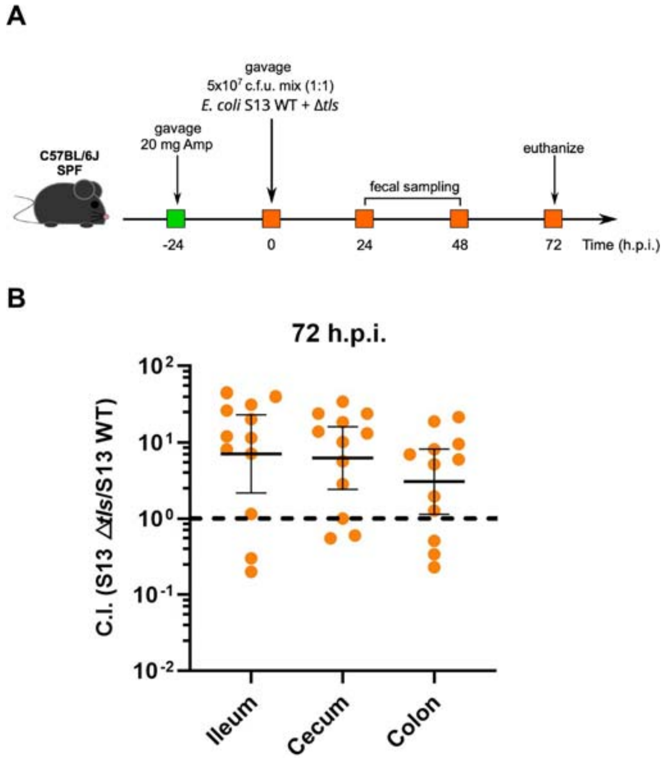

Figure EV5. Testing role of Tls in gut proliferation.

(**A**) Experimental scheme of competitive oral inoculation. C57BL/6J SPF mice were pre-treated with 20 mg of ampicillin by oral gavage 24 h before infection with *E. coli* (1:1 mix of S13 WT and Δ*tls* strains). Feces were collected at 24 and 48 h.p.i., and mice were euthanized at 72 h.p.i. (**B**) The CI values of Δ*tls* in Δ*tls*/WT competitive infection along the gut at 72 h.p.i. Analyses were performed using 12 mice (individual dots). The line depicts the geometric mean +/− 95% CI. The dashed line indicates the CI value of 1. Source data are available online for this figure.

© The Author(s)
